# Translation and cross-cultural adaptation of the MISSCARE Survey-Ped into Brazilian Portuguese

**DOI:** 10.1590/0034-7167-2023-0060

**Published:** 2024-07-19

**Authors:** Julia Silva Del Bello, Kiana Alexandra Rei Gray, Mavilde da Luz Gonçalves Pedreira

**Affiliations:** I Universidade Federal de São Paulo. São Paulo, São Paulo, Brazil; II Conselho Nacional de Desenvolvimento Científico e Tecnológico. Brasília, Distrito Federal, Brazil

**Keywords:** Pediatric Nursing, Patient Safety, Nursing Care, Validation Study, Workload, Enfermería Pediátrica, Seguridad del Paciente, Atención de Enfermería, Estudio de Validación, Carga de Trabajo

## Abstract

**Objectives:**

to translate and cross-culturally adapt the MISSCARE Survey-Ped for use in Brazil.

**Methods:**

a methodological study proposed by translation, synthesis of translations, back-translation, assessment by a committee of experts and pre-testing with the target population.

**Results:**

two direct translations of the instrument were carried out, followed by a consensual version between them. This synthetic version was back-translated and analyzed by a committee of five experts in pediatric nursing and patient safety, obtaining a Content Validity Index (CVI) of 0.95 and Cronbach’s alpha of 0.804. The final version was sent for pre-testing with 254 Brazilian pediatric nurses, with 44 (17.3%) analyzing the instrument for understanding (CVI 0.866; Content Validity Ratio (CVR) 0.773), relevance (CVI 0.931; CVR 0.864) and relevance (CVI 0.977; CVR 0.955).

**Conclusions:**

the MISSCARE Survey-Ped *Brasil* was considered suitable for application in pediatric nurses’ clinical practice in the country.

## RESUMEN

**Objetivos:**

realizar la traducción y adaptación transcultural del instrumento MISSCARE Survey-Ped para su uso en Brasil.

**Métodos:**

estudio metodológico propuesto mediante traducción, síntesis de traducciones, retrotraducción, evaluación por comité de expertos y pretest con la población objetivo.

**Resultados:**

se realizaron dos traducciones directas del instrumento, seguidas de una versión consensuada entre ellas. Esta versión sintética fue retrotraducida y analizada por un comité de cinco expertos en enfermería pediátrica y seguridad del paciente, obteniendo un Índice de Validez de Contenido (IVC) de 0,95 y alfa de Cronbach de 0,804. La versión final fue enviada para prueba previa con 254 enfermeros pediátricos brasileños, y 44 (17,3%) analizaron el instrumento por su comprensión (CVI 0,866; Ratio de Validez de Contenido (CVR) 0,773), relevancia (CVI 0,931; RVC 0,864) y relevancia (CVI 0,977; RVC 0,955).

**Conclusiones:**

el MISSCARE Survey-Ped fue considerado adecuado para su aplicación en la práctica clínica de los enfermeros pediátricos del país.

## INTRODUCTION

Reducing the possibility of errors and violations capable of generating adverse events for patients during healthcare provision is a pillar for promoting patient safety^(1)^. However, a healthcare system cannot be organized without considering that human beings are prone to error. Instead of seeking perfection, patient safety culture aims to identify errors, manage risks, learn from errors and omissions, aiming to institute prevention measures that promote safety for patients and the system as a whole^(1-2)^.

The typology and scope of errors in the healthcare sector are continually being known through research and dissemination of data from notifications encouraged by the World Health Organization and Ministry of Health health security policies^(3)^. Knowledge about situations that compromise patient safety generates opportunities for establishing control and prevention measures that make the healthcare system increasingly reliable, through the remodeling of structures, technologies and interaction processes between the healthcare system, healthcare professionals and patients and families^(4)^.

Errors and adverse events that affect children may differ from those that occur in adults due to characteristics known as 4Ds: demographics; development; dependence; and different health conditions^(5-6)^. In the same care environment, healthcare professionals care for newborns to adolescents, with different needs, types of care and service technologies, and may require knowledge of different specialties to care for neurological, respiratory, cardiovascular, gastrointestinal, urological problems, among others, demanding extensive knowledge from the team in an increasingly specialized area^(4)^. Medication errors stand out in pediatrics, with an estimated proportion of 1.8 to 2.96 errors per 100 discharges in the United States^(5-7)^.

Scientific and ethical nursing care is based on providing the right assistance, in the right way, for the right person and at the right time, based on the best scientific information available to meet the full and individual needs of patients and their family^(8)^. However, the systems’ constant operational failures divert nurses’ actions towards the momentary resolution of difficulties. With such demands, which often go beyond what they are responsible for, nurses are unable to focus on attention to human beings’ individuality and comprehensiveness, delegating excessive activities to the technical level and compromising the ability to assess the nursing care provided^(9)^.

Therefore, amid multiple demands and limited resources, nurses can shorten care, delay it or even omit it. Concerning errors, there are acts of commission (doing something wrong) and acts of omission (failing to do the right thing). Although errors of omission are more common to be ignored, their consequences can constitute a relevant threat to patient safety^(10-11)^. In definition, nursing care missed refers to any aspect of patient care that has been omitted (partially or completely) or delayed^(12-13)^.

To intervene in order to increase safety and, consequently, quality of care, nurses must first identify the factors that predispose the occurrence of nursing care omissions, the barriers that prevent care provision and opportunities for improvement.

Kalisch and Williams^(12)^ developed an instrument called The Missed Nursing Care Survey, the MISSCARE Survey. The tool is divided into three parts, the first being composed of nurses’ demographic data and job satisfaction. Subsequently, Part A presents items relating to care not performed, and Part B covers the reasons for not carrying out such care, containing 24 and 17 items, respectively. Data is measured using a four-point Likert scale, which in Part A varies from “rarely forgotten” to “always forgotten” and, in Part B, from “not a reason” to “significant reason”. Part A and Part B can be applied independently^(12-13)^.

In 2012, the MISSCARE Survey was adapted and validated for use in Brazil, under the name MISSCARE-*BRASIL* ^(10)^. The aim of making this instrument available in Brazil is to use tools capable of assessing nursing care omission, tending to favor the identification of solutions for the phenomenon in question and improving clinical practice^(10)^. To date, there are no other instruments in nationally that propose to assess this phenomenon empirically. However, the instrument does not cover pediatric specificities, which reveals a gap in knowledge of neglected care within Brazilian pediatric nursing.

In 2015, Tubbs-Cooley *et al*.^(14)^ explored nursing care not provided in the neonatal context. To do this, they needed to adapt the original version of MISSCARE Survey considering specific characteristics of newborns, who require different nursing care as well as their planning when compared to adults. This also applies to pediatric hospitals, which differ from both the adult and neonatal settings, and a new version for this population was developed in Italy in 2018, the MISSCARE Survey-Ped^(15)^.

The process of adapting the instrument ended with 29 pediatric nursing care items in Section A and 17 reasons for omission in Section B, considering specific aspects of a pediatric environment, such as the constant presence of family members or caregivers who are actively involved in the care process^(9,15)^. The instrument was validated with a good Content Validity Index (CVI) (0.88) and Cronbach’s alpha (0.912; 0.82; 0.807)^(15)^.

Missed care in pediatrics is of particular concern, given the vulnerability and specificities of this population, as they are less capable of promoting their own safety, and negative results can seriously compromise their health. Therefore, this study proposes to translate and cross-culturally adapt the MISSCARE Survey-Ped for use in Brazil.

## OBJECTIVES

To translate and cross-culturally adapt the MISSCARE Survey-Ped for use in Brazil.

## METHODS

### Ethical aspects

Authorization to use the instrument in Brazil as well as its translation and adaptation was obtained from the author who developed the MISSCARE Survey-Ped. The research was approved by the *Universidade Federal de São Paulo* Research Ethics Committee (UNIFESP-REC), and all study participants signed the Informed Consent Form (ICF).

### Study design, period and location

This is a methodological study consisting of five stages, such as translation, synthesis of translations, back-translation, assessment by a committee of experts and pre-testing of cross-cultural adaptation with the target population, based on COnsensus-based Standards for the selection of health Measurement INstruments (COSMIN) recommendations^(16)^.

Initially, direct translation of the instrument was carried out by two independent translators with knowledge of the original language and the target language. It was decided that one of the translators should be a nurse and be aware of the objectives of the study and the concepts involved (TA), whereas the other should not be a nurse nor aware of the objectives of the study (TB), reflecting the language used in the lay population, as per recommendations of Beaton *et al*.^(17)^.

The translations obtained were analyzed by a professional with clinical and research knowledge in pediatric nursing and patient safety as well as advanced knowledge of English. This consensual version was forwarded to the initial translators for agreement on synthesis translation (ST).

Back-translation was implemented with the participation of two bilingual translators (BTA and BTB), native speakers of the original language and non-experts in the area of study, in an attempt to control bias and help find errors or inconsistencies in translation. All translators carried out the activity independently. The BTA and BTB versions were analyzed by the study authors until consensus was reached on identified consistencies.

A committee of five experts, as recommended by Lynn^(18)^, composed of a team of nurses specializing in pediatric nursing, had to consolidate all the versions obtained (TA, TB, ST, BTA, BTB) and developing the version pre-final of the instrument, considering semantic equivalence (relating to the meaning of words in vocabulary and grammar), idiomatic equivalence (i.e., use of colloquialisms), experimental equivalence (which refers to situations involved in the cultural context) and conceptual equivalence (which explores the different ways a concept can be understood)^(17)^.

This final version of MISSCARE Survey-Ped in Brazilian Portuguese was analyzed in a cross-cultural adaptation study. Pediatric nurses from Brazil, from different regions and levels of training, were invited to analyze understanding, relevance and relevance to pediatric nursing and patient safety.

All data was collected virtually between November 2021 and July 2022.

### Sample and inclusion criteria

The recruitment of nurses participating in the research occurred from a search in curriculums in the Brazilian National Council for Scientific and Technological Development (CNPq - *Conselho Nacional de Desenvolvimento Científico e Tecnológico*) *Lattes* Platform, list of members of the Brazilian Society of Pediatric Nurses, contacts of the nursing research group on patient safety, pediatric intensive care and intravenous therapy in pediatrics (SEGTEC - Safety, Technology and Care) at UNIFESP and recognition as an expert in pediatric nursing or patient safety.

The selection of the five members for the committee included Brazilian nursing professionals, with advanced knowledge of English, clinical and/or research experience in pediatric nursing and/or patient safety. It is noteworthy that four (80.0%) nurses held a PhD degree and one (20.0%) hold a postdoctoral degree.

The decision on sample size for applying pre-testing considered the approach of Beaton *et al*.^(17)^, suggesting between 20 and 40 people from the target group. In total, 254 Brazilian nurses were invited to apply pre-testing through an invitation letter sent by email, and 44 (17.3%) participated in the research.

### Study protocol

Data collection took place virtually and in two stages. The first was developed between November 2021 and May 2022, involving the processes of translation, back-translation and analysis by a committee of experts. For the latter, a form was created in the Research Electronic Data Capture (REDCap)^®^, containing the ST version of the instrument to fill in semantic, idiomatic, experimental and cultural equivalences, using the Delphi technique for this phase of research. Each item presented could be assessed as “not equivalent”, “undecided” or “equivalent”, generating a Likert scale that scored -1, 0, 1, respectively. In cases of -1 or 0, experts should express suggestions for changes to the assessed item. The other versions (original, TA, TB, BTA and BTB) were also made available to experts for consultation.

The second stage of collection took place between June and July 2022 and began after the instrument was validated by the committee. The form for Brazilian nurses was created on Google Forms^®^. Each participant was asked to analyze the instrument based on or simulating a shift or a moment in which they carried out, supervised, experienced or reflected on daily nursing practice in the care of newborns, children, adolescents and their families, highlighting nursing care not performed. At the end of Section A and Section B, nurses could make considerations regarding adequacy or improvement. At the end of the two sections of MISSCARE Survey-Ped, an overall assessment was requested, according to a Likert scale that ranged from 1 to 5 points, considering “totally disagree”, “partially disagree”, “neither agree nor disagree”, “partially agree” and “totally agree”. In case of disagreement, nurses were asked to express suggestions for improvement.

Demographic variables to identify study participants were also collected (age, sex, level of academic training, city and state where they work, length of professional experience in years, length of experience in pediatric nursing, area of activity, work in an institution public or private, work shift and daily working hours), in addition to information related to job satisfaction (how satisfied you are and how you feel in the work environment), as proposed in the original MISSCARE.

### Analysis of results, and statistics

The results were imported into a Microsoft Excel^®^ spreadsheet and analyzed by the study authors. In order to quantify the degree of agreement between experts, equivalences were measured using CVI^(19)^. The minimum value of CVI was considered 0.80 or 80.0% per item. Items that did not reach the minimum value were subjected to reassessment in a second round using the Delphi technique until consensus was reached by the committee.

Cronbach’s alpha was calculated to analyze internal consistency in the first and second round of expert assessment.

In relation to content analysis in the instrument’s overall assessment, the Content Validity Ratio (CVR) was calculated, which measures the agreement among assessing nurses on the importance of the item assessed^(20-21)^. In the present study, 44 nurses participated, requiring a minimum CVR value of 0.29^(21-22)^.

## RESULTS

The TA and TB versions achieved close results, and ST was finalized after adjustments. BTA and BTB indicated fidelity to the original content and then ST was sent for analysis by experts. Concerning the instrument title, it was decided to maintain it in English, plus the designation of the translation as “MISSCARE Survey-Ped *Brasil*”.

Considering the instrument items, titles and subtitles, 50 items were analyzed. In the first round of evaluation by the expert committee, five (10.0%) and seven (14.0%) items relating to Sections A and B, respectively, were modified according to experts’ suggestions, as they obtained CVI<80.0%. The other items obtained CVI > 80.0%, and the instrument’s CVI in the first round was 89.0%, as shown in Tables 1 and 2. Cronbach’s alpha in the first round was 0.826, indicating good consistency.

**Table 1:** First round of the Delphi technique for assessing the translation of Section A of MISSCARE Survey-Ped into Brazilian Portuguese by a committee of experts according to semantic equivalence, idiomatic equivalence, experimental equivalence, cultural equivalence

Analyzed items	Score, mean CVI^*^ (%)				
	SE	IE	EE	CE	Total
*Seção “A” - Atividades de cuidados de enfermagem não realizadas*	4(80.0)	3(60.0)	2(40.0)	2(60.0)	2.75(55.0)
*Participação na visita clínica diária à beira leito*	3(60.0)	3(60.0)	3(60.0)	3(60.0)	3(60.0)
*Deambulação 3 vezes ao dia ou de acordo com o plano de cuidados de enfermagem, se as condições clínicas permitirem*	5(100.0)	5(100.0)	4(80.0)	5(100.0)	4.75(95.0)
*Avaliação da eficácia da medicação*	4(80.0)	4(80.0)	5(100.0)	5(100.0)	4.5(90.0)
*Mudança de decúbito da criança a cada 2 horas ou conforme prescrito*	5(100.0)	5(100.0)	5(100.0)	5(100.0)	5(100.0)
*Cuidados orais*	3(60.0)	3(60.0)	2(40.0)	4(80.0)	3(60.0)
*Envolvimento dos pais nos cuidados com a criança*	4(80.0)	4(80.0)	4(80.0)	4(80.0)	4(80.0)
*Educação do paciente e família*	5(100.0)	5(100.0)	4(80.0)	5(100.0)	4.75(95.0)
*Discussão com a criança e sua família sobre planos de alta e cuidados no domicílio*	5(100.0)	5(100.0)	5(100.0)	5(100.0)	5(100.0)
*Promoção do desenvolvimento neuroevolutivo, de acordo com a idade e condições clínicas da criança (por exemplo, cuidados neonatais, desenvolvimento cognitivo e relacional da criança ou do adolescente)*	5(100.0)	4(80.0)	4(80.0)	5(100.0)	4.5(90.0)
*Avaliação da dor e intervenções farmacológicas ou não farmacológicas, de acordo com protocolos*	5(100.0)	5(100.0)	5(100.0)	5(100.0)	5(100.0)
*Solicitações de medicamentos atendidas dentro de 15 minutos*	5(100.0)	5(100.0)	5(100.0)	5(100.0)	5(100.0)
*Documentação completa com todos os dados necessários*	3(60.0)	5(100.0)	5(100.0)	5(100.0)	4.5(90.0)
*Comunicação de todas as informações relevantes na passagem de plantão ou transferência*	5(100.0)	5(100.0)	5(100.0)	5(100.0)	5(100.0)
*Satisfação das necessidades alimentares, de acordo com as condições clínicas da criança (por exemplo, incentivo a alimentação oral e/ou nutrição do recém-nascido assim que solicitado; incentivo a alimentação apropriada, de acordo com a preferência pessoal)*	5(100.0)	5(100.0)	5(100.0)	5(100.0)	5(100.0)
*Administração de medicamentos 30 minutos antes ou depois do horário programado (por exemplo, horário programado às 20h, administração entre 19h30 e 20h30)*	5(100.0)	4(80.0)	4(80.0)	5(100.0)	4.5(90.0)
*Auxílio a criança nas necessidades de eliminação dentro de 5 minutos após a solicitação (por exemplo, ir com a criança ao banheiro ou fornecer os dispositivos apropriados se estiver no leito)*	4(80.0)	3(60.0)	4(80.0)	4(80.0)	3.75(75.0)
*Resposta à luz de chamada, à solicitação de intervenção ou alarme é iniciada dentro de 5 minutos (por exemplo, monitores, bombas de infusão, aparelhos de ventilação mecânica)*	5(100.0)	4(80.0)	4(80.0)	5(100.0)	4.5(90.0)
*Apoio emocional à criança e/ou família*	5(100.0)	5(100.0)	5(100.0)	5(100.0)	5(100.0)
*Coleta de exames laboratoriais realizados conforme prescrito*	5(100.0)	5(100.0)	5(100.0)	5(100.0)	5(100.0)
*Higiene corporal e cuidados com a pele*	5(100.0)	5(100.0)	5(100.0)	5(100.0)	5(100.0)
*Avaliação do local de inserção do cateter intravenosos central e do cateter intravenoso periférico segundo protocolos*	4(80.0)	4(80.0)	4(80.0)	5(100.0)	4.25(85.0)
*Cuidados com o local de inserção do cateter intravenoso central e do cateter intravenoso periférico segundo protocolos*	4(80.0)	4(80.0)	4(80.0)	5(100.0)	4.25(85.0)
*Adoção das precauções necessárias para o controle de infecções conforme protocolos (uso de EPIs, desinfecção de dispositivos, isolamento, correto descarte de resíduos)*	5(100.0)	4(80.0)	4(80.0)	5(100.0)	4.5(90.0)
*Monitoramento dos ganhos e perdas de sólidos e líquidos*	5(100.0)	5(100.0)	4(80.0)	5(100.0)	4.75(95.0)
*Avaliação dos sinais vitais de acordo com o plano de cuidados de enfermagem*	5(100.0)	5(100.0)	5(100.0)	5(100.0)	5(100.0)
*Reavaliações direcionadas sobre a condição da criança para avaliar melhorias ou agravos durante o plantão*	5(100.0)	4(80.0)	4(80.0)	5(100.0)	4.5(90.0)
*Higienização das mãos*	5(100.0)	5(100.0)	5(100.0)	5(100.0)	5(100.0)
*Avaliação das atividades realizadas pelo cuidador*	4(80.0)	3(60.0)	3(60.0)	4(80.0)	3.5(70.0)
*Verificação de segurança dos equipamentos e limpeza concorrente do mobiliário realizadas uma vez por plantão ou segundo protocolo (por exemplo, cama, mesa de cabeceira, dispositivos)*	5(100.0)	4(80.0)	5(100.0)	5(100.0)	4.75(95.0)

* *CVI - Content Validity Index; SE - semantic equivalence; IE - idiomatic equivalence; EE - experimental equivalence; CE - cultural equivalence; EPI - equipamento de proteção individual.*

**Table 2:** First round of the Delphi technique for assessing the translation of Section B of MISSCARE Survey-Ped into Brazilian Portuguese by a committee of experts according to semantic equivalence, idiomatic equivalence, experimental equivalence, cultural equivalence

Analyzed items	Score, mean CVI^*^ (%)				
	SE	IE	EE	CE	Total
*Seção “B” - razões para as omissões de cuidados em enfermagem*	5(100.0)	5(100.0)	5(100.0)	5(100.0)	5(100.0)
*Recursos laborais*	5(100.0)	4(80.0)	4(80.0)	4(80.0)	4.25(85.0)
*Desequilíbrio nas atribuições com pacientes*	5(100.0)	5(100.0)	4(80.0)	4(80.0)	4.5(90.0)
*Número inadequado de enfermeiras*	3(60.0)	4(80.0)	4(80.0)	4(80.0)	3.75(75.0)
*Situação de urgência do paciente (por exemplo, piora da condição do paciente)*	5(100.0)	4(80.0)	4(80.0)	4(80.0)	4.25(85.0)
*Aumento inesperado do número e/ou gravidade dos pacientes na unidade*	5(100.0)	5(100.0)	5(100.0)	5(100.0)	5(100.0)
*Número inadequado de técnicos/auxiliares de enfermagem*	5(100.0)	5(100.0)	5(100.0)	5(100.0)	5(100.0)
*Interrupções frequentes*	5(100.0)	5(100.0)	5(100.0)	5(100.0)	5(100.0)
*Comunicação*	5(100.0)	5(100.0)	5(100.0)	5(100.0)	5(100.0)
*Tensão ou barreiras de comunicação com a equipe médica*	4(80.0)	3(60.0)	3(60.0)	4(80.0)	3.5(70.0)
*Falta de colaboração entre membros da equipe (por exemplo, enfermeiras, técnicas/auxiliares de enfermagem e médicos)*	3(60.0)	4(80.0)	4(80.0)	4(80.0)	3.75(75.0)
*Tensão ou barreiras de comunicação na equipe de enfermagem*	4(80.0)	4(80.0)	3(60.0)	4(80.0)	3.75(75.0)
*Tensão ou barreiras de comunicação com outros serviços ou departamentos (por exemplo, banco de sangue, serviço de radiologia, farmácia, etc)*	4(80.0)	4(80.0)	1(20.0)	4(80.0)	3.25(65.0)
*Técnica/auxiliar de enfermagem não comunicou que o cuidado à criança não foi realizado*	3(60.0)	4(80.0)	4(80.0)	4(80.0)	3.75(75.0)
*Inadequada passagem de plantão entre turnos ou na transferência entre unidades*	5(100.0)	5(100.0)	5(100.0)	5(100.0)	5(100.0)
*Outros serviços ou setores não prestaram os cuidados necessários (por exemplo, laboratório de análises, farmácia hospitalar)*	5(100.0)	5(100.0)	3(60.0)	5(100.0)	4.5(90.0)
*Recursos materiais*	5(100.0)	5(100.0)	5(100.0)	5(100.0)	5(100.0)
*Materiais/equipamentos não disponíveis quando necessários (por exemplo, bombas de infusão, instrumentais cirúrgicos)*	5(100.0)	5(100.0)	5(100.0)	5(100.0)	5(100.0)
*Materiais/equipamentos não funcionam corretamente quando necessário*	5(100.0)	5(100.0)	5(100.0)	5(100.0)	5(100.0)
*Medicamentos não disponíveis quando necessários*	5(100.0)	5(100.0)	5(100.0)	5(100.0)	5(100.0)
*Falta de treinamento com equipamento/procedimento/normas*	3(60.0)	4(80.0)	4(80.0)	4(80.0)	3.75(75.0)

* *CVI - Content Validity Index; SE - semantic equivalence; IE - idiomatic equivalence; EE - experimental equivalence; CE - cultural equivalence.*

Of the 12 items that required review after the first round, all were validated in the second round of analysis, and the final CVI of the instrument was 0.95. Cronbach’s alpha for the second round was 0.804, indicating acceptable consistency despite decay from one round to another.

After analysis by experts, pre-testing was applied to 44 nurses. All participants were female, with a mean age of 40 years, working in different areas and regions of the country and with different levels of training (Table 3).

**Table 3:** Data collected in pre-testing with participant characterization

Occupation area	n(%)	Region of origin	n(%)	Training level	n(%)
Hospital care	29(65.9%)	North	0(0%)	PhD	14(31.8%)
Teaching	26(59.0%)	Northeast	6(13.6%)	Specialization/improvement	13(29.5%)
Research	23(52.2%)	Midwest	2(4.5%)	Master’s degree	10(22.7%)
Primary care	5(11.3%)	Southeast	32(72.7%)	Post-doctoral degree	5(11.4%)
Management	6(13.6%)	South	4(9.1%)	*Livre-Docência* (title granted in Brazil that attests to superior quality in teaching and research)	1(2.3%)
Others	2(4.5%)			Undergraduate degree	1(2.3%)

Therefore, 30 nurses (68.1%) work in a public institution; nine (20.4%) work in a private institution; four (9.0%) work in both; and one (2.2%) identified himself as retired/not employed. The majority (68.1%) of participants work during the day and 75.0% of nurses did not report working double shifts. With regard to daily working hours, 21 (47.7%) work eight hours a day; nine (20.0%) selected the option other or not applicable; seven (15.9%) comply with the 12x36 schedule; and seven (15.9%) work six hours or less.

As for job satisfaction, 33 (75.0%) nurses declared to be satisfied; nine (20.4%) are very satisfied; and two (4.5%) are not very satisfied. Thus, 20 (45.5%) participants indicated that they feel more happy/excited/good-humored than sad/discouraged/grumpy at work; four (9.1%) nurses feel more sad/discouraged/grumpy than happy/excited/good-humored; 23 (52.3%) feel more calm/satisfied than tense/stressed; 10 (22.7%) feel more tense/stressed than calm/satisfied.

After presenting the instrument, 42 (95.5%) of nurses identified unperformed nursing care, arranged in Section A. The most frequently highlighted items were “*Verificação de segurança dos equipamentos e limpeza concorrente do mobiliário realizadas uma vez por plantão ou segundo protocolo (por exemplo, cama, mesa de cabeceira, dispositivos)*”, marked 29 (69.0%) times, “*Participação na visita clínica multiprofissional diária à beira leito*”, marked 27 (64.3%) times, “*Cuidados bucais*”, “*Solicitações de medicamentos atendidas dentro de 15 minutos*” and “*Avaliação das atividades atribuídas aos cuidadores*”, marked 22 (52.4%) times each.

In Section B, 43 (97.7%) nurses responded that the reasons for omitted nursing care were related to work resources. The most frequently mentioned items were “*Número inadequado de técnicos/auxiliares de enfermagem*”, with 34 (79.1%) responses, and “*Número inadequado de enfermeiros*”, with 33 (76.7%).

With respect to the causes related to communication, 41 (93.2%) nurses responded and highlighted mainly the items “*Falta de colaboração entre membros da equipe (por exemplo, enfermeiros, técnicos/auxiliares de enfermagem e médicos)*”, with 33 (80.5%) responses, and “*Inadequada passagem de plantão entre turnos ou na transferência entre unidades*”, with 28 (68.3%) responses.

Hence, 40 (90.9%) nurses considered material resources as a reason for omitting nursing care, highlighting in greater numbers the items “*Materiais/equipamentos não funcionam corretamente quando necessário*”, with 29 (72.5%) responses, and “*Materiais/equipamentos não disponíveis quando necessários (por exemplo, bombas de infusão, instrumentais cirúrgicos)*”, with 28 (70.0%) responses.

In relation to understanding, 29 (65.9%) of nurses completely agreed that the instrument is clear, intelligible and easy to understand; 10 (22.7%) partially agreed; four (9.1%) partially disagreed; and one (2.3%) neither agreed nor disagreed.

When assessing relevance, 28 (63.6%) nurses completely agreed that the instrument covers situations related to nursing care not provided and reasons for omissions; 13 (29.5%) partially agreed; two (4.5%) neither agreed nor disagreed; and one (2.3%) totally disagreed.

It was found that 32 (72.7%) nurses completely agreed in the analysis that the instrument is relevant for identifying nursing care not performed and the reasons for omissions; 11 (25.0%) partially agreed; and one (2.3%) partially disagreed.

According to Table 4, high CVR and CVI values are noted, indicating consistency in evaluators’ responses to the questionnaire assessment items.

**Table 4:** Estimates of the Content Validity Ratio and Content Validity Index in the overall assessment of MISSCARE Survey-Ped *Brasil* regarding understanding, pertinence and relevance

	The instrument is clear, intelligible and easy to understand	The instrument covers situations related to nursing care not provided and reasons for omissions	The instrument is relevant for identifying nursing care not performed and reasons for omissions
CVR^*^	0.773	0.864	0.955
CVT^†^	0.886	0.931	0.977

* CVR - Content Validity Ratio;

† CVI - Content Validity Index.

From statistical analysis using the Quasi-Poisson generalized linear model, it was observed that labor resources increased by an average of 1,290 nursing care described as not performed, as there was a significant relationship between reporting nursing care not performed due to work-related causes (p<0.001).

It was also found that the total nursing care mentioned in Section A is not related to sociodemographic variables. The mean number of reasons indicated in Section B is different between those who work six hours or less and those who answered other/not applicable. Nurses who answered other/not applicable indicated more reasons in Section B.

Working at night increased the number of items marked in Section B in labor resources by a mean of 1.6. Being more happy/excited/good-humored than sad/discouraged/grumpy and more calm/satisfied than tense/stressed the number of items marked in Part B in labor resources decreased by a mean of 1.37.

In the cross-cultural adaptation process, suggestions regarding the terms used stood out. A nurse mentioned that “*In the item “Resposta à luz de chamada, à solicitação de chamada e alarme”, call light is perhaps more understandable as a bell [...]*”. Another suggestion was “*In the item “Documentação completa com todos os dados necessários”, it is not very clear in the translation whether this refers to documents in the medical record or shift handover or handoff [...]*”. However, these items were not revised, as they were mentioned by only one nurse (2.2%).

Based on suggestions of participating nurses regarding Section A, Section B and the overall assessment of the instrument, the item “*Desequilíbrio nas atribuições com pacientes*” needed to be revised and changed to “*Desequilíbrio no dimensionamento de pacientes*”, as this issue was mentioned by two nurses (4.5%) who expressed doubt about the meaning of the phrase, and the suggestion was understood as pertinent. thus, the latest version of MISSCARE Survey-Ped *Brasil* was finalized, as shown in Chart 1.

**Chart 1:** Versions of the MISSCARE Survey-Ped *Brasil* with items changed according to suggestions highlighted in bold

Initial version of MISSCARE Survey-Ped *Brasil*	Final version of MISSCARE Survey-Ped *Brasil*	Analysis of modifications
***Seção “A” - Atividades de cuidados de enfermagem não realizadas***	***Seção “A” - Cuidados de enfermagem não realizados***	*The word “actividades” was deleted because “atividades de” followed by “cuidados” was considered redundant*
***Participação na visita clínica diária à beira leito***	***Participação na visita clínica multiprofissional diária à beira leito***	*The item was reformulated because it was not clear whether the term “visita” referred to nurses appearing in the patient’s room during a multidisciplinary clinical visit in the context of case handover or whether nurses did not enter the patient’s room during the entire shift.*
*Deambulação 3 vezes ao dia ou de acordo com o plano de cuidados de enfermagem, se as condições clínicas permitirem*	*Deambulação 3 vezes ao dia ou de acordo com o plano de cuidados de enfermagem, se as condições clínicas permitirem*	
*Avaliação da eficácia da medicação*	*Avaliação da eficácia da medicação*	
*Mudança de decúbito da criança a cada 2 horas ou conforme prescrito*	*Mudança de decúbito da criança a cada 2 horas ou conforme prescrito*	
***Cuidados orais***	***Cuidados bucais***	*The word “orais” was replaced by “bucais” as this is closer to what is used in nursing practice.*
*Envolvimento dos pais nos cuidados com a criança*	*Envolvimento dos pais nos cuidados com a criança*	
*Educação do paciente e família*	*Educação do paciente e família*	
*Discussão com a criança e sua família sobre planos de alta e cuidados no domicílio*	*Discussão com a criança e sua família sobre planos de alta e cuidados no domicílio*	
*Promoção do desenvolvimento neuroevolutivo, de acordo com a idade e condições clínicas da criança (por exemplo, cuidados neonatais, desenvolvimento cognitivo e relacional da criança ou do adolescente)*	*Promoção do desenvolvimento neuroevolutivo, de acordo com a idade e condições clínicas da criança (por exemplo, cuidados neonatais, desenvolvimento cognitivo e relacional da criança ou do adolescente)*	
*Avaliação da dor e intervenções* *farmacológicas ou não farmacológicas, de acordo com protocolos*	*Avaliação da dor e intervenções farmacológicas ou não farmacológicas, de acordo com protocolos*	
*Solicitações de medicamentos atendidas dentro de 15 minutos*	*Solicitações de medicamentos atendidas dentro de 15 minutos*	
*Documentação completa com todos os dados necessários*	*Documentação completa com todos os dados necessários*	
*Comunicação de todas as informações relevantes na passagem de plantão ou transferência*	*Comunicação de todas as informações relevantes na passagem de plantão ou transferência*	
*Satisfação das necessidades alimentares, de acordo com as condições clínicas da criança (por exemplo, incentivo a alimentação oral e/ou nutrição do recém-nascido assim que solicitado; incentivo a alimentação apropriada, de acordo com a preferência pessoal)*	*Satisfação das necessidades alimentares, de acordo com as condições clínicas da criança (por exemplo, incentivo a alimentação oral e/ou nutrição do recém-nascido assim que solicitado; incentivo a alimentação apropriada, de acordo com a preferência pessoal)*	
*Administração de medicamentos entre 30 minutos antes ou depois do horário programado (por exemplo, horário programado às 20h, administração entre 19h30 e 20h30)*	*Administração de medicamentos entre 30 minutos antes ou depois do horário programado (por exemplo, horário programado às 20h, administração entre 19h30 e 20h30)*	
***Auxílio a criança nas necessidades de eliminação dentro de 5 minutos após a solicitação (por exemplo, ir com a criança ao banheiro ou fornecer os dispositivos apropriados se estiver no leito)***	***Auxílio a criança nas necessidades de eliminação dentro de 5 minutos após a solicitação (por exemplo, ir com a criança ao banheiro ou fornecer os dispositivos apropriados se estiver restrita no leito)***	*The term “restrita no leito” was added, as it is understood that all hospitalized children will be in a bed.*
*Resposta à luz de chamada, à solicitação de intervenção ou alarme é iniciada dentro de 5 minutos (por exemplo, monitores, bombas de infusão, aparelhos de* *ventilação mecânica)*	*Resposta à luz de chamada, à solicitação de intervenção ou alarme é iniciada dentro de 5 minutos (por exemplo, monitores, bombas de infusão, aparelhos de* *ventilação mecânica)*	
*Apoio emocional à criança e/ou família*	*Apoio emocional à criança e/ou família*	
*Coleta de exames laboratoriais realizados conforme prescrito*	*Coleta de exames laboratoriais realizados conforme prescrito*	
*Higiene corporal e cuidados com a pele*	*Higiene corporal e cuidados com a pele*	
*Avaliação do local de inserção do cateter intravenosos central e do cateter intravenoso periférico segundo protocolos*	*Avaliação do local de inserção do cateter intravenosos central e do cateter intravenoso periférico segundo protocolos*	
*Cuidados com o local de inserção do cateter intravenoso central e do cateter intravenoso periférico segundo protocolos*	*Cuidados com o local de inserção do cateter intravenoso central e do cateter intravenoso periférico segundo protocolos*	
*Adoção das precauções necessárias para o controle de infecções conforme protocolos (uso de EPIs, desinfecção de dispositivos, isolamento, correto descarte de resíduos)*	*Adoção das precauções necessárias para o controle de infecções conforme protocolos (uso de EPIs, desinfecção de dispositivos, isolamento, correto descarte de resíduos)*	
*Monitoramento dos ganhos e perdas de sólidos e líquidos*	*Monitoramento dos ganhos e perdas de sólidos e líquidos*	
*Avaliação dos sinais vitais de acordo com o plano de cuidados de enfermagem*	*Avaliação dos sinais vitais de acordo com o plano de cuidados de enfermagem*	
*Reavaliações direcionadas sobre a condição da criança para avaliar melhorias ou agravos durante o plantão*	*Reavaliações direcionadas sobre a condição da criança para avaliar melhorias ou agravos durante o plantão*	
*Higienização das mãos*	*Higienização das mãos*	
***Avaliação das atividades realizadas pelo cuidador***	***Avaliação das atividades atribuídas aos cuidadores***	*Since “realizar” and “atribuir” are different concepts, an analysis was carried out with the original version of the instrument, understanding the use of the word “atribuídas” as more pertinent to the context.*
*Verificação de segurança dos equipamentos e limpeza concorrente do mobiliário realizadas uma vez por plantão ou segundo protocolo (por exemplo, cama, mesa de cabeceira, dispositivos)*	*Verificação de segurança dos equipamentos e limpeza concorrente do mobiliário realizadas uma vez por plantão ou segundo protocolo (por exemplo, cama, mesa de cabeceira, dispositivos)*	
*Seção “B” - razões para as omissões de cuidados em enfermagem*	*Seção “B” - razões para as omissões de cuidados em enfermagem*	
*Recursos laborais*	*Recursos laborais*	
***Desequilíbrio nas atribuições com*** ***pacientes***	***Desequilíbrio no dimensionamento de pacientes***	*“Atribuições” was not clear and so it was replaced to “dimensionamento”.*
***Número inadequado de enfermeiras***	***Número inadequado de enfermeiros***	*Changed to “enfermeiros” to encompass both sexes.*
*Situação de urgência do paciente (por exemplo, piora da condição do paciente)*	*Situação de urgência do paciente (por exemplo, piora da condição do paciente)*	
*Aumento inesperado do número e/ou gravidade dos pacientes na unidade*	*Aumento inesperado do número e/ou gravidade dos pacientes na unidade*	
*Número inadequado de técnicos/auxiliares de enfermagem*	*Número inadequado de técnicos/auxiliares de enfermagem*	
*Interrupções frequentes*	*Interrupções frequentes*	
*Comunicação*	*Comunicação*	
***Tensão ou barreiras de comunicação com a equipe médica***	***Tensão ou falhas na comunicação com a equipe médica***	*The word “barreiras” was not clear and was therefore replaced with “falhas”.*
***Falta de colaboração entre membros da equipe (por exemplo, enfermeiras, técnicas/auxiliares de*** ***enfermagem e médicos)***	***Falta de colaboração entre membros da equipe (por exemplo, enfermeiros, técnicas/auxiliares de enfermagem e médicos)***	*Changed to “enfermeiros” and “técnicos” to encompass both sexes.*
***Tensão ou barreiras de comunicação na equipe de enfermagem***	***Tensão ou falhas na comunicação na equipe de enfermagem***	*The word “barreiras” was not clear and was therefore replaced with “falhas”.*
***Tensão ou barreiras de comunicação com outros serviços ou departamentos (por exemplo, banco de sangue, serviço de radiologia, farmácia, etc)***	***Tensão ou falhas na comunicação com outros serviços ou departamentos (por exemplo, banco de sangue, serviço de radiologia, farmácia, etc)***	*Same as above.*
***Técnica/auxiliar de enfermagem não comunicou que o cuidado à criança não foi realizado***	***Técnico/auxiliar de enfermagem não comunicou que o cuidado à criança não foi realizado***	*Changed to “técnicos” to encompass both sexes.*
*Inadequada passagem de plantão entre turnos ou na transferência entre unidades*	*Inadequada passagem de plantão entre turnos ou na transferência entre unidades*	
*Outros serviços ou setores não prestaram os cuidados necessários (por exemplo, laboratório de análises, farmácia hospitalar)*	*Outros serviços ou setores não prestaram os cuidados necessários (por exemplo, laboratório de análises, farmácia hospitalar)*	
*Recursos materiais*	*Recursos materiais*	
*Materiais/equipamentos não disponíveis quando necessários (por exemplo, bombas de infusão, instrumentais cirúrgicos)*	*Materiais/equipamentos não disponíveis quando necessários (por exemplo, bombas de infusão, instrumentais cirúrgicos)*	
*Materiais/equipamentos não funcionam corretamente quando necessário*	*Materiais/equipamentos não funcionam corretamente quando necessário*	
*Medicamentos não disponíveis quando necessários*	*Medicamentos não disponíveis quando necessários*	
***Falta de treinamento com equipamento/procedimento/norma***	***Falta de familiaridade com equipamento/procedimento/norma***	*The term “treinamento” was replaced by “familiaridade”, because, although the professional may have been trained, they may not have many opportunities to come into contact with some equipment/procedure/standard.*

## DISCUSSION

Errors of omission are considered phenomena intrinsic to the human cognitive process and are among the most relevant types of incidents in the healthcare system worldwide, due to their frequent occurrence and potential to cause harm to patients. However, there is still little empirical evidence of this phenomenon in pediatric populations^(23)^.

The present study described the process of translating and cross-cultural adapting the MISSCARE Survey-Ped, originally in English, into Brazilian Portuguese, following recommendations in the literature. The process, although complex, is of great importance, because while it preserves the characteristics of the original version, it is adapted considering the heterogeneity of the population and the use of regional terms^(24)^.

The involvement of experts in pediatric nursing and patient safety favored the development of the study, as, with their proven experience in the area, they suggested adjustments that promoted improvements in the synthesis version (ST). Likewise, the participation of pediatric nurses from all regions of Brazil, except the North, with different levels of training, benefited the identification of differences in aspects of nursing practice, when considering the country’s continental proportions. It is noteworthy, however, that the majority of nurses had graduate academic training, reflecting a portion of nurses with greater academic preparation. This result may be related to the low proportion of nurses who agreed to participate in the research, with graduate professionals being more encouraged to participate in scientific research of this nature.

The relationship between identification of care not performed in adult patients observed in the MISSCARE validity study in Brazil^(10)^, compared to pediatric patients, is significant in terms of participation in the multidisciplinary clinical visit, as was also observed in the present study. The organization of the multidisciplinary team’s work process in caring for hospitalized children depends on professionals’ conceptions about meaning of work and team, and their importance, aiming to achieve comprehensive care. Communication failures, work overload, health service flows and processes and interruptions are aspects that weaken care dynamics^(25)^.

Regarding care not carried out in the pediatric context, in a validity study of MISSCARE Survey-Ped for use in Turkey, the main nursing care activities not carried out during the last shift were: administering medications 30 minutes before or after hours schedules; responding to patient requests for prescribed medications within 15 minutes; and involving parents in child care^(26)^. The latter, being specific care for pediatric environments, points to Patientand Family-Centered Care (PFCC).

PFCC implementation in care contexts can result in several actions and strategies that contribute to patient safety, promoting the provision of qualified care that results in a better experience for patients and families in contact with the healthcare system. However, the lack of training of healthcare professionals on establishing effective patient relationships is still an obstacle to be overcome^(9,25)^.

Also noteworthy is the item in Section A that was most frequently highlighted by participating Brazilian nurses as care not performed, “*Verificação de segurança dos equipamentos e limpeza concorrente do mobiliário realizadas uma vez por plantão ou segundo protocolo (por exemplo, cama, mesa de cabeceira, dispositivos)*”, which is directly linked to hospital infection prevention and control. Healthcare-Associated Infections (HAIs) in pediatrics are important adverse events that affect treatment, increasing morbidity, mortality, length of stay, costs and suffering for children and family.

In Section B, which points to the reasons for nursing care omission, the subsection “*recursos laborais*” presented the highest mean comparison of responses. Likewise, in studies carried out with MISSCARE in the United States^(12)^, Turkey^(27)^ and Brazil^(10)^, they also obtained higher scores related to labor resources.

The inadequate number of nursing professionals was cited as the main reason for not providing nursing care in pediatrics. This is consistent with national studies that report the precariousness, inadequacies and dissatisfactions experienced in hospitals by these professionals, involving working conditions, dissatisfaction with salary, increased hours worked and burnout. As a result, workers are forced to request leaves of absence and/or resign, a situation that contributes to the lack of professionals in the area^(28-30)^. Considering that the majority of participating nurses work in public health institutions, this explains the high number of nurses who marked this item.

Both versions, MISSCARE Survey-Ped and MISSCARE Survey-Ped *Brasil*, presented similar results in the validity process: Cronbach’s alpha values were greater than 0.70, considered acceptable for internal consistency and, similarly, the CVI was greater than 0.80, demonstrating good content validity.

### Study limitations

The impossibility of comparing the findings between all Brazilian regions was identified as a limitation of this study, as there was no participation of nurses from the North region. Furthermore, participants’ nursing practice context can modify their assessment of situations that lead to omissions in nursing care. Although carried out in different regions of the country, as the workplace was not a variable considered in this study, nurses allocated to a given location may have contributed more broadly, without necessarily reflecting the reality of that region. Considering that this was a translation and cross-cultural adaptation study, it is also necessary that the MISSCARE Survey-Ped *Brasil* be applied to a larger sample and with factor analysis. This study is under development for practical assessment of the instrument.

### Contributions to nursing, health, or public policies

The advantages of using the instrument are widely described in international literature, highlighting that it can offer information for developing public policies aimed at promoting patient safety, having been assessed by experts. It can also be useful to healthcare institutions in the development of protocols, guidelines or checklists, in order to promote positive professional practice environments, prevent forgetfulness and possible errors of omission during nursing practice. As it is aimed at pediatric nursing, it will cover the specificities of this population, making it possible to improve outcome indicators in the healthcare of children who require hospitalization.

## CONCLUSIONS

With this study, it was possible to translate and cross-culturally adapt the MISSCARE Survey-Ped into Brazilian Portuguese, being considered suitable for use in national health institutions, upon achieving adequate levels of content validation by specialists and nurses in clinical practice.

## Supplementary Material

0034-7167-reben-77-02-e20230060-suppl01

0034-7167-reben-77-02-e20230060-suppl02

0034-7167-reben-77-02-e20230060-suppl03

0034-7167-reben-77-02-e20230060-suppl04

0034-7167-reben-77-02-e20230060-suppl05

## Data Availability

*https://doi.org/10.48331/scielodata.IOBMLO*
