# Supplementary material for: Translation and cross-cultural adaptation of the MISSCARE Survey-Ped into Brazilian Portuguese
Source: Rev Bras Enferm. 2024 Jul 19;77(2):e20230060. doi: 10.1590/0034-7167-2023-0060 (PMC11259437; doi:10.1590/0034-7167-2023-0060)
Supplement: 0034-7167-reben-77-02-e20230060-suppl01 [file 0034-7167-reben-77-02-e20230060-suppl01.pdf]

ID	Idade (anos)	Sexo	Nível de formação acadêmica	Cidade e Estado onde trabalha	Tempo de experiência profissional em anos	Tempo de experiência na área de enfermagem pediátrica	Qual a sua área de atuação? Se necessário, assinale mais de uma opção.	Trabalha em instituição pública ou privada?	Período do turno de trabalho. Assinale mais de uma opção se necessário.
1	60	Fem inino	Doutorado	São Paulo, São Paulo	11 anos ou mais	11 anos ou mais	Assistência Hospitalar, Docência, Pesquisa	Pública	Período diurno
2	25	Fem inino	Graduação em Enfermagem em andam ento	São Paulo/SP	Não tenho experiência na atuação profissional	Não tenho experiência na área	Assistência Hospitalar, Pesquisa	Pública	Período diurno
3	42	Fem inino	Mestrado	Uberlândia MG	11 anos ou mais	11 anos ou mais	Assistência na Atenção Primária, Docência	Pública	Período noturno
4	33	Fem inino	Mestrado	Ribeirão Preto	6 a 10 anos	2 a 5 anos	Assistência Hospitalar, Docência, Pesquisa	Pública	Período diurno
5	36	Feminino	Especialização/aperfeiçoamento	Feira de Santana - Bahia	6 a 10 anos	2 a 5 anos	Assistência Hospitalar, Gestão	Pública	Período diurno
6	40	Feminino	Especialização/aperfeiçoamento	SÃO PAULO/SP	11 anos ou mais	11 anos ou mais	Assistência Hospitalar, Docência, Pesquisa	Pública	Período diurno
7	23	Feminino	Especialização/aperfeiçoamento	São Paulo SP	2 a 5 anos	2 a 5 anos	Assistência Hospitalar	Pública	Período diurno
8	38	Feminino	Mestrado	FORTALEZA - CEARÁ	11 anos ou mais	11 anos ou mais	Docência	Privada	Período diurno
9	46	Feminino	Pós-doutorado	Florianopolis SC	11 anos ou mais	11 anos ou mais	Docência, Pesquisa	Pública	Período diurno
10	34	Feminino	Mestrado	RIO DE JANEIRO - RJ	6 a 10 anos	6 a 10 anos	Assistência Hospitalar, Docência, Pesquisa	Privada	Período diurno, Período noturno
11	33	Fem inino	Doutorado	São Paulo- SP	2 a 5 anos	2 a 5 anos	Docência, Pesquisa	Pública	Período diurno
12	33	Fem inino	Doutorado	Curitiba- PR	6 a 10 anos	6 a 10 anos	Assistência Hospitalar, Pesquisa	Pública	Período noturno
13	59	Fem inino	Pós-doutorado	NITERÓI/RIO DE JANEIRO	11 anos ou mais	11 anos ou mais	Assistência Hospitalar, Assistência na Atenção Primária, Gestão, Docência, Pesquisa	Pública	Período diurno
14	45	Feminino	Especialização/aperfeiçoamento	Ribeirão Preto - SP	11 anos ou mais	6 a 10 anos	Assistência Hospitalar, Outro	Am bas	Período diurno, Período noturno

15	45	Feminino	Especialização/aperfeiçoamento	São Paulo	11 anos ou mais	11 anos ou mais	Assistência Hospitalar	Privada	Período noturno
16	41	Feminino	Especialização/aperfeiçoamento	São Paulo sp	11 anos ou mais	11 anos ou mais	Assistência Hospitalar	Privada	Período noturno
17	45	Feminino	Mestrado	imbituba - santa catarina	11 anos ou mais	11 anos ou mais	Outro	Pública	Período diurno
18	39	Fem inino	Doutorado	Salvador- Bahia	11 anos ou mais	6 a 10 anos	Assistência Hospitalar, Docência	Pública	Período diurno
19	31	Fem inino	Mestrado	São Paulo - SP	6 a 10 anos	6 a 10 anos	Assistência Hospitalar, Docência, Pesquisa	Pública	Período diurno
20	3	Fem	Doutorado	Rio de Janeiro. RJ	6 a 10 anos	6 a 10 anos	Docência, Pesquisa	Pública	Período diurno
21	3 5 1	inino Fem inino	Doutorado	Rio de Janeiro/RJ	11 anos ou mais	11 anos ou mais	Assistência Hospitalar, Docência, Pesquisa	Pública	Período noturno
22	37	Fem inino	Doutorado	RJ - RJ	11 anos ou mais	11 anos ou mais	Assistência Hospitalar, Docência, Pesquisa	Pública	Período diurno
23	52	Feminino	Doutorado	macae- RJ	11 anos ou mais	11 anos ou mais	Docência	Am bas	Período diurno
24	43	Feminino	Especialização/aperfeiçoamento	MACEIÓ-AL	11 anos ou mais	11 anos ou mais	Assistência Hospitalar	Pública	Período diurno, Período noturno
25	54	Feminino	Doutorado	londrina paraná	11 anos ou mais	11 anos ou mais	Assistência Hospitalar, Assistência na Atenção Primária, Docência, Pesquisa	Pública	Período diurno
26	31	Feminino	Especialização/aperfeiçoamento	Campo Grande MS	2 a 5 anos	2 a 5 anos	Assistência Hospitalar	Pública	Período noturno
27	63	Feminino	Pós-doutorado	Cuiabá, MT	11 anos ou mais	11 anos ou mais	Assistência Hospitalar, Assistência na Atenção Primária, Docência, Pesquisa	Aposentado/Não em pregado	Não se aplica
28	43	Fem inino	Doutorado	Rio de Janeiro-RJ	11 anos ou mais	11 anos ou mais	Assistência na Atenção Primária, Pesquisa	Pública	Período diurno
2	50	Fem inino	Pós-doutorado	São Paulo - SP	11 anos ou mais	11 anos ou mais	Docência, Pesquisa	Pública	Período diurno
9	48	Fem inino	Mestrado	São Paulo	11 anos ou mais	11 anos ou mais	Gestão	Privada	Período diurno
3 31 0	47	Fem inino	Pós-doutorado	Belo Horizonte - Minas Gerais	11 anos ou mais	11 anos ou mais	Assistência Hospitalar, Gestão, Docência, Pesquisa	Pública	Período diurno

32	36	Feminino	Doutorado	SÃO PAULO	11 anos ou mais	11 anos ou mais	Docência, Pesquisa	Privada	Período diurno
33	29	Feminino	Especialização/aperfei çoamento	São Paulo	2 a 5 anos	2 a 5 anos	Assistência Hospitalar, Pesquisa	Am bas	Período diurno
34	40	Feminino	Doutorado	São Paulo SP	11 anos ou mais	11 anos ou mais	Docência	Pública	Período diurno
35	29	Fem inino	Mestrado	São Paulo	6 a 10 anos	2 a 5 anos	Assistência Hospitalar, Docência, Pesquisa	Privada	Período diurno
36	52	Fem inino	Livre-Docência	Ribeirão Preto - SP	11 anos ou mais	11 anos ou mais	Gestão, Docência, Pesquisa	Pública	Período diurno
37	44	Fem inino	Doutorado	Salvador- BA	11 anos ou mais	11 anos ou mais	Docência	Pública	Não se aplica
38	33	Fem inino	Mestrado	Feira de Santana-Bahia	6 a 10 anos	6 a 10 anos	Assistência Hospitalar, Gestão	Am bas	Período diurno, Período noturno
39	43	Fem inino	Doutorado	São Paulo	11 anos ou mais	11 anos ou mais	Assistência Hospitalar, Docência, Pesquisa	Privada	Período diurno
40	34	Feminino	Especialização/aperfei çoamento	Campinas, SP	11 anos ou mais	6 a 10 anos	Assistência Hospitalar	Pública	Período diurno
41	47	Feminino	Especialização/aperfei çoamento	Campinas_sp	11 anos ou mais	11 anos ou mais	Assistência Hospitalar	Pública	Período noturno
42	42	Feminino	Mestrado	São Paulo- SP	11 anos ou mais	11 anos ou mais	Docência	Privada	Período diurno, Período noturno
43	27	Feminino	Especialização/aperfei çoamento	Cam pinas /SP	2 a 5 anos	2 a 5 anos	Assistência Hospitalar	Pública	Período diurno
44	31	Feminino	Especialização/aperfei çoamento	Campinas - SP	6 a 10 anos	6 a 10 anos	Assistência Hospitalar	Pública	Período diurno

Você tem dupla jornada de trabalho?	Jornada diária (em horas)	O quão satisfeito(a) você está com seu trabalho?	Como você se sente no seu ambiente de trabalho? Se necessário, assinale mais de uma opção.
Não	8 horas	Satisfeito(a)	Mais Feliz/Animado(a)/Bem-humorado(a) do que Triste/Desanimado(a)/Mal-humorado(a), Mais Tenso(a)/Estressado(a) do que Tranquilo(a)/Satisfeito(a)
Não	6 horas ou menos	Satisfeito(a)	Mais Feliz/Animado(a)/Bem-humorado(a) do que Triste/Desanimado(a)/Mal-humorado(a)
Não	12 horas (Escala 12x36)	Pouco satisfeito(a)	Mais Tranquilo(a)/Satisfeito(a) do que Tenso(a)/Estressado(a)
Não	8 horas	Satisfeito(a)	Mais Tranquilo(a)/Satisfeito(a) do que Tenso(a)/Estressado(a)
Não	8 horas	Satisfeito(a)	Mais Tenso(a)/Estressado(a) do que Tranquilo(a)/Satisfeito(a)
Não	8 horas	Satisfeito(a)	Mais Feliz/Animado(a)/Bem-humorado(a) do que Triste/Desanimado(a)/Mal-humorado(a)
Não	Outro ou não se aplica	Satisfeito(a)	Mais Feliz/Animado(a)/Bem-humorado(a) do que Triste/Desanimado(a)/Mal-humorado(a), Mais Tenso(a)/Estressado(a) do que Tranquilo(a)/Satisfeito(a)
Não	8 horas	Satisfeito(a)	Mais Feliz/Animado(a)/Bem-humorado(a) do que Triste/Desanimado(a)/Mal-humorado(a)
Não	8 horas	Satisfeito(a)	Mais Tenso(a)/Estressado(a) do que Tranquilo(a)/Satisfeito(a)
Sim	8 horas	Satisfeito(a)	Mais Feliz/Animado(a)/Bem-humorado(a) do que Triste/Desanimado(a)/Mal-humorado(a), Mais Tranquilo(a)/Satisfeito(a) do que Tenso(a)/Estressado(a)
Não	8 horas	Muito satisfeito(a)	Mais Feliz/Animado(a)/Bem-humorado(a) do que Triste/Desanimado(a)/Mal-humorado(a), Mais Tranquilo(a)/Satisfeito(a) do que Tenso(a)/Estressado(a)
Não	12 horas (Escala 12x36)	Satisfeito(a)	Mais Tenso(a)/Estressado(a) do que Tranquilo(a)/Satisfeito(a)
Não	8 horas	Satisfeito(a)	Mais Tranquilo(a)/Satisfeito(a) do que Tenso(a)/Estressado(a)
Sim	Outro ou não se aplica	Satisfeito(a)	Mais Feliz/Animado(a)/Bem-humorado(a) do que Triste/Desanimado(a)/Mal-humorado(a), Mais Tranquilo(a)/Satisfeito(a) do que Tenso(a)/Estressado(a)

Não	12 horas (Escala 12x36)	Satisfeito(a)	Mais Feliz/Animado(a)/Bem-humorado(a) do que Triste/Desanimado(a)/Mal-humorado(a)
Não	12 horas (Escala 12x36)	Satisfeito(a)	Mais Tranquilo(a)/Satisfeito(a) do que Tenso(a)/Estressado(a)
Não	6 horas ou menos	Satisfeito(a)	Mais Feliz/Animado(a)/Bem-humorado(a) do que Triste/Desanimado(a)/Mal-humorado(a), Mais Tranquilo(a)/Satisfeito(a) do que Tenso(a)/Estressado(a)
Não	Outro ou não se aplica	Muito satisfeito(a)	Mais Feliz/Animado(a)/Bem-humorado(a) do que Triste/Desanimado(a)/Mal-humorado(a)
Não	8 horas	Muito satisfeito(a)	Mais Feliz/Animado(a)/Bem-humorado(a) do que Triste/Desanimado(a)/Mal-humorado(a)
Não	8 horas	Satisfeito(a)	Mais Tranquilo(a)/Satisfeito(a) do que Tenso(a)/Estressado(a)
Sim	Outro ou não se aplica	Satisfeito(a)	Mais Tenso(a)/Estressado(a) do que Tranquilo(a)/Satisfeito(a)
Sim	8 horas	Satisfeito(a)	Mais Tranquilo(a)/Satisfeito(a) do que Tenso(a)/Estressado(a)
Sim	8 horas	Satisfeito(a)	Mais Feliz/Animado(a)/Bem-humorado(a) do que Triste/Desanimado(a)/Mal-humorado(a)
Sim	Outro ou não se aplica	Pouco satisfeito(a)	Mais Triste/Desanimado(a)/Mal-humorado(a) do que Feliz/Animado(a)/Bem-humorado(a), Mais Tenso(a)/Estressado(a) do que Tranquilo(a)/Satisfeito(a)
Não	8 horas	Muito satisfeito(a)	Mais Feliz/Animado(a)/Bem-humorado(a) do que Triste/Desanimado(a)/Mal-humorado(a)
Não	12 horas (Escala 12x36)	Satisfeito(a)	Mais Triste/Desanimado(a)/Mal-humorado(a) do que Feliz/Animado(a)/Bem-humorado(a)
Não se aplica	Outro ou não se aplica	Muito satisfeito(a)	Mais Tranquilo(a)/Satisfeito(a) do que Tenso(a)/Estressado(a)
Não	8 horas	Satisfeito(a)	Mais Tranquilo(a)/Satisfeito(a) do que Tenso(a)/Estressado(a)
Não	6 horas ou menos	Satisfeito(a)	Mais Tranquilo(a)/Satisfeito(a) do que Tenso(a)/Estressado(a)
Não	8 horas	Satisfeito(a)	Mais Tranquilo(a)/Satisfeito(a) do que Tenso(a)/Estressado(a)
Não	8 horas	Muito satisfeito(a)	Mais Feliz/Animado(a)/Bem-humorado(a) do que Triste/Desanimado(a)/Mal-humorado(a)

Não	8 horas	Satisfeito(a)	Mais Feliz/Animado(a)/Bem-humorado(a) do que Triste/Desanimado(a)/Mal-humorado(a)
Sim	12 horas (Escala 12x36)	Satisfeito(a)	Mais Tenso(a)/Estressado(a) do que Tranquilo(a)/Satisfeito(a)
Não	8 horas	Satisfeito(a)	Mais Feliz/Animado(a)/Bem-humorado(a) do que Triste/Desanimado(a)/Mal-humorado(a), Mais Tenso(a)/Estressado(a) do que Tranquilo(a)/Satisfeito(a)
Não	8 horas	Satisfeito(a)	Mais Tranquilo(a)/Satisfeito(a) do que Tenso(a)/Estressado(a)
Não	8 horas	Muito satisfeito(a)	Mais Feliz/Animado(a)/Bem-humorado(a) do que Triste/Desanimado(a)/Mal-humorado(a), Mais Tranquilo(a)/Satisfeito(a) do que Tenso(a)/Estressado(a)
Não se aplica	Outro ou não se aplica	Muito satisfeito(a)	Mais Tranquilo(a)/Satisfeito(a) do que Tenso(a)/Estressado(a)
Sim	Outro ou não se aplica	Satisfeito(a)	Mais Tranquilo(a)/Satisfeito(a) do que Tenso(a)/Estressado(a)
Não	6 horas ou menos	Satisfeito(a)	Mais Feliz/Animado(a)/Bem-humorado(a) do que Triste/Desanimado(a)/Mal-humorado(a), Mais Tranquilo(a)/Satisfeito(a) do que Tenso(a)/Estressado(a)
Não	6 horas ou menos	Muito satisfeito(a)	Mais Tranquilo(a)/Satisfeito(a) do que Tenso(a)/Estressado(a)
Não	12 horas (Escala 12x36)	Satisfeito(a)	Mais Triste/Desanimado(a)/Mal-humorado(a) do que Feliz/Animado(a)/Bem-humorado(a), Mais Tranquilo(a)/Satisfeito(a) do que Tenso(a)/Estressado(a)
Sim	Outro ou não se aplica	Satisfeito(a)	Mais Tranquilo(a)/Satisfeito(a) do que Tenso(a)/Estressado(a)
Não	6 horas ou menos	Satisfeito(a)	Mais Feliz/Animado(a)/Bem-humorado(a) do que Triste/Desanimado(a)/Mal-humorado(a), Mais Tranquilo(a)/Satisfeito(a) do que Tenso(a)/Estressado(a)
Não	6 horas ou menos	Satisfeito(a)	Mais Triste/Desanimado(a)/Mal-humorado(a) do que Feliz/Animado(a)/Bem-humorado(a), Mais Tenso(a)/Estressado(a) do que Tranquilo(a)/Satisfeito(a)

ID	Quantidade de itens assinalados na Seção A	Seção A: 42 respostas; 29 itens totais	Quantas vezes foram citados
1	12	Participação na visita clínica multiprofissional diária à beira leito.	27 (64,3%)
2	13	Deambulação 3 vezes ao dia ou de acordo com o plano de cuidados de enfermagem, se as condições clínicas permitirem.	14 (33,3%)
3	4	Avaliação da eficácia da medicação.	19 (45,2%)
4	18	Mudança de decúbito da criança a cada 2 horas ou conforme prescrito.	12 (28,6%)
5	9	Cuidados bucais.	22 (52,4%)
6	5	Envolvimento dos pais nos cuidados com a criança.	20 (47,6%)
7	5	Educação do paciente e família.	18 (42,9%)
8	14	Discussão com a criança e sua família sobre planos de alta e cuidados no domicílio.	21 (50%)
9	20	Promoção do desenvolvimento neuroevolutivo, de acordo com a idade e condições clínicas da criança (por exemplo, cuidados neonatais, desenvolvimento cognitivo e relacional da criança ou do adolescente).	20
10	4	Avaliação da dor e intervenções farmacológicas ou não farmacológicas, de acordo com protocolos.	(47,6%)
11	3	Solicitações de medicamentos atendidas dentro de 15 minutos.	16
12	11	Documentação completa com todos os dados necessários.	(38,1%)
13	6	Comunicação de todas as informações relevantes na passagem de plantão ou transferência.	22
14	19	Satisfação das necessidades alimentares, de acordo com as condições clínicas da criança (por exemplo, incentivo a alimentação oral e/ou nutrição do recém-nascido assim que solicitado; incentivo a alimentação apropriada, de acordo com a preferência pessoal).	(52,4%) 20 (47,6%) 16 (38,1%)
15	24	Administração de medicamentos 30 minutos antes ou depois do horário programado (por exemplo, horário programado às 20h, administração entre 19h30 e 20h30).	19 (45,2%) 17 (40,5%)
16	10	Auxílio a criança nas necessidades de eliminação dentro de 5 minutos após a solicitação (por exemplo, ir com a criança ao banheiro ou fornecer os dispositivos apropriados se estiver restrita no leito).	15 (35,7%)
17	0	Resposta à luz de chamada, à solicitação de intervenção ou alarme é iniciada dentro de 5 minutos (por exemplo, monitores, bombas de infusão, aparelhos de ventilação mecânica).	16 (38,1%)
18	11	Apoio emocional à criança e/ou família.	18 (42,9%)
19	14	Coleta de exames laboratoriais realizados conforme prescrito.	8 (19%)
20	29	Higiene corporal e cuidados com a pele.	9 (21,4%)
21	23	Avaliação do local de inserção do cateter intravenoso central e do cateter intravenoso periférico segundo protocolos.	11 (26,2%)
22	13	Cuidados com o local de inserção do cateter intravenoso central e do cateter intravenoso periférico segundo protocolos.	13 (31%)
23	2	Adoção das precauções necessárias para o controle de infecções conforme protocolos (uso de EPIs, desinfecção de dispositivos, isolamento, correto descarte de resíduos).	12 (28,6%)
24	14	Monitoramento dos ganhos e perdas de sólidos e líquidos.	11 (26,2%)
25	29	Avaliação dos sinais vitais de acordo com o plano de cuidados de enfermagem.	9 (21,4%)

9	Reavaliações direcionadas sobre a condição da criança para avaliar melhorias ou agravos durante o plantão.	17 (40,5%)
4	Higienização das mãos.	13 (31%)
8	Avaliação das atividades atribuídas aos cuidadores.	22 (52,4%)
3	Verificação de segurança dos equipamentos e limpeza concorrente do mobiliário realizadas uma vez por plantão ou segundo protocolo (por exemplo, cama, mesa de cabeceira, dispositivos).	29 (69%)
6		
9		
15		
6		
19		
6		
28		
0		
7		
4		
4		
10		
9		
10		
18		

ID	Quant. De itens assinalados Recursos laborais	Quant. De itens assinalados Comunicação	Quant. De itens assinalados Recursos	SOMA	M ÉDIA
	3		3		
1	5	4	2	10	3.33333333
2	4	4	2	11	3
3	1	2	1	8	3.66666666 7
4	5	2	2	4	2.66666666 7
5	3	4	3	11	
6	3	4	3	10	1.33333333
7	6	4	4	10	
8		7		17	3
9	4	5	3	12	3.66666666 4
10	2	3	1	6	7 2
11	1	1	0	2	3.33333333 / 0.666666667
12	6	7	4	17	5.33333333 / 5.666666667 3 5.66666666 7
1	3	3	1	7	2.333333333
3	4	5	3	12	4
1	5	5	2	12	4
4	6	0	1	7	2.333333333
1	0	0	0	0	0
5	3	3	1	7	2.333333333
1	5	5	3	13	4.333333333
6	6	7	4	17	5.666666667
1	6	7	3	16	5.333333333
	6	6	2	14	4.666666667
7	3	4	2	9	3
	5	6	1	12	4

1

8

1

2	6	7	4	17	5.666666667
5	5	5	4	14	4.666666667
2	2	4	2	8	2.666666667
6	5	3	2	10	3.333333333
2	2	0	1	3	1
7	2	2	1	5	1.666666667
2	4	3	0	7	2.333333333
8	4	3	2	9	3
2	3	2	3	8	2.666666667
9	3	6	3	12	4
3	4	7	4	15	5
0	5	7	4	16	5.333333333
3	3	7	4	14	4.666666667
1	6	7	1	14	4.666666667
3	1	6	0	7	2.333333333
2	2	2	2	6	2
3	4	5	3	12	4
3	4	7	4	15	5
3	4	3	1	8	2.666666667
4	3	4	2	9	3

3
5
3
6
3
7
3
8
3
9
4
0
4
1
4
2
4
3
4
4

Seção B: Recursos laborais: 43 respostas; 6 itens totais	Quantas vezes foram citados
Desequilíbrio nas atribuições com pacientes.	20 (46,5%) 33 (76,7%)
Número inadequado deenfermeiros.	22 (51,2%)
Situação de urgência do paciente (por exemplo, piora da condição do paciente).	
	26 (60,5%)
Aumento inesperado do número e/ou gravidade dos pacientes na unidade.	34 (79,1%)
Número inadequado de técnicos/auxiliares de enfermagem.	31 (72,1%)
Interrupções frequentes.	

Comunicação: 41 respostas; 7 itens totais	Quantas vezes foram citados
	27 (65,9%) 33 (80,5%)
	30 (73,2%)
Tensão ou falhas na comunicação com a equipe médica.	
Falta de colaboração entre membros da equipe (por exemplo, enfermeiros, técnicos/auxiliares de enfermagem e médicos).	
Tensão ou falhas na comunicação na equipe de enfermagem.	
Tensão ou falhas na comunicação com outros serviços ou departamentos (por exemplo, banco de sangue, serviço de radiologia, farmácia, etc).	27
Técnico/auxiliar de enfermagem não comunicou que o cuidado à criança não foi realizado.	(65,9%)
Inadequada passagem de plantão entre turnos ou na transferência entre unidades.	20
Outros serviços ou setores não prestaram os cuidados necessários (por exemplo, laboratório de análises, farmácia hospitalar).	(48,8%)
	28

Recursos materiais: 40 respostas; 4 itens totais	Quantas vezes foram citados
	(68,3%)
Materiais/equipamentos não disponíveis quando necessários (por exemplo, bombas de infusão, instrumentais cirúrgicos).	28 (70%)
	23
Materiais/equipamentos não funcionam corretamente quando necessário.	29 (72,5%)
Medicamentos não disponíveis quando necessários.	[illegible]
Falta de familiaridade com equipamento/procedimento/norma.	15 (37,5%)
