# Supplementary material for: Translation and cross-cultural adaptation of the MISSCARE Survey-Ped into Brazilian Portuguese
Source: Rev Bras Enferm. 2024 Jul 19;77(2):e20230060. doi: 10.1590/0034-7167-2023-0060 (PMC11259437; doi:10.1590/0034-7167-2023-0060)
Supplement: 0034-7167-reben-77-02-e20230060-suppl02 [file 0034-7167-reben-77-02-e20230060-suppl02.pdf]

ID	Idade	Formação acadêmica	Experiência profissional	Atuação profissional
1	38	Doutorado	Enfermeira assistencial em unidade de internação pediátrica. Professor titular de graduação em enfermagem. Coordenador de curso de graduação em enfermagem.	Ensino, pesquisa, assistência e gestão
2	41	Doutorado	17 anos na pediatria (3 anos como enfermeiro assistencial e 15 como docente)	Ensino, pesquisa, assistência e gestão
3	33	Doutorado	Enfermeiro assistencial em Unidade de Terapia Intensiva Pediátrica e Docente do curso de Graduação em Enfermagem	Ensino e pesquisa
4	39	Pós-doutorado	UTI Neonatal (enfermeira assistencial); Educação Corporativa; Docência	Ensino e pesquisa
5	41	Doutorado	Enfermeira especialista em terapia intensiva e com atuação na área. Docente do ensino superior em Universidade Pública Federal.	Ensino e pesquisa

ID	es_a0	ei_a0	ee_a0	ec_a0	s_a0	es_a1	ei_a1	ee_a1	ec_a1
1+A:IP	1			1		-1	0	0	0
2	1	1	0	0	suprimir a palavra "atividades de", acho que cuidados de enfermagem já explica as atividades	1	1	1	1
3	1	1	1	0	Atividades e cuidados me pareceu repetitivo. Sugestão: Cuidados de enfermagem não realizados	1	1	1	1
4						1	1	1	1
5	1	1	1	1		1	0	0	0
SOMA	4	3	2	2		3	3	3	3

s_a1	es_a2	ei_a2	ee_a2	ec_a2	s_a2	es_a3	ei_a3
Interpreto a versão em inglês como sendo a omissão de uma visita clínica do enfermeiro apenas. Como se enfermeiro não houvesse comparecido ao leito durante todo o seu plantão. A tradução deixa dúvidas quanto a atividade específica que deixou de ser realizada, podendo levar o leitor a diversas interpretações: a não realização foi referente a uma visita clínica? Ou será que o enfermeiro foi ausente na visita com a equipe de enfermagem? Ou será que o enfermeiro não adentrou ao quarto do paciente durante todo o seu plantão? Sugiro: Visita clínica diária do enfermeiro a beira do leito	1	1	1	1		0	0
	1	1	0	1	talvez usar o termo saída do leito, usamos bastante aqui	1	1
	1	1	1	1		1	1
	1	1	1	1		1	1
Dúvida quanto a equivalência semântica e idiomática entre "daily rounds" e "visita clínica", considerando-se que no Brasil são utilizados termos como rondas de enfermagem e rounds de enfermagem, o que pode variar de acordo com a instituição. A "visita clínica" pode ser compreendida como a visita realizada pelo profissional e não aquela realizada no contexto da passagem de caso.	1	1	1	1		1	1
	5	5	4	5		4	4

ee_a3	ec_a3	s_a3	es_a4	ei_a4	ee_a4	ec_a4	s_a4	es_a5	ei_a5	ee_a5	ec_a5
1	1	Sugiro a troca de "medicação" por "medicamentos"	1	1	1	1		1	1	1	1
1	1		1	1	1	1		0	0	0	1
1	1		1	1	1	1		0	0	-1	0
1	1		1	1	1	1		1	1	1	1
1	1		1	1	1	1		1	1	1	1
5	5		5	5	5	5		3	3	2	4

s_a5	es_a6	ei_a6	ee_a6	ec_a6	s_a6	es_a7	ei_a7	ee_a7	ec_a7
	0	0	0	0	A palavra "envolvimento" pode dar uma interpretação ambígua: os pais estão sendo envolvidos pela equipe? Ou os pais estão se envolvendo? Sugiro a troca por "inclusão dos pais nos cuidados com a criança".	1	1	1	1
higiene oral ou cuidados com a boca estaria mais próximo do que usamos na assistência	1	1	1	1		1	1	0	1
Este tópico no que diz respeito a EE na minha opinião deveria ser cuidados bucais ou cuidados com a boca.	1	1	1	1		1	1	1	1
	1	1	1	1		1	1	1	1
	1	1	1	1		1	1	1	1
	4	4	4	4		5	5	4	5

s_a7	es_a8	ei_a8	ee_a8	ec_a8	s_a8	es_a9	ei_a9	ee_a9	ec_a9	s_a9	es_a10	ei_a10	ee_a10	ec_a10	s_a10
	1	1	1	1		1	1	1	1		1	1	1	1	
se inclui-se educação/orientação ficaria perfeito a realidade daqui	1	1	1	1		1	0	0	1	não é frequentemente usado no cotidiano da assistência	1	1	1	1	
	1	1	1	1		1	1	1	1		1	1	1	1	
	1	1	1	1		1	1	1	1		1	1	1	1	
	1	1	1	1		1	1	1	1		1	1	1	1	
	5	5	5	5		5	4	4	5		5	5	5	5	

es_a11	*ei_a11*	*ee_a11*	*ec_a11*	*s_a11*	*es_a12*	*ei_a12*	*ee_a12*	*ec_a12*	*s_a12*	*es_a13*	*ei_a13*	*ee_a13*	*ec_a13*	*s_a13*
1	1	1	1		1	1	1	1	Erro no formulário. Acredito que seja: Documentação completa de todos os dados necessários" - considerei essa tradução para responder a pergunta- está de acordo em minha avaliação.	1	1	1	1	
1	1	1	1		1	1	1	1		1	1	1	1	
1	1	1	1		1	1	1	1		1	1	1	1	
1	1	1	1		-1	1	1	1	trocar "de" por "com" todos os dados	1	1	1	1	
1	1	1	1		1	1	1	1		1	1	1	1	
5	5	5	5		3	5	5	5		5	5	5	5	

es_a14	*ei_a14*	*ee_a14*	*ec_a14*	*s_a14*	*es_a15*	*ei_a15*	*ee_a15*	*ec_a15*	*s_a15*	*es_a16*	*ei_a16*	*ee_a16*	*ec_a16*
1	1	1	1		1	0	0	1	Sugestão: Horário prescrito. Coloquialmente utilizamos no Brasil o termo -"prescrição" e não "programação".	0	0	0	0
1	1	1	1		1	1	1	1		1	1	1	1
1	1	1	1		1	1	1	1		1	1	1	1
1	1	1	1		1	1	1	1		1	1	1	1
1	1	1	1		1	1	1	1		1	0	1	1
5	5	5	5		5	4	4	5		4	3	4	4

s_a16	*es_a17*	*ei_a17*	*ee_a17*	*ec_a17*	*s_a17*	*es_a18*	*ei_a18*	*ee_a18*	*ec_a18*	*s_a18*	*es_a19*	*ei_a19*	*ee_a19*
Sugiro: "restrito no leito" ou "acamado" - no lugar de somente "no leito". Afinal, "no leito" todos estarão, uma vez que estão hospitalizados em um leito.	1	1	1	1		1	1	1	1		1	1	1
	1	0	0	1	resposta a chamados de campainha ou chamados eletrônicos para auxílio	1	1	1	1		1	1	1
	1	1	1	1		1	1	1	1		1	1	1
	1	1	1	1		1	1	1	1		1	1	1
"auxílio à criança..."	1	1	1	1		1	1	1	1		1	1	1
	5	4	4	5		5	5	5	5		5	5	5

ec_a19	s_a19	es_a20	ei_a20	ee_a20	ec_a20	s_a20	es_a21	ei_a21	ee_a21	ec_a21	s_a21	
	1		1	1	1	1		0	1	1	1	Sugestão: "protocolo" no singular. Uma mesma unidade não pode ter dois protocolos distintos nesta situação. Achei estranho estar no plural.
	1		1	1	1	1		1	0	0	1	reescrever a frase para evitar a repetição de palavras com o mesmo sentido
	1		1	1	1	1		1	1	1	1	
	1		1	1	1	1		1	1	1	1	
	1		1	1	1	1		1	1	1	1	
	5		5	5	5	5		4	4	4	5	

es_a22	ei_a22	ee_a22	ec_a22
0	1	1	1
1	0	0	1
1	1	1	1
1	1	1	1
1	1	1	1
4	4	4	5

s_a22	es_a23	ei_a23	ee_a23	ec_a23	s_a23	es_a24	ei_a24	ee_a24	ec_a24	s_a24
Sugestão: "protocolo" no singular. Uma mesma unidade não pode ter dois protocolos distintos nesta situação. Achei estranho estar no plural.	1	1	1	1		1	1	1	1	
reescrever a frase para evitar a repetição de palavras com o mesmo sentido	1	0	0	1	incluir precauções-padrão - termo bastante utilizado no Brasil	1	1	0	1	usar volume ingerido e volume eliminado
	1	1	1	1		1	1	1	1	
	1	1	1	1		1	1	1	1	
	1	1	1	1		1	1	1	1	
	5	4	4	5		5	5	4	5	

es_a25	ei_a25	ee_a25	ec_a25	s_a25	es_a26	ei_a26	ee_a26	ec_a26	s_a26	es_a27	ei_a27	ee_a27	ec_a27	s_a27	es_a28
1	1	1	1		1	1	1	1		1	1	1	1		0
1	1	1	1		1	0	0	1	Monitorização e reavaliação do paciente conforme deterioração clínica	1	1	1	1		1
1	1	1	1		1	1	1	1		1	1	1	1		1
1	1	1	1		1	1	1	1		1	1	1	1		1
1	1	1	1		1	1	1	1		1	1	1	1		1
5	5	5	5		5	4	4	5		5	5	5	5		4

ei_a28	*ee_a28*	*ec_a28*	*s_a28*	*es_a29*	*ei_a29*	*ee_a29*	*ec_a29*	*s_a29*	*es_b0*	*ei_b0*	*ee_b0*	*ec_b0*	*s_b0*
0	0	0	Sugestão: "atribuídas ao cuidador" - atribuir e realizar são conceitos distintos.	1	1	1	1		1	1	1	1	
0	0	1	aqui incluir familiar ou acompanante	1	1	1	1		1	1	1	1	
1	1	1		1	1	1	1		1	1	1	1	
1	1	1		1	1	1	1		1	1	1	1	
1	1	1		1	0	1	1	Sugestão: retirar a palavra concorrente a fim deixar a frase mais "usual".	1	1	1	1	
3	3	4		5	4	5	5		5	5	5	5	

es_sb1	*ei_sb1*	*ee_sb1*	*ec_sb1*	*s_sb1*	*es_b1*	*ei_b1*	*ee_b1*	*ec_b1*	*s_b1*	*es_b2*	*ei_b2*	*ee_b2*	*ec_b2*
1	0	0	0	Sugestão: "recursos de trabalho"	1	1	1	1		1	1	1	1
1	1	1	1		1	1	0	1	divisão da escala de trabalho dos pacientes	1	1	1	1
1	1	1	1		1	1	1	1		-1	0	0	0
1	1	1	1		1	1	1	0	Atribuições distribuídas de forma desequilibrada	1	1	1	1
1	1	1	1		1	1	1	1		1	1	1	1
5	4	4	4		5	5	4	4		3	4	4	4

s_b2	es_b3	ei_b3	ee_b3	ec_b3	s_b3	es_b4	ei_b4	ee_b4	ec_b4	s_b4	es_b5	ei_b5	ee_b5	ec_b5	s_b5
	1	0	0	0	Sugestão: "Paciente em situação de urgência"	1	1	1	1		1	1	1	1	
	1	1	1	1		1	1	1	1		1	1	1	1	
Sugestão: "número inadequado de enfermeiros", visto que existe a possibilidade de haver profissionais do sexo masculino na equipe.	1	1	1	1		1	1	1	1		1	1	1	1	
	1	1	1	1		1	1	1	1		1	1	1	1	
	1	1	1	1		1	1	1	1		1	1	1	1	
	5	4	4	4		5	5	5	5		5	5	5	5	

es_b6 ei_b6 ee_b6 ec_b6 s_b6 es_				sb2 ee_sb2 ei_sb2 ec_sb2 s_sb2 es_b7				ei_b7	ee_b7	ec_b7	s_b7		es_b8
1	1	1	1	1	1	1	1	0	0	0	0	Sugestão: "falhas de comunicação" no lugar de "barreiras". Sugiro pois esse termo é mais utilizado em nossa cultura. Além disso o termo "communication breakdowns" se traduz como "quebra" ou "falha" e não barreira.	1
1	1	1	1	1	1	1	1	1	0	0	1	limitação ou fragilidade na comunicacao	1
1	1	1	1	1	1	1	1	1	1	1	1		-1
1	1	1	1	1	1	1	1	1	1	1	1		1
1	1	1	1	1	1	1	1	1	1	1	1		1
5	5	5	5	5	5	5	5	4	3	3	4		3

ei_b8	*ee_b8*	*ec_b8*	*s_b8*	*es_b9*	*ei_b9*	*ee_b9*	*ec_b9*	*s_b9*	*es_b10*	*ei_b10*	*ee_b10*	*ec_b10*
1	1	1		0	0	0	0	Sugestão: "falhas de comunicação" no lugar de "barreiras".	0	0	0	0
1	1	1		1	1	0	1	limitações ou fragilidades - idem comentario acima	1	1	0	1
0	0	0	Mesma sugestão. Colocar enfermeiros e técnicos de enfermagem para englobar ambos os gêneros.	1	1	1	1		1	1	1	1
1	1	1		1	1	1	1		1	1	-1	1
1	1	1		1	1	1	1		1	1	1	1
4	4	4		4	4	3	4		4	4	1	4

s_b10	es_b11	ei_b11	ee_b11	ec_b11	s_b11	es_b12	ei_b12	ee_b12	ec_b12	s_b12	es_b13	ei_b13	ee_b13
Sugestão: "falhas de comunicação" no lugar de "barreiras".	1	1	1	1		1	1	1	1		1	1	1
idem acima	1	1	1	1		1	1	1	1		1	1	1
	-1	0	0	0	Sugestão: técnico de enfermagem, que pode ser um profissional do sexo masculino.	1	1	1	1		1	1	1
Trocar a palavra departamentos por setores	1	1	1	1		1	1	1	1		1	1	-1
	1	1	1	1		1	1	1	1		1	1	1
	3	4	4	4		5	5	5	5		5	5	3

ec_b13	*s_b13*	*es_sb3*	*ei_sb3*	*ee_sb3*	*ec_sb3*	*s_sb3*	*es_b14*	*ei_b14*	*ee_b14*	*ec_b14*	*s_b14*	*es_b15*	*ei_b15*
1		1	1	1	1	Erro no formulário: considere para responder a tradução "Recursos materiais"	1	1	1	1		1	1
1		1	1	1	1		1	1	1	1		1	1
1		1	1	1	1		1	1	1	1		1	1
1	Tocar a palavra departamentos por setores	1	1	1	1		1	1	1	1		1	1
1		1	1	1	1		1	1	1	1		1	1
5		5	5	5	5		5	5	5	5		5	5

ee_b15	*ec_b15*	*s_b15*	*es_b16*	*ei_b16*	*ee_b16*	*ec_b16*	*s_b16*	*es_b17*	*ei_b17*	*ee_b17*	*ec_b17*	*s_b17*	
1	1		1	1	1	1		-1	0	0	0		Sugestão: "Familiaridade! no lugar de "treinamento". - O profissional poder ter sido treinado porém não teve oportunidade de muitos contatos com determinado equipamento.
1	1		1	1	1	1		1	1	1	1		
1	1		1	1	1	1		1	1	1	1		
1	1		1	1	1	1		1	1	1	1		
1	1		1	1	1	1		1	1	1	1		
5	5		5	5	5	5		3	4	4	4		

comentários
Fiz comentários considerando a realidade assistencial na qual atuo...
Formulário apresenta clareza nas informações e equivalência entre a versão original e a proposta. Foram incluídas sugestões em alguns itens.

	MISSCARE Survey-Ped - Tradução Síntese (TS)<br>1ª RODADA	ES EI EE EC	IVC do item
	Seção "A" – atividades de cuidados de enfermagem não realizadas	80% 60% 40% 40% 60%	55%
1	Participação na visita clínica diária à beira leito	60% 60% 60%	60%
2	Deambulação 3 vezes ao dia ou de acordo com o plano de cuidados de enfermagem, se as condições clínicas permitirem	100% 100% 80% 100%	95%
3	Avaliação da eficácia da medicação	80% 80%100%100%	90%
4	Mudança de decúbito da criança a cada 2 horas ou conforme prescrito	100% 100% 100% 100%	100%
5	Cuidados orais	60% 60% 40% 80%	60%
6	Envolvimento dos pais nos cuidados com a criança	80% 80% 80% 80%	80%
7	Educação do paciente e família	100% 100% 80% 100%	95%
8	Discussão com a criança e sua família sobre planos de alta e cuidados no domicílio	100% 100% 100% 100%	100%
9	Promoção do desenvolvimento neuroevolutivo, de acordo com a idade e condições clínicas da criança (por exemplo, cuidados neonatais, desenvolvimento cognitivo e relacional da criança ou do adolescente)	100% 80% 80%100%	90%
10	Avaliação da dor e intervenções farmacológicas ou não farmacológicas, de acordo com protocolos	100% 100% 100% 100%	100%
11	Solicitações de medicamentos atendidas dentro de 15 minutos	100% 100% 100% 100%	100%
12	Documentação completa de todos os dados necessários	60% 100% 100% 100%	90%
13	Comunicação de todas as informações relevantes na passagem de plantão ou transferência	100% 100% 100% 100%	100%
14	Satisfação das necessidades alimentares, de acordo com as condições clínicas da criança (por exemplo, incentivo a alimentação oral e/ou nutrição do recém-nascido assim que solicitado; incentivo a alimentação apropriada, de acordo com a preferência pessoal)	100% 100% 100% 100%	100%
15	Administração de medicamentos 30 minutos antes ou depois do horário programado (por exemplo, horário programado às 20h, administração entre 19h30 e 20h30)	100% 80% 80%100%	90%
16	Auxílio a criança nas necessidades de eliminação dentro de 5 minutos após a solicitação (por exemplo, ir com a criança ao banheiro ou fornecer os dispositivos apropriados se estiver no leito)	80% 60% 80% 80%	75%
17	Resposta à luz de chamada, à solicitação de intervenção ou alarme é iniciada dentro de 5 minutos (por exemplo, monitores, bombas de infusão, aparelhos de ventilação mecânica)	100% 80% 80%100%	90%
18	Apoio emocional à criança e/ou família	100% 100% 100% 100%	100%
19	Coleta de exames laboratoriais realizados conforme prescrito	100% 100% 100% 100%	100%

20	Higiene corporal e cuidados com a pele	100%	100%	100%	100%	100%
22	Cuidados com o local de inserção do cateter intravenoso central e do cateter intravenoso periférico segundo protocolos	80%	80%	80%	100%	85%
23	Adoção das precauções necessárias para o controle de infecções conforme protocolos (uso de EPIs, desinfecção de dispositivos, isolamento, correto descarte de resíduos)	100%	80%	80%	100%	90%
24	Monitoramento dos ganhos e perdas de sólidos e líquidos	100%	100%	80%	100%	95%
25	Avaliação dos sinais vitais de acordo com o plano de cuidados de enfermagem	100%	100%	100%	100%	100%
26	Reavaliações direcionadas sobre a condição da criança para avaliar melhorias ou agravos durante o plantão	100%	80%	80%	100%	90%
27	Higienização das mãos	100%	100%	100%	100%	100%
28	Avaliação das atividades realizadas pelo cuidador	80%	60%	60%	80%	70%
29	Verificação de segurança dos equipamentos e limpeza concorrente do mobiliário realizadas uma vez por plantão ou segundo protocolo (por exemplo, cama, mesa de cabeceira, dispositivos)	100%	80%	100%	100%	95%
	Seção "B" – razões para as omissões de cuidados em enfermagem	100%	100%	100%	100%	100%
	Recursos laborais	100%	80%	80%	80%	85%
1	Desequilíbrio nas atribuições com pacientes.	100%	100%	80%	80%	90%
2	Número inadequado de enfermeiras.	60%	80%	80%	80%	75%
3	Situação de urgência do paciente (por exemplo, piora da condição do paciente).	100%	80%	80%	80%	85%
4	Aumento inesperado do número e/ou gravidade dos pacientes na unidade.	100%	100%	100%	100%	100%
5	Número inadequado de técnicos/auxiliares de enfermagem.	100%	100%	100%	100%	100%
6	Interrupções frequentes.	100%	100%	100%	100%	100%
	Comunicação	100%	100%	100%	100%	100%
7	Tensão ou barreiras de comunicação com a equipe médica.	80%	60%	60%	80%	70%
8	Falta de colaboração entre membros da equipe (por exemplo, enfermeiras, técnicas/auxiliares de enfermagem e médicos).	60%	80%	80%	80%	75%
9	Tensão ou barreiras de comunicação na equipe de enfermagem.	80%	80%	60%	80%	75%
10	Tensão ou barreiras de comunicação com outros serviços ou departamentos (por exemplo, banco de sangue, serviço de radiologia, farmácia, etc);	80%	80%	20%	80%	65%

11		Técnica/auxiliar de enfermagem não comunicou que o cuidado à criança não foi realizado.	60%	80%	80%	80%	75%
12		Inadequada passagem de plantão entre turnos ou na transferência entre unidades.	100%	100%	100%	100%	100%
13		Outros serviços ou departamentos não prestaram os cuidados necessários (por exemplo, laboratório de análises, farmácia hospitalar).	100%	100%	60%	100%	90%
	Recursos materiais		100%	100%	100%	100%	100%
14		Materiais/equipamentos não disponíveis quando necessários (por exemplo, bombas de infusão, instrumentais cirúrgicos).	100%	100%	100%	100%	100%
15		Materiais/equipamentos não funcionam corretamente quando necessário.	100%	100%	100%	100%	100%
16		Medicamentos não disponíveis quando necessários.	100%	100%	100%	100%	100%
17		Falta de treinamento com equipamento/procedimento/normas.	60%	80%	80%	80%	75%

IVC do instrumento (1° rodada): 89%

ID	es_a0	ei_a0	ee_a0	ec_a0	s_a0	es_a1	ei_a1	ee_a1	ec_a1	s_a1	es_a5	ei_a5	ee_a5	ec_a5	s_a5	es_a16	ei_a16	ee_a16	ec_a16	s_a16	es_a28
1	1	1	1	1		0	1	1	0	Acredito que a versão em inglês não se refere especificamente a uma visita multidisciplinar. Não fica claro isso.	1	1	1	1		1	1	1	1		1
2	1	1	1	1		1	0	1	0	visita clínica no contexto que se pretende me parece ser o round diário com a equipe multiprofissional, entendo por visita clínica a avaliação diária do enfermeiro separada dos demais	1	1	1	1		1	1	1	1		1
3	1	1	1	1		1	1	1	1		1	1	1	1		1	1	1	1		1
4	1	1	1	1		1	1	1	1		1	1	1	1		1	1	1	1		0

5	1	1	1	1	1	1	1	1	1	1	1	1	1	1	1	1	1
SOMA	5	5	5	5	4	4	5	3	5	5	5	5	5	5	5	5	4

ei_a28	*ee_a28*	*ec_a28*	*s_a28*	*es_b2*	*ei_b2*	*ee_b2*	*ec_b2*	*s_b2*	*es_b7*	*ei_b7*	*ee_b7*	*ec_b7*	*s_b7*	*es_b8*	*ei_b8*	*ee_b8*	*ec_b8*	*s_b8*	*es_b9*	*ei_b9*	*ee_b9*
1	1	1		1	1	1	1		1	1	1	1		1	1	1	1		1	1	1
1	1	1		1	1	1	1		1	1	1	1		1	1	1	1		1	1	1
1	1	1		1	1	1	1		1	1	1	1		1	1	1	1		1	1	1
1	1	0	Realizadas e atribuídas têm significados diferentes. Penso que é preciso considerar o significado no instrumento original.	1	1	1	1		0	1	1	0	Barreiras e falhas têm significados diferentes. Penso que é preciso considerar o significado no instrumento original. Parece-me que falhas é mais pertinente.	1	1	1	1		0	1	1

1	1	1	1	1	1	1	1	1	1	1	1	1	1	1	1	1	1
5	5	4	5	5	5	5	4	5	5	4	5	5	5	5	4	5	5

ec_b9	s_b9	es_b10	ei_b10	ee_b10	ec_b10	s_b10	es_b11	ei_b11	ee_b11	ec_b11	s_b11	es_b17	ei_b17	ee_b17	ec_b17	s_b17	Comentários
1		1	1	1	1		1	1	1	1		1	1	1	1		
1		1	1	1	1		1	1	1	1		1	1	1	1		
1		1	1	1	1		1	1	1	1		1	1	1	1		
0	Barreiras e falhas têm significados diferentes. Penso que é preciso considerar o significado no instrumento original. Parece-me que falhas é mais pertinente.	0	1	1	0	Barreiras e falhas têm significados diferentes. Penso que é preciso considerar o significado no instrumento original. Parece-me que falhas é mais pertinente.	1	1	1	1		1	1	1	1		

1	1	1	1	1	1	1	1	1	1	1	1	1	Estou de acordo com as versões propostas!
4	4	5	5	4	5	5	5	5	5	5	5	5	

	MISSCARE Survey-Ped - Tradução Síntese (TS) 2ª RODADA	ES	EI	EE	EC	IVC do item
	Seção "A" – Cuidados de enfermagem não realizados	100%	100%	100%	100%	100%
1	Participação na visita clínica multiprofissional diária à beira leito	80%	80%	100%	60%	80%
2	Deambulação 3 vezes ao dia ou de acordo com o plano de cuidados de enfermagem, se as condições clínicas permitirem	100%	100%	80%	100%	95%
3	Avaliação da eficácia da medicação	80%	80%	100%	100%	90%
4	Mudança de decúbito da criança a cada 2 horas ou conforme prescrito	100%	100%	100%	100%	100%
5	Cuidados bucais	100%	100%	100%	100%	100%
6	Envolvimento dos pais nos cuidados com a criança	80%	80%	80%	80%	80%
7	Educação do paciente e família	100%	100%	80%	100%	95%
8	Discussão com a criança e sua família sobre planos de alta e cuidados no domicílio	100%	100%	100%	100%	100%
9	Promoção do desenvolvimento neuroevolutivo, de acordo com a idade e condições clínicas da criança (por exemplo, cuidados neonatais, desenvolvimento cognitivo e relacional da criança ou do adolescente)	100%	80%	80%	100%	90%
10	Avaliação da dor e intervenções farmacológicas ou não farmacológicas, de acordo com protocolos	100%	100%	100%	100%	100%
11	Solicitações de medicamentos atendidas dentro de 15 minutos	100%	100%	100%	100%	100%
12	Documentação completa com todos os dados necessários	60%	100%	100%	100%	90%
13	Comunicação de todas as informações relevantes na passagem de plantão ou transferência	100%	100%	100%	100%	100%
14	Satisfação das necessidades alimentares, de acordo com as condições clínicas da criança (por exemplo, incentivo a alimentação oral e/ou nutrição do recém-nascido assim que solicitado; incentivo a alimentação apropriada, de acordo com a preferência pessoal)	100%	100%	100%	100%	100%
15	Administração de medicamentos 30 minutos antes ou depois do horário programado (por exemplo, horário programado às 20h, administração entre 19h30 e 20h30)	100%	80%	80%	100%	90%
16	Auxílio a criança nas necessidades de eliminação dentro de 5 minutos após a solicitação (por exemplo, ir com a criança ao banheiro ou fornecer os dispositivos apropriados se estiver restrita no leito)	100%	100%	100%	100%	100%
17	Resposta à luz de chamada, à solicitação de intervenção ou alarme é iniciada dentro de 5 minutos (por exemplo, monitores, bombas de infusão, aparelhos de ventilação mecânica)	100%	80%	80%	100%	90%
18	Apoio emocional à criança e/ou família	100%	100%	100%	100%	100%
19	Coleta de exames laboratoriais realizados conforme prescrito	100%	100%	100%	100%	100%
20	Higiene corporal e cuidados com a pele	100%	100%	100%	100%	100%

21	Avaliação do local de inserção do cateter intravenosos central e do cateter intravenoso periférico segundo protocolos		80%	80%	80%	100%	85%
22	Cuidados com o local de inserção do cateter intravenoso central e do cateter intravenoso periférico segundo protocolos		80%	80%	80%	100%	85%
23	Adoção das precauções necessárias para o controle de infecções conforme protocolos (uso de EPIs, desinfecção de dispositivos, isolamento, correto descarte de resíduos)		100%	80%	80%	100%	90%
24	Monitoramento dos ganhos e perdas de sólidos e líquidos		100%	100%	80%	100%	95%
25	Avaliação dos sinais vitais de acordo com o plano de cuidados de enfermagem		100%	100%	100%	100%	100%
26	Reavaliações direcionadas sobre a condição da criança para avaliar melhorias ou agravos durante o plantão		100%	80%	80%	100%	90%
27	Higienização das mãos		100%	100%	100%	100%	100%
28	Avaliação das atividades atribuídas aos cuidadores		80%	100%	100%	80%	90%
29	Verificação de segurança dos equipamentos e limpeza concorrente do mobiliário realizadas uma vez por plantão ou segundo protocolo (por exemplo, cama, mesa de cabeceira, dispositivos)		100%	80%	100%	100%	95%
	Seção “B” – razões para as omissões de cuidados em enfermagem		100%	100%	100%	100%	100%
	Recursos laborais		100%	80%	80%	80%	85%
		Desequilíbrio nas atribuições com pacientes.	100%	100%	80%	80%	90%
		Número inadequado de enfermeiros.	100%	100%	100%	100%	100%
		Situação de urgência do paciente (por exemplo, piora da condição do paciente).	100%	80%	80%	80%	85%
		Aumento inesperado do número e/ou gravidade dos pacientes na unidade.	100%	100%	100%	100%	100%
		Número inadequado de técnicos/auxiliares de enfermagem.	100%	100%	100%	100%	100%
		Interrupções frequentes.	100%	100%	100%	100%	100%
	Comunicação		100%	100%	100%	100%	100%
		Tensão ou falhas na comunicação com a equipe médica.	80%	100%	100%	80%	90%
		Falta de colaboração entre membros da equipe (por exemplo, enfermeiros, técnicas/auxiliares de enfermagem e médicos).	100%	100%	100%	100%	100%
		Tensão ou falhas na comunicação na equipe de enfermagem.	80%	100%	100%	80%	90%
		Tensão ou falhas na comunicação com outros serviços ou departamentos (por exemplo, banco de sangue, serviço de radiologia, farmácia, etc);	80%	100%	100%	80%	90%
		Técnico/auxiliar de enfermagem não comunicou que o cuidado à criança não foi realizado.	100%	100%	100%	100%	100%

	Inadequada passagem de plantão entre turnos ou na transferência entre unidades.	100%	100%	100%	100%		100%
	Outros serviços ou setores não prestaram os cuidados necessários (por exemplo, laboratório de análises, farmácia hospitalar).	100%	100%	60%	100%		90%
Recursos materiais		100%	100%	100%	100%		100%
	Materiais/equipamentos não disponíveis quando necessários (por exemplo, bombas de infusão, instrumentais cirúrgicos).	100%	100%	100%	100%		100%
	Materiais/equipamentos não funcionam corretamente quando necessário.	100%	100%	100%	100%		100%
	Medicamentos não disponíveis quando necessários.	100%	100%	100%	100%		100%
	Falta de familiaridade com equipamento/procedimento/norma.	100%	100%	100%	100%		100%
						IVC do instrumento (2° rodada): 95%	

Itens	Validados na 1ª rodada	Validados na 2ª rodada
a0	Não validado	Validado
a1	Não validado	Validado
a2	Validado	Validado
a3	Validado	Validado
a4	Validado	Validado
a5	Não validado	Validado
a6	Validado	Validado
a7	Validado	Validado
a8	Validado	Validado
a9	Validado	Validado
a10	Validado	Validado
a11	Validado	Validado
a12	Validado	Validado
a13	Validado	Validado
a14	Validado	Validado
a15	Validado	Validado
a16	Não validado	Validado
a17	Validado	Validado
a18	Validado	Validado
a19	Validado	Validado
a20	Validado	Validado
a21	Validado	Validado
a22	Validado	Validado
a23	Validado	Validado
a24	Validado	Validado
a25	Validado	Validado
a26	Validado	Validado
a27	Validado	Validado
a28	Não validado	Validado
a29	Validado	Validado
b0	Validado	Validado
sb1	Validado	Validado

b1	Validado	Validado
b2	Não validado	Validado
b3	Validado	Validado
b4	Validado	Validado
b5	Validado	Validado
b6	Validado	Validado
sb2	Validado	Validado
b7	Não validado	Validado
b8	Não validado	Validado
b9	Não validado	Validado
b10	Não validado	Validado
b11	Não validado	Validado
b12	Validado	Validado
b13	Validado	Validado
sb3	Validado	Validado
b14	Validado	Validado
b15	Validado	Validado
b16	Validado	Validado
b17	Não validado	Validado
