# Supplementary material for: Translation and cross-cultural adaptation of the MISSCARE Survey-Ped into Brazilian Portuguese
Source: Rev Bras Enferm. 2024 Jul 19;77(2):e20230060. doi: 10.1590/0034-7167-2023-0060 (PMC11259437; doi:10.1590/0034-7167-2023-0060)
Supplement: 0034-7167-reben-77-02-e20230060-suppl03 [file 0034-7167-reben-77-02-e20230060-suppl03.pdf]

Metodologia: Para verificar a consistência e validade do questionário respondido por profissionais de enfermagem, utilizou-se nas perguntas de escala likert o ivc, rvc, alpha de cronbach e o kappa fleiss.

Verificou-se por meio da correlação do total de itens assinalados na sessão B com os totais assinalados nos tópicos dessa sessão para saber a força da correlação existente entre o total feito na parte B com os totais assinalados nos tópicos pertencentes a essa sessão. Calculou-se a correlação entre o total de itens assinalados na sessão A com o total de itens assinalados na sessão B para verificar a correlação entre o total de itens assinalados na parte A com o total assinalado na parte B. A correlação entre o total de itens da parte A também foi calculado para cada total atingido em cada tópico da parte B. Modelo de regressão simples foi ajustado entre o total de itens assinalados da parte A com o total de itens assinalados na parte B para verificar se o aumento ou diminuição no total de itens da parte B aumentavam ou diminuíam a quantidade de itens assinalados em A.

Para entender que tópicos da parte B contribuíam para o aumento no total de ocorrências da parte A, ajustou-se um modelo linear generalizado quasi poisson considerando como resposta o total da parte A e como covariáveis os totais de cada tópico da parte B.

Programa usado : R versão 4.2.1

Pacotes do R: nortest, hnp

Tabela 1: Matriz de correlações spearman

	Soma (Parte A)	Soma (Parte B)	Soma (laborais)	Soma (comunicação)	Soma (materiais)
Soma(Parte A)	1(<0,001)	0,558(<0,001)	0,602(<0,001)	0,457(0,002)	0,376(0,012)
Soma(Parte B)	0,558(<0,001)	1(<0,001)	0,775(<0,001)	0,917(<0,001)	0,818(<0,001)
Soma(laborais)	0,602(<0,001)	0,775(<0,001)	1(<0,001)	0,576(<0,001)	0,449(<0,001)
Soma(comunicação)	0,457(0,002)	0,917(<0,001)	0,576(<0,001)	1(<0,001)	0,678(<0,001)
Soma(materiais)	0,376(0,012)	0,818(<0,001)	0,449(<0,001)	0,678(<0,001)	1(<0,001)

Nota-se que a soma dos itens da parte B se correlaciona de forma moderada a alta com os totais dos itens laborais, comunicação e materiais. A soma dos itens da parte A se correlaciona moderadamente com a soma dos itens da parte B. Assim, a quantidade de intercorrências estão correlacionadas com a quantidade de motivos elencados na parte B.

Ajustando-se o modelo de regressão simples com soma dos itens da parte A(resposta) e com soma dos itens da parte B(variável explicativa):

Tabela 2: Estimativas do modelo de regressão simples

			IC 95%		
	Estimativa	Desvio padrão	LI	LS	Pr(>|t|)

somaB	1,06591	0,08575	0,8929716	1,238844	<0,001

Assim, a cada aumento de uma unidade na quantidade de motivos elencados na parte B, há um aumento médio de 1,066 na quantidade de intercorrências. A variabilidade explicada do modelo é de 78,23%. Os resíduos seguem normalidade pelo teste de Shapiro Francia(p-valor=0,437)

Ajustando-se um modelo linear generalizado quasi poisson, considerando-se como resposta a soma dos itens da parte A e como covariáveis: soma dos itens laborais e soma dos itens de comunicação

Tabela 3: Estimativas do modelo linear generalizado quasi poisson

			IC 95%		
	Estimativa	Desvio padrão	LI	LS	Pr(>|t|)
Intercepto	1,230	0,293	0,633	1,782	0,000
laborais	0,194	0,073	0,053	0,337	0,011
comunicação	0,082	0,053	-0,019	0,189	0,132

Assim, o aumento dos itens assinalados na parte A se deve a motivos de recursos laborais.
Retirando-se comunicação:

Tabela 4: Estimativas do modelo linear generalizado quasi poisson

			IC 95%		
	Estimativa	Desvio padrão	LI	LS	Pr(>|t|)
Intercepto	1,357	0,289	0,765	1,900	<0,001
laborais	0,255	0,063	0,135	0,381	<0,001

Assim, o aumento de recursos laborais aumenta em média 1,290 cuidados de enf não realizados. Houve relação significante entre apontar cuidados de enf nao realizados por causas laborais (p<0,001)

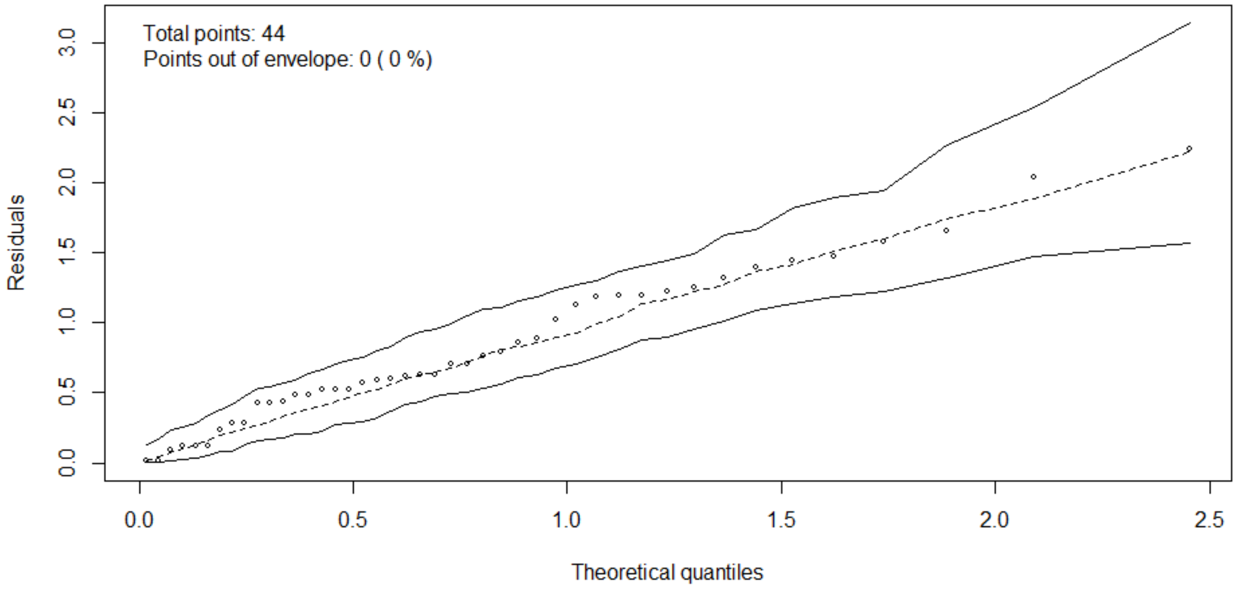

Figura 1: Gráfico halfnormal dos resíduos deviance
Nota-se que todos os pontos estão dentro do envelope indicando adequabilidade do modelo

Calculando-se o Kappa Fleiss para verificar concordância nas respostas dos avaliadores, obtém-se o valor de -0,014, indicando que não há concordância entre os avaliadores para as 3 questões de validação do questionário.

Calculando-se o alpha de cronbach, encontra-se o valor de 0,435, indicando fraca consistência nos itens de validação do questionário.

Resultado da parte B

Tabela 5: Estimativas do IVC e RVC

	O instrumento é claro, inteligível e de fácil compreensão	O instrumento abrange situações relacionadas ao cuidado de enfermagem não realizado e razões para omissões	O instrumento é relevante para a identificação de cuidados de enfermagem não realizados e as razões para omissões
RVC	0,773	0,864	0,955
IVC	88,636%	93,182%	97,727%

Nota-se altos valores de RVC e IVC indicando a consistência nas respostas dos avaliadores para os itens de avaliação do questionário.

Tabela 6: Total de itens assinalados na parte A de acordo com variáveis sociodemográficas

Escolaridade	soma dos itens parte A(Total de intercorrências)				
Níveis	Média(Desvio padrão)	Mediana	Mínimo-Máximo	Primeiro quartil - Terceiro quartil	P-valor
Doutorado	12.786 (9.431)	11.5	0-29	5-18	0.439(ANOVA)*Desconsiderando as categorias com 1 pessoa
Especialização/aperfeiçoamento	11 (6.11)	10.0	4-24	6-14	
Graduação em Enfermagem em andamento	13 (NA)	13.0	13-13	13-13	
Livre-Docência	28 (NA)	28.0	28-28	28-28	
Mestrado	8.2 (5.554)	6.5	0-18	4.5-12.75	
Pós-doutorado	8.4 (6.877)	6.0	3-20	4-9	
Cidade e estado					
Níveis	Média(Desvio padrão)	Mediana	Mínimo-Máximo	Primeiro quartil - Terceiro quartil	
Belo Horizonte - Minas Gerais	9 (NA)	9.0	9-9	9-9	0,072(ANOVA)*Desconsiderando as categorias com 1 pessoa
Campinas/SP	10.5 (5.745)	10.0	4-18	8.5-12	
Campo Grande MS	9 (NA)	9.0	9-9	9-9	
Cuiabá, MT	4 (NA)	4.0	4-4	4-4	
Curitiba- PR	11 (NA)	11.0	11-11	11-11	
Feira de Santana - Bahia	8 (1.414)	8.0	7-9	7.5-8.5	
Florianopolis SC	20 (NA)	20.0	20-20	20-20	
FORTALEZA - CEARÁ	14 (NA)	14.0	14-14	14-14	
imbituba - santa catarina	0 (NA)	0.0	0-0	0-0	
londrina paraná	29 (NA)	29.0	29-29	29-29	
macae- RJ	2 (NA)	2.0	2-2	2-2	
MACEIÓ-AL	14 (NA)	14.0	14-14	14-14	
NITERÓI/RIO DE JANEIRO	6 (NA)	6.0	6-6	6-6	
Ribeirão Preto - SP	21.667 (5.508)	19.0	18-28	18.5-23.5	

Rio de Janeiro/RJ	15.4 (10.407)	13.0	4-29	8-23	
Salvador- BA	5.5 (7.778)	5.5	0-11	2.75-8.25	
São Paulo/SP	9.625 (6.12)	7.5	3-24	5-13.25	
Uberlândia MG	4 (NA)	4.0	4-4	4-4	
Tempo de experiência profissional em anos					
Níveis	Média(Desvio padrão)	Mediana	Mínimo-Máximo	Primeiro quartil - Terceiro quartil	
11 anos ou mais	11,207 (8,152)	10	0-29	4-15	0,346(ANOVA)*Desconsiderando as categorias com 1 pessoa
2 a 5 anos	6,6 (2,881)	6	3-10	5-9	
6 a 10 anos	12,889 (7,849)	11	4-29	7-18	
Não tenho experiência na atuação profissional	13 (NA)	13	13-13	13-13	
Tempo de experiência na área de enfermagem pediátrica					
Níveis	Média(Desvio padrão)	Mediana	Mínimo-Máximo	Primeiro quartil - Terceiro quartil	
11 anos ou mais	11,192 (8,362)	9,5	0-29	4,25-14,75	0,464(Kruskal Wallis)*Desconsiderando as categorias com 1 pessoa
2 a 5 anos	8,25 (4,59)	7,5	3-18	5,75-9,25	
6 a 10 anos	13 (8,093)	11	4-29	7-18	
Não tenho experiência na área	13 (NA)	13	13-13	13-13	
Qual a sua área de atuação? Se necessário, assinale mais de uma opção.					
Níveis	Média(Desvio padrão)	Mediana	Mínimo-Máximo	Primeiro quartil - Terceiro quartil	
Assistência Hospitalar	11.556 (6.287)	10.0	4-24	9-14	0,877(ANOVA)*Desconsid

Assistência Hospitalar, Assistência na Atenção Primária, Docência, Pesquisa	16.5 (17.678)	16.5	4-29	10.25-22.75	erando as categorias com 1 pessoa
Assistência Hospitalar, Assistência na Atenção Primária, Gestão, Docência, Pesquisa	6 (NA)	6.0	6-6	6-6	
Assistência Hospitalar, Docência	11 (NA)	11.0	11-11	11-11	
Assistência Hospitalar, Docência, Pesquisa	11 (6.764)	12.0	4-23	5-14	
Assistência Hospitalar, Gestão	8 (1.414)	8.0	7-9	7.5-8.5	
Assistência Hospitalar, Gestão, Docência, Pesquisa	9 (NA)	9.0	9-9	9-9	
Assistência Hospitalar, Outro	19 (NA)	19.0	19-19	19-19	
Assistência Hospitalar, Pesquisa	10 (3.606)	11.0	6-13	8.5-12	
Assistência na Atenção Primária, Docência	4 (NA)	4.0	4-4	4-4	
Assistência na Atenção Primária, Pesquisa	8 (NA)	8.0	8-8	8-8	
Docência	8.8 (7.981)	9.0	0-19	2-14	
Docência, Pesquisa	14 (11.225)	15.0	3-29	3-20	
Gestão	6 (NA)	6.0	6-6	6-6	
Gestão, Docência, Pesquisa	28 (NA)	28.0	28-28	28-28	

Outro	0 (NA)	0.0	0-0	0-0	
Trabalha em instituição pública ou privada?					
Níveis	Média(Desvio padrão)	Mediana	Mínimo-Máximo	Primeiro quartil - Terceiro quartil	
Ambas	8,5 (7,326)	6,5	2-19	5-10	0,667(Kruskal Wallis)*Desconsiderando as categorias com 1 pessoa
Aposentado/Não empregado	4 (NA)	4	4-4	4-4	
Privada	10,222 (6,534)	9	4-24	6-14	
Pública	11,9 (8,126)	10,5	0-29	5,25-17	
Período do turno de trabalho. Assinale mais de uma opção se necessário.					
Níveis	Média(Desvio padrão)	Mediana	Mínimo-Máximo	Primeiro quartil - Terceiro quartil	
Não se aplica	2 (2,828)	2	0-4	1-3	0,216(Kruskal Wallis)
Período diurno	11,3 (7,962)	9,5	0-29	5,25-14,75	
Período diurno, Período noturno	10,6 (5,941)	9	4-19	7-14	
Período noturno	13 (7,528)	10	4-24	9,5-17	
Você tem dupla jornada de trabalho?					
Níveis	Média(Desvio padrão)	Mediana	Mínimo-Máximo	Primeiro quartil - Terceiro quartil	
Não	11,697 (7,784)	10	0-29	6-15	0,130(Kruskal Wallis)
Não se aplica	2 (2,828)	2	0-4	1-3	
Sim	10,778 (7,032)	9	2-23	6-14	
Jornada diária (em horas)					
Níveis	Média(Desvio padrão)	Mediana	Mínimo-Máximo	Primeiro quartil -	

				Terceiro quartil	
12 horas (Escala 12x36)	10,571 (6,425)	10	4-24	7,5-10,5	
6 horas ou menos	7,429 (6,425)	4	0-18	3,5-11,5	
8 horas	12,81 (8,436)	12	2-29	6-18	
Outro ou não se aplica	10,222 (7,396)	9	0-23	5-14	0,431(Kruskal Wallis)
O quão satisfeito(a) você está com seu trabalho?					
Níveis	Média(Desvio padrão)	Mediana	Mínimo-Máximo	Primeiro quartil - Terceiro quartil	
Muito satisfeito(a)	11,333 (10,654)	9	0-29	4-14	
Pouco satisfeito(a)	9 (7,071)	9	4-14	6,5-11,5	
Satisfeito(a)	11,121 (6,968)	10	0-29	6-15	0,847(Kruskal Wallis)
Como você se sente no seu ambiente de trabalho? Se necessário, assinale mais de uma opção.					
Níveis	Média(Desvio padrão)	Mediana	Mínimo-Máximo	Primeiro quartil - Terceiro quartil	
Mais Feliz/Animado(a)/Bem-humorado(a) do que Triste/Desanimado(a)/Mal-humorado(a)	13,6 (8,058)	13,5	2-29	9,5-14,75	
Mais Feliz/Animado(a)/Bem-humorado(a) do que Triste/Desanimado(a)/Mal-humorado(a),	12 (7)	12	5-19	8,5-15,5	0,236(Kruskal Wallis)*Desconsiderando as categorias com 1 pessoa

Mais Tenso(a)/Estressado(a) do que Tranquilo(a)/Satisfeito(a)					
Mais Feliz/Animado(a)/Bem-humorado(a) do que Triste/Desanimado(a)/Mal-humorado(a), Mais Tranquilo(a)/Satisfeito(a) do que Tenso(a)/Estressado(a)	9,714 (10,21)	4	0-28	3,5-14,5	
Mais Tenso(a)/Estressado(a) do que Tranquilo(a)/Satisfeito(a)	13,8 (7,328)	11	6-23	9-20	
Mais Tranquilo(a)/Satisfeito(a) do que Tenso(a)/Estressado(a)	8,467 (7,16)	6	0-29	4-9,5	
Mais Triste/Desanimado(a)/Mal-humorado(a) do que Feliz/Animado(a)/Bem-humorado(a)	9 (NA)	9	9-9	9-9	
Mais Triste/Desanimado(a)/Mal-humorado(a) do que Feliz/Animado(a)/Bem-humorado(a), Mais Tenso(a)/Estre	16 (2,828)	16	14-18	15-17	

ssado(a) do que Tranquilo(a)/Satisfeito(a)					
Mais Triste/Desanimado(a)/Mal-humorado(a) do que Feliz/Animado(a)/Bem-humorado(a), Mais Tranquilo(a)/Satisfeito(a) do que Tenso(a)/Estressado(a)	10 (NA)	10	10-10	10-10	

Nota-se que o total de intercorrências da parte A não está relacionada com as variáveis sociodemográficas

Tabela 7: Total de itens assinalados na parte B de acordo com variáveis sociodemográficas

	soma dos itens da parte B(motivos das intercorrências)				
Níveis	Média(Desvio padrão)	Mediana	Mínimo-Máximo	Primeiro quartil - Terceiro quartil	P-valor
Doutorado	11.5 (4.57)	11.0	2-17	9-15.5	Dados seguem normalidade nos grupos, mas variâncias desiguais, não significante por games howell nas comparações 2 a 2*Desconsiderando as categorias com 1 pessoa
Especialização/aperfeiçoamento	10.077 (2.362)	10.0	6-14	8-12	
Graduação em Enfermagem em andamento	11 (NA)	11.0	11-11	11-11	
Livre-Docência	16 (NA)	16.0	16-16	16-16	
Mestrado	9.7 (5.813)	10.5	0-17	5.25-14.75	
Pós-doutorado	7.4 (3.209)	7.0	3-12	7-8	
Cidade e estado					
Níveis	Média(Desvio padrão)	Mediana	Mínimo-Máximo	Primeiro quartil - Terceiro quartil	P-valor

Belo Horizonte - Minas Gerais	7 (NA)	7.0	7-7	7-7	0,597(ANOVA)*Desconsiderando as categorias com 1 pessoa
Campinas/SP	8.75 (2.5)	8.5	6-12	7.5-9.75	
Campo Grande MS	14 (NA)	14.0	14-14	14-14	
Cuiabá, MT	8 (NA)	8.0	8-8	8-8	
Curitiba- PR	17 (NA)	17.0	17-17	17-17	
Feira de Santana - Bahia	12.5 (2.121)	12.5	11-14	11.75-13.25	
Florianopolis SC	12 (NA)	12.0	12-12	12-12	
FORTALEZA - CEARÁ	17 (NA)	17.0	17-17	17-17	
imbituba - santa catarina	0 (NA)	0.0	0-0	0-0	
londrina paraná	17 (NA)	17.0	17-17	17-17	
macae- RJ	9 (NA)	9.0	9-9	9-9	
MACEIÓ-AL	12 (NA)	12.0	12-12	12-12	
NITERÓI/RIO DE JANEIRO	7 (NA)	7.0	7-7	7-7	
Ribeirão Preto - SP	10.667 (6.11)	12.0	4-16	8-14	
Rio de Janeiro/RJ	12.6 (4.561)	14.0	6-17	10-16	
Salvador- BA	10.5 (4.95)	10.5	7-14	8.75-12.25	
São Paulo/SP	9.312 (3.842)	10.0	2-15	7-12	
Uberlândia MG	8 (NA)	8.0	8-8	8-8	
Tempo de experiência profissional em anos					
Níveis	Média(Desvio padrão)	Mediana	Mínimo-Máximo	Primeiro quartil - Terceiro quartil	
11 anos ou mais	10.138 (4.206)	10	0-17	7-12	0,365(ANOVA)*Desconsiderando as categorias com 1 pessoa
2 a 5 anos	8.4 (4.336)	8	2-14	8-10	
6 a 10 anos	11.778 (4.658)	13	4-17	9-15	
Não tenho experiência na atuação profissional	11 (NA)	11	11-11	11-11	

Tempo de experiência na área de enfermagem pediátrica					
Níveis	Média(Desvio padrão)	Mediana	Mínimo-Máximo	Primeiro quartil - Terceiro quartil	
11 anos ou mais	10,346 (4,308)	10	0-17	7,25-13,5	0,577(ANOVA)*Desconsiderando as categorias com 1 pessoa
2 a 5 anos	9 (4,504)	9	2-15	7-11,75	
6 a 10 anos	11,222 (4,41)	12	6-17	7-14	
Não tenho experiência na área	11 (NA)	11	11-11	11-11	
Qual a sua área de atuação? Se necessário, assinale mais de uma opção.					
Níveis	Média(Desvio padrão)	Mediana	Mínimo-Máximo	Primeiro quartil - Terceiro quartil	
Assistência Hospitalar	10 (2,693)	10	6-14	8-12	0,599(ANOVA)*Desconsiderando as categorias com 1 pessoa
Assistência Hospitalar, Assistência na Atenção Primária, Docência, Pesquisa	12,5 (6,364)	12,5	8-17	10,25-14,75	
Assistência Hospitalar, Assistência na Atenção Primária, Gestão, Docência, Pesquisa	7 (NA)	7	7-7	7-7	
Assistência Hospitalar, Docência	7 (NA)	7	7-7	7-7	
Assistência Hospitalar, Docência, Pesquisa	10,556 (4,246)	10	4-16	7-14	

Assistência Hospitalar, Gestão	12,5 (2,121)	12,5	11-14	11,75-13,25	
Assistência Hospitalar, Gestão, Docência, Pesquisa	7 (NA)	7	7-7	7-7	
Assistência Hospitalar, Outro	12 (NA)	12	12-12	12-12	
Assistência Hospitalar, Pesquisa	12 (4,583)	11	8-17	9,5-14	
Assistência na Atenção Primária, Docência	8 (NA)	8	8-8	8-8	
Assistência na Atenção Primária, Pesquisa	10 (NA)	10	10-10	10-10	
Docência	13,4 (3,05)	14	9-17	12-15	
Docência, Pesquisa	8,6 (6,269)	9	2-17	3-12	
Gestão	5 (NA)	5	5-5	5-5	
Gestão, Docência, Pesquisa	16 (NA)	16	16-16	16-16	
Outro	0 (NA)	0	0-0	0-0	
Trabalha em instituição pública ou privada?					
Níveis	Média(Desvio padrão)	Mediana	Mínimo-Máximo	Primeiro quartil - Terceiro quartil	
Ambas	10.75 (2.754)	10.5	8-14	8.75-12.5	0,982(ANOVA)*Desconsiderando as categorias com 1 pessoa
Aposentado/Não	8 (NA)	8.0	8-8	8-8	
Privada	10.333 (4.5)	9.0	5-17	7-15	
Pública	10.3 (4.519)	10.5	0-17	7.25-13.75	
Período do turno de trabalho. Assinale mais de uma opção se necessário.					
Níveis	Média(Desvio padrão)	Mediana	Mínimo-Máximo	Primeiro quartil -	

				Terceiro quartil	
Não se aplica	11 (4.243)	11.0	8-14	9.5-12.5	
Período diurno	9.533 (4.447)	9.5	0-17	7-12	
Período diurno, Período noturno	11.8 (3.493)	12.0	6-15	12-14	
Período noturno	12.286 (3.773)	12.0	7-17	10-15	0,378(ANOVA)
Você tem dupla jornada de trabalho?					
Níveis	Média(Desvio padrão)	Mediana	Mínimo-Máximo	Primeiro quartil - Terceiro quartil	
Não	9,848 (4,487)	10	0-17	7-12	
Não se aplica	11 (4,243)	11	8-14	9,5-12,5	
Sim	11,778 (3,42)	12	6-16	9-14	0,482(ANOVA)
Jornada diária (em horas)					
Níveis	Média(Desvio padrão)	Mediana	Mínimo-Máximo	Primeiro quartil - Terceiro quartil	
12 horas (Escala 12x36)	11,143 (3,671)	12	7-17	8-13	
6 horas ou menos	6,286 (3,729)*	7	0-11	4,5-8,5	
8 horas	10,619 (4,444)	10	2-17	7-14	
Outro ou não se aplica	12 (3,122)*	12	7-16	10-14	0,039(ANOVA)
*Grupos significantes pelo teste de Tukey a 5%, p-valor=0,034					
O quão satisfeito(a) você está com seu trabalho?					
Níveis	Média(Desvio padrão)	Mediana	Mínimo-Máximo	Primeiro quartil - Terceiro quartil	
Muito satisfeito(a)	10 (5,148)	8	2-17	7-14	
Pouco satisfeito(a)	10 (2,828)	10	8-12	9-11	
Satisfeito(a)	10,394 (4,19)	10	0-17	8-14	0,967(ANOVA)

Como você se sente no seu ambiente de trabalho? Se necessário, assinale mais de uma opção.					
Níveis	Média(Desvio padrão)	Mediana	Mínimo-Máximo	Primeiro quartil - Terceiro quartil	
Mais Feliz/Animado(a)/Bem-humorado(a) do que Triste/Desanimado(a)/Mal-humorado(a)	11,2 (3,615)	10,5	7-17	9-12,75	
Mais Feliz/Animado(a)/Bem-humorado(a) do que Triste/Desanimado(a)/Mal-humorado(a), Mais Tenso(a)/Estressado(a) do que Tranquilo(a)/Satisfeito(a)	10,667 (1,155)	10	10-12	10-11	
Mais Feliz/Animado(a)/Bem-humorado(a) do que Triste/Desanimado(a)/Mal-humorado(a), Mais Tranquilo(a)/Satisfeito(a) do que Tenso(a)/Estressado(a)	7,286 (5,499)	7	0-16	4-10	0,346(ANOVA)*Desconsiderando as categorias com 1 pessoa
Mais Tenso(a)/Estressado(a) do que	12,8 (3,701)	12	8-17	11-16	

Tranquilo(a)/Satisfeito(a)					
Mais Tranquilo(a)/Satisfeito(a) do que Tenso(a)/Estressado(a)	9,8 (4,617)	8	3-17	6,5-14	
Mais Triste/Desanimado(a)/Mal-humorado(a) do que Feliz/Animado(a)/Bem-humorado(a)	14 (NA)	14	14-14	14-14	
Mais Triste/Desanimado(a)/Mal-humorado(a) do que Feliz/Animado(a)/Bem-humorado(a), Mais Tenso(a)/Estressado(a) do que Tranquilo(a)/Satisfeito(a)	10,5 (2,121)	10,5	9-12	9,75-11,25	
Mais Triste/Desanimado(a)/Mal-humorado(a) do que Feliz/Animado(a)/Bem-humorado(a), Mais Tranquilo(a)/Satisfeito(a) do que Tenso(a)/Estressado(a)	12 (NA)	12	12-12	12-12	

A quantidade média de motivos (Parte B) é diferente entre quem possui jornada de 6 horas ou menos e quem respondeu outro/não se aplica. Quem colocou outro/não se aplica possui mais motivos assinalados na parte B do quem quem possui 6 horas ou menos de jornada

Tabela 8: Total de itens assinalados em recursos laborais de acordo com dados sociodemográficos

	Soma dos itens recursos laborais				
Níveis	Média(Desvio padrão)	Mediana	Mínimo-Máximo	Primeiro quartil - Terceiro quartil	P-valor
Doutorado	4 (1,84)	3,5	1-6	3-6	0,554(ANOVA)*Desconsiderando as categorias com 1 pessoa
Especialização /aperfeiçoamento	4 (1,155)	4	2-6	3-5	
Graduação em Enfermagem em andamento	5 (NA)	5	5-5	5-5	
Livre-Docência	5 (NA)	5	5-5	5-5	
Mestrado	3,4 (2,066)	4	0-6	2-4,75	
Pós-doutorado	3 (1)	3	2-4	2-4	
Cidade e estado					
Níveis	Média(Desvio padrão)	Mediana	Mínimo-Máximo	Primeiro quartil - Terceiro quartil	P-valor
Belo Horizonte - Minas Gerais	4 (NA)	4.0	4-4	4-4	Dados seguem normalidade nos grupos, mas variâncias desiguais, não significante por games howell nas comparações 2 a 2*Desconsiderando as categorias com 1 pessoa
Campinas/SP	3.25 (0.957)	3.5	2-4	2.75-4	
Campo Grande MS	5 (NA)	5.0	5-5	5-5	
Cuiabá, MT	2 (NA)	2.0	2-2	2-2	
Curitiba- PR	6 (NA)	6.0	6-6	6-6	
Feira de Santana - Bahia	5.5 (0.707)	5.5	5-6	5.25-5.75	
Florianopolis SC	4 (NA)	4.0	4-4	4-4	
FORTALEZA - CEARÁ	6 (NA)	6.0	6-6	6-6	
imbituba - santa catarina	0 (NA)	0.0	0-0	0-0	
londrina paraná	6 (NA)	6.0	6-6	6-6	
macae- RJ	3 (NA)	3.0	3-3	3-3	
MACEIÓ-AL	5 (NA)	5.0	5-5	5-5	
NITERÓI/RIO DE JANEIRO	3 (NA)	3.0	3-3	3-3	
Ribeirão Preto - SP	3.333 (2.082)	4.0	1-5	2.5-4.5	
Rio de Janeiro/RJ	5 (1.732)	6.0	2-6	5-6	
Salvador- BA	3 (0)	3.0	3-3	3-3	
São Paulo/SP	3.375 (1.455)	3.0	1-6	2.75-4.25	
Uberlândia MG	4 (NA)	4.0	4-4	4-4	

Tempo de experiência profissional em anos					
Níveis	Média(Desvio padrão)	Mediana	Mínimo-Máximo	Primeiro quartil - Terceiro quartil	
11 anos ou mais	3,724 (1,579)	4	0-6	3-5	0,520(ANOVA)*Desconsiderando as categorias com 1 pessoa
2 a 5 anos	3,2 (1,483)	3	1-5	3-4	
6 a 10 anos	4,222 (1,856)	5	1-6	3-6	
Não tenho experiência na atuação profissional	5 (NA)	5	5-5	5-5	
Tempo de experiência na área de enfermagem pediátrica					
Níveis	Média(Desvio padrão)	Mediana	Mínimo-Máximo	Primeiro quartil - Terceiro quartil	
11 anos ou mais	3.808 (1.625)	4.0	0-6	3-5	0,548(ANOVA)*Desconsiderando as categorias com 1 pessoa
2 a 5 anos	3.25 (1.581)	3.5	1-5	2.5-4.25	
6 a 10 anos	4.111 (1.691)	4.0	2-6	3-6	
Não tenho experiência na área	5 (NA)	5.0	5-5	5-5	
Qual a sua área de atuação? Se necessário, assinale mais de uma opção.					
Níveis	Média(Desvio padrão)	Mediana	Mínimo-Máximo	Primeiro quartil - Terceiro quartil	
Assistência Hospitalar	4,111 (1,269)	4	2-6	3-5	0,709(ANOVA)*Desconsiderando as categorias com 1 pessoa
Assistência Hospitalar, Assistência na Atenção Primária, Docência, Pesquisa	4 (2,828)	4	2-6	3-5	

Assistência Hospitalar, Assistência na Atenção Primária, Gestão, Docência, Pesquisa	3 (NA)	3	3-3	3-3	
Assistência Hospitalar, Docência	3 (NA)	3	3-3	3-3	
Assistência Hospitalar, Docência, Pesquisa	3,444 (1,944)	3	1-6	2-5	
Assistência Hospitalar, Gestão	5,5 (0,707)	5,5	5-6	5,25-5,75	
Assistência Hospitalar, Gestão, Docência, Pesquisa	4 (NA)	4	4-4	4-4	
Assistência Hospitalar, Outro	4 (NA)	4	4-4	4-4	
Assistência Hospitalar, Pesquisa	4,667 (1,528)	5	3-6	4-5,5	
Assistência na Atenção Primária, Docência	4 (NA)	4	4-4	4-4	
Assistência na Atenção Primária, Pesquisa	5 (NA)	5	5-5	5-5	
Docência	3,8 (1,304)	3	3-6	3-4	
Docência, Pesquisa	3,4 (1,949)	4	1-6	2-4	
Gestão	2 (NA)	2	2-2	2-2	
Gestão, Docência, Pesquisa	5 (NA)	5	5-5	5-5	
Outro	0 (NA)	0	0-0	0-0	
Trabalha em instituição pública ou privada?					

Níveis	Média(Desvio padrão)	Mediana	Mínimo-Máximo	Primeiro quartil - Terceiro quartil	
Ambas	4 (1,414)	3,5	3-6	3-4,5	0,975(ANOVA)*Desconsiderando as categorias com 1 pessoa
Aposentado/Não empregado	2 (NA)	2	2-2	2-2	
Privada	3,778 (1,787)	4	1-6	2-5	
Pública	3,833 (1,621)	4	0-6	3-5	
Período do turno de trabalho. Assinale mais de uma opção se necessário.					
Níveis	Média(Desvio padrão)	Mediana	Mínimo-Máximo	Primeiro quartil - Terceiro quartil	
Não se aplica	2,5 (0,707)	2,5	2-3	2,25-2,75	0,050(ANOVA)
Período diurno	3,5 (1,635)*	3	0-6	3-5	
Período diurno, Período noturno	4,2 (1,483)	4	2-6	4-5	
Período noturno	5,143 (0,9)*	5	4-6	4,5-6	
*Diferença de médias pelo teste de Tukey a 10%, p-valor=0,062					
Você tem dupla jornada de trabalho?					
Níveis	Média(Desvio padrão)	Mediana	Mínimo-Máximo	Primeiro quartil - Terceiro quartil	
Não	3,727 (1,645)	4	0-6	3-5	0,314(ANOVA)
Não se aplica	2,5 (0,707)	2,5	2-3	2,25-2,75	
Sim	4,333 (1,5)	4	2-6	3-6	
Jornada diária (em horas)					
Níveis	Média(Desvio padrão)	Mediana	Mínimo-Máximo	Primeiro quartil - Terceiro quartil	
12 horas (Escala 12x36)	4,714 (1,113)*	5	3-6	4-5,5	0,051(ANOVA)
6 horas ou menos	2,429 (1,718)*	2	0-5	1,5-3,5	
8 horas	3,857 (1,59)	4	1-6	3-5	
Outro ou não se aplica	4 (1,414)	4	2-6	3-5	
*Diferença de médias pelo teste de Tukey a 5%, p-valor=0,036					
O quão satisfeito(a)					

você está com seu trabalho?					
Níveis	Média(Desvio padrão)	Mediana	Mínimo-Máximo	Primeiro quartil - Terceiro quartil	
Muito satisfeito(a)	3,444 (1,667)	3	1-6	2-5	0,665(ANOVA)
Pouco satisfeito(a)	4,5 (0,707)	4,5	4-5	4,25-4,75	
Satisfeito(a)	3,848 (1,642)	4	0-6	3-5	
Como você se sente no seu ambiente de trabalho? Se necessário, assinale mais de uma opção.					
Níveis	Média(Desvio padrão)	Mediana	Mínimo-Máximo	Primeiro quartil - Terceiro quartil	
Mais Feliz/Animado(a)/Bem-humorado(a) do que Triste/Desanimado(a)/Mal-humorado(a)	4,4 (1,174)*	4,5	3-6	3,25-5	0,037(Games Howell)*Desconsiderando as categorias com 1 pessoa
Mais Feliz/Animado(a)/Bem-humorado(a) do que Triste/Desanimado(a)/Mal-humorado(a), Mais Tenso(a)/Estressado(a) do que Tranquilo(a)/Satisfeito(a)	3 (0)*	3	3-3	3-3	
Mais Feliz/Animado(a)/Bem-humorado(a) do que Triste/Desanimado(a)/Mal-humorado(a), Mais Tranquilo(a)/S	2,429 (1,902)	2	0-5	1-4	

atisfeito(a) do que Tenso(a)/Estressado(a)					
Mais Tenso(a)/Estressado(a) do que Tranquilo(a)/Satisfeito(a)	4,8 (1,304)	5	3-6	4-6	
Mais Tranquilo(a)/Satisfeito(a) do que Tenso(a)/Estressado(a)	3,733 (1,751)	4	1-6	2-5,5	
Mais Triste/Desanimado(a)/Mal-humorado(a) do que Feliz/Animado(a)/Bem-humorado(a)	5 (NA)	5	5-5	5-5	
Mais Triste/Desanimado(a)/Mal-humorado(a) do que Feliz/Animado(a)/Bem-humorado(a), Mais Tenso(a)/Estressado(a) do que Tranquilo(a)/Satisfeito(a)	4 (1,414)	4	3-5	3,5-4,5	
Mais Triste/Desanimado(a)/Mal-humorado(a) do que Feliz/Animado(a)/Bem-humorado(a), Mais Tranquilo(a)/S	4 (NA)	4	4-4	4-4	

atisfeito(a) do que Tenso(a)/Estressado(a)					
*Diferença nos grupos pelo teste Games Howell					

A quantidade de itens assinalados na parte B é maior em média em quem se sente Mais Feliz/Animado(a)/Bem-humorado(a) do que Triste/Desanimado(a)/Mal-humorado(a) do que quem se sente Mais Feliz/Animado(a)/Bem-humorado(a) do que Triste/Desanimado(a)/Mal-humorado(a), Mais Tenso(a)/Estressado(a) do que Tranquilo(a)/Satisfeito(a)

A quantidade de itens assinalados na parte B é maior em média em quem possui jornada de 12 horas do que quem possui de 6 horas ou menos.

Tabela 9: Total de itens assinalados em comunicação de acordo com variáveis sociodemográficas

	Soma dos itens de comunicação				
Níveis	Média(Desvio padrão)	Mediana	Mínimo-Máximo	Primeiro quartil - Terceiro quartil	P-valor
Doutorado	5.071 (2.018)	6	1-7	3.25-7	0,216(ANOVA)*Desconsiderando as categorias com 1 pessoa
Especialização/aperfeiçoamento	3.769 (1.641)	4	0-6	3-5	
Graduação em Enfermagem em andamento	4 (NA)	4	4-4	4-4	
Livre-Docência	7 (NA)	7	7-7	7-7	
Mestrado	4.2 (2.7)	4	0-7	2-7	
Pós-doutorado	3 (1.871)	3	0-5	3-4	
Cidade e estado					
Níveis	Média(Desvio padrão)	Mediana	Mínimo-Máximo	Primeiro quartil - Terceiro quartil	P-valor
Belo Horizonte - Minas Gerais	3 (NA)	3	3-3	3-3	0,664(ANOVA)*Desconsiderando as categorias com 1 pessoa
Campinas/SP	3,5 (1,291)	3,5	2-5	2,75-4,25	
Campo Grande MS	5 (NA)	5	5-5	5-5	
Cuiabá, MT	4 (NA)	4	4-4	4-4	
Curitiba- PR	7 (NA)	7	7-7	7-7	
Feira de Santana - Bahia	5,5 (2,121)	5,5	4-7	4,75-6,25	

Florianopolis SC	5 (NA)	5	5-5	5-5	
FORTALEZA - CEARÁ	7 (NA)	7	7-7	7-7	
imbituba - santa catarina	0 (NA)	0	0-0	0-0	
londrina paraná	7 (NA)	7	7-7	7-7	
macae- RJ	4 (NA)	4	4-4	4-4	
MACEIÓ-AL	6 (NA)	6	6-6	6-6	
NITERÓI/RIO DE JANEIRO	3 (NA)	3	3-3	3-3	
Ribeirão Preto - SP	4,667 (2,517)	5	2-7	3,5-6	
Rio de Janeiro/RJ	5,2 (2,049)	6	3-7	3-7	
Salvador- BA	5 (2,828)	5	3-7	4-6	
São Paulo/SP	3,75 (2,266)	4	0-7	2-5,25	
Uberlândia MG	2 (NA)	2	2-2	2-2	
Tempo de experiência profissional em anos					
Níveis	Média(Desvio padrão)	Mediana	Mínimo-Máximo	Primeiro quartil - Terceiro quartil	
11 anos ou mais	4,241 (2,214)	4	0-7	3-6	0,188(Kruskal Wallis)*Desconsiderando as categorias com 1 pessoa
2 a 5 anos	3 (1,581)	3	1-5	2-4	
6 a 10 anos	5,111 (1,965)	5	2-7	4-7	
Não tenho experiência na atuação profissional	4 (NA)	4	4-4	4-4	
Tempo de experiência na área de enfermagem pediátrica					
Níveis	Média(Desvio padrão)	Mediana	Mínimo-Máximo	Primeiro quartil - Terceiro quartil	
11 anos ou mais	4,346 (2,279)	4,5	0-7	3-6	
2 a 5 anos	3,5 (1,927)	3,5	1-7	2-4,25	
6 a 10 anos	4,778 (1,922)	5	2-7	3-7	
Não tenho experiência na área 4 (NA)	4		4-4	4-4	0,466(ANOVA)

Qual a sua área de atuação? Se necessário, assinale mais de uma opção.					
Níveis	Média(Desvio padrão)	Mediana	Mínimo-Máximo	Primeiro quartil - Terceiro quartil	
Assistência Hospitalar	3,778 (1,856)	4	0-6	3-5	
Assistência Hospitalar, Assistência na Atenção Primária, Docência, Pesquisa	5,5 (2,121)	5,5	4-7	4,75-6,25	
Assistência Hospitalar, Assistência na Atenção Primária, Gestão, Docência, Pesquisa	3 (NA)	3	3-3	3-3	
Assistência Hospitalar, Docência	3 (NA)	3	3-3	3-3	
Assistência Hospitalar, Docência, Pesquisa	4,889 (1,764)	5	2-7	4-6	
Assistência Hospitalar, Gestão	5,5 (2,121)	5,5	4-7	4,75-6,25	
Assistência Hospitalar, Gestão, Docência, Pesquisa	3 (NA)	3	3-3	3-3	
Assistência Hospitalar, Outro	5 (NA)	5	5-5	5-5	
Assistência Hospitalar, Pesquisa	4,333 (2,517)	4	2-7	3-5,5	0,269(ANOVA)*Desconsiderando as categorias com 1 pessoa
Assistência na Atenção Primária, Docência	2 (NA)	2	2-2	2-2	

Assistência na Atenção Primária, Pesquisa	3 (NA)	3	3-3	3-3	
Docência	6,2 (1,304)	7	4-7	6-7	
Docência, Pesquisa	3,2 (2,864)	3	0-7	1-5	
Gestão	2 (NA)	2	2-2	2-2	
Gestão, Docência, Pesquisa	7 (NA)	7	7-7	7-7	
Outro	0 (NA)	0	0-0	0-0	
Trabalha em instituição pública ou privada?					
Níveis	Média(Desvio padrão)	Mediana	Mínimo-Máximo	Primeiro quartil - Terceiro quartil	
Ambas	4,5 (2,082)	4,5	2-7	3,5-5,5	0,936(ANOVA)*Desconsiderando as categorias com 1 pessoa
Aposentado/Não empregado	4 (NA)	4	4-4	4-4	
Privada	4,444 (2,555)	5	0-7	3-7	
Pública	4,2 (2,091)	4	0-7	3-6	
Período do turno de trabalho. Assinale mais de uma opção se necessário.					
Níveis	Média(Desvio padrão)	Mediana	Mínimo-Máximo	Primeiro quartil - Terceiro quartil	
Não se aplica	5,5 (2,121)	5,5	4-7	4,75-6,25	0,336(ANOVA)
Período diurno	3,933 (2,05)	4	0-7	3-5,75	
Período diurno, Período noturno	5,6 (1,673)	6	3-7	5-7	
Período noturno	4,429 (2,573)	5	0-7	3,5-6	
Você tem dupla jornada de trabalho?					
Níveis	Média(Desvio padrão)	Mediana	Mínimo-Máximo	Primeiro quartil - Terceiro quartil	
Não	3,939(2,135)	4	0-7	3-5	0,195(ANOVA)
Não se aplica	5,5(2,121)	5,5	4-7	4,75-6,25	
Sim	5,222(1,856)	6	2-7	4-7	

Jornada diária (em horas)					
Níveis	Média(Desvio padrão)	Mediana	Mínimo-Máximo	Primeiro quartil - Terceiro quartil	
12 horas (Escala 12x36)	3.714 (2.43)	5	0-7	2-5	
6 horas ou menos	2.714 (2.215)*	3	0-6	1-4	
8 horas	4.429 (1.912)	4	1-7	3-6	
Outro ou não se aplica	5.556 (1.59)*	6	3-7	4-7	0,046(ANOVA)
*Diferença de médias entre quem possui 6 horas ou menos e quem respondeu outro, p-valor=0,035					
O quão satisfeito(a) você está com seu trabalho?					
Níveis	Média(Desvio padrão)	Mediana	Mínimo-Máximo	Primeiro quartil - Terceiro quartil	
Muito satisfeito(a)	4.333 (2.291)	4	1-7	3-7	
Pouco satisfeito(a)	4 (2.828)	4	2-6	3-5	0,952(Kruskal Wallis)
Satisfeito(a)	4.273 (2.111)	4	0-7	3-6	
Como você se sente no seu ambiente de trabalho? Se necessário, assinale mais de uma opção.					
Níveis	Média(Desvio padrão)	Mediana	Mínimo-Máximo	Primeiro quartil - Terceiro quartil	
Mais Feliz/Animado(a)/Bem-humorado(a) do que Triste/Desanimado(a)/Mal-humorado(a)	4.5 (1.509)	4	3-7	3.25-5	0,854(ANOVA)*Desconsiderando as categorias com 1 pessoa

Mais Feliz/Animado(a)/Bem-humorado(a) do que Triste/Desanimado(a)/Mal-humorado(a), Mais Tenso(a)/Estressado(a) do que Tranquilo(a)/Satisfeito(a)	4.667 (1.155)	4	4-6	4-5	
Mais Feliz/Animado(a)/Bem-humorado(a) do que Triste/Desanimado(a)/Mal-humorado(a), Mais Tranquilo(a)/Satisfeito(a) do que Tenso(a)/Estressado(a)	3.571 (2.573)	3	0-7	2-5.5	
Mais Tenso(a)/Estressado(a) do que Tranquilo(a)/Satisfeito(a)	5 (2.121)	5	2-7	4-7	
Mais Tranquilo(a)/Satisfeito(a) do que Tenso(a)/Estressado(a)	3.933 (2.658)	3	0-7	2-7	
Mais Triste/Desanimado(a)/Mal-humorado(a) do que Feliz/Animado(a)/Bem-humorado(a)	5 (NA)	5	5-5	5-5	

Mais Triste/Desanimado(a)/Mal-humorado(a) do que Feliz/Animado(a)/Bem-humorado(a), Mais Tenso(a)/Estressado(a) do que Tranquilo(a)/Satisfeito(a)	5 (1.414)	5	4-6	4.5-5.5	
Mais Triste/Desanimado(a)/Mal-humorado(a) do que Feliz/Animado(a)/Bem-humorado(a), Mais Tranquilo(a)/Satisfeito(a) do que Tenso(a)/Estressado(a)	5 (NA)	5	5-5	5-5	

Em média a quantidade de reclamações da parte B de quem tem jornada outra é maior do que de quem tem de 6 horas ou menos

Tabela 10: Total de itens assinalados em recursos materiais de acordo com variáveis sociodemográficas

	Soma dos itens de recurso material				
Níveis	Média(Desvio padrão)	Mediana	Mínimo-Máximo	Primeiro quartil - Terceiro quartil	P-valor
Doutorado	2.429 (1.399)	2.5	0-4	2-3.75	0,476(ANOVA)*Desconsiderando as categorias com 1 pessoa
Especialização/aperfeiçoamento	2.308 (0.947)	2.0	1-4	2-3	
Graduação em Enfermagem em andamento	2 (NA)	2.0	2-2	2-2	
Livre-Docência	4 (NA)	4.0	4-4	4-4	
Mestrado	2.1 (1.524)	1.5	0-4	1-3.75	
Pós-doutorado	1.4 (1.14)	1.0	0-3	1-2	

Cidade e estado					
Níveis	Média(Desvio padrão)	Mediana	Mínimo-Máximo	Primeiro quartil - Terceiro quartil	P-valor
Belo Horizonte - Minas Gerais	0 (NA)	0.0	0-0	0-0	
Campinas/SP	2 (0.816)	2.0	1-3	1.75-2.25	
Campo Grande MS	4 (NA)	4.0	4-4	4-4	
Cuiabá, MT	2 (NA)	2.0	2-2	2-2	
Curitiba- PR	4 (NA)	4.0	4-4	4-4	
Feira de Santana - Bahia	1.5 (0.707)	1.5	1-2	1.25-1.75	
Florianopolis SC	3 (NA)	3.0	3-3	3-3	
FORTALEZA - CEARÁ	4 (NA)	4.0	4-4	4-4	
imbituba - santa catarina	0 (NA)	0.0	0-0	0-0	
londrina paraná	4 (NA)	4.0	4-4	4-4	
macae- RJ	2 (NA)	2.0	2-2	2-2	
MACEIÓ-AL	1 (NA)	1.0	1-1	1-1	
NITERÓI/RIO DE JANEIRO	1 (NA)	1.0	1-1	1-1	
Ribeirão Preto - SP	2.667 (1.528)	3.0	1-4	2-3.5	
Rio de Janeiro/RJ	2.4 (1.14)	2.0	1-4	2-3	
Salvador- BA	2.5 (2.121)	2.5	1-4	1.75-3.25	
São Paulo/SP	2.188 (1.276)	2.5	0-4	1-3	
Uberlândia MG	2 (NA)	2.0	2-2	2-2	0,924(ANOVA)*Desconsiderando as categorias com 1 pessoa
Tempo de experiência profissional em anos					
Níveis	Média(Desvio padrão)	Mediana	Mínimo-Máximo	Primeiro quartil - Terceiro quartil	
11 anos ou mais	2.172 (1.256)	2	0-4	1-3	0,883(Kruskal Wallis)*Desconsiderando as categorias com 1 pessoa
2 a 5 anos	2.2 (1.643)	3	0-4	1-3	
6 a 10 anos	2.444 (1.333)	2	1-4	1-4	

Não tenho experiência na atuação profissional	2 (NA)	2	2-2	2-2	
Tempo de experiência na área de enfermagem pediátrica					
Níveis	Média(Desvio padrão)	Mediana	Mínimo-Máximo	Primeiro quartil - Terceiro quartil	
11 anos ou mais	2,192 (1,297)	2	0-4	1-3	0,974(Kruskal Wallis)*Desconsiderando as categorias com 1 pessoa
2 a 5 anos	2,25 (1,488)	2,5	0-4	1-3,25	
6 a 10 anos	2,333 (1,225)	2	1-4	1-3	
Não tenho experiência na área	2 (NA)	2	2-2	2-2	
Qual a sua área de atuação? Se necessário, assinale mais de uma opção.					
Níveis	Média(Desvio padrão)	Mediana	Mínimo-Máximo	Primeiro quartil - Terceiro quartil	
Assistência Hospitalar	2,111 (1,054)	2	1-4	1-3	0,339(ANOVA)*Desconsiderando as categorias com 1 pessoa
Assistência Hospitalar, Assistência na Atenção Primária, Docência, Pesquisa	3 (1,414)	3	2-4	2,5-3,5	
Assistência Hospitalar, Assistência na Atenção Primária, Gestão, Docência, Pesquisa	1 (NA)	1	1-1	1-1	
Assistência Hospitalar, Docência	1 (NA)	1	1-1	1-1	

Assistência Hospitalar, Docência, Pesquisa	2,222 (1,302)	3	0-4	1-3	
Assistência Hospitalar, Gestão	1,5 (0,707)	1,5	1-2	1,25-1,75	
Assistência Hospitalar, Gestão, Docência, Pesquisa	0 (NA)	0	0-0	0-0	
Assistência Hospitalar, Outro	3 (NA)	3	3-3	3-3	
Assistência Hospitalar, Pesquisa	3 (1)	3	2-4	2,5-3,5	
Assistência na Atenção Primária, Docência	2 (NA)	2	2-2	2-2	
Assistência na Atenção Primária, Pesquisa	2 (NA)	2	2-2	2-2	
Docência	3,4 (0,894)	4	2-4	3-4	
Docência, Pesquisa	2 (1,581)	2	0-4	1-3	
Gestão	1 (NA)	1	1-1	1-1	
Gestão, Docência, Pesquisa	4 (NA)	4	4-4	4-4	
Outro	0 (NA)	0	0-0	0-0	
Trabalha em instituição pública ou privada?					
Níveis	Média(Desvio padrão)	Mediana	Mínimo-Máximo	Primeiro quartil - Terceiro quartil	
Ambas	2,25 (0,957)	2,5	1-3	1,75-3	0,943(Kruskal Wallis)*Desconsiderando as categorias com 1 pessoa
Aposentado/Não empregado	2 (NA)	2	2-2	2-2	
Privada	2,111 (1,537)	2	0-4	1-4	
Pública	2,267 (1,285)	2	0-4	1-3	
Período do turno de					

trabalho. Assinale mais de uma opção se necessário.					
Níveis	Média(Desvio padrão)	Mediana	Mínimo-Máximo	Primeiro quartil - Terceiro quartil	
Não se aplica	3 (1,414)	3	2-4	2,5-3,5	
Período diurno	2,1 (1,296)	2	0-4	1-3	
Período diurno, Período noturno	2 (1,414)	1	1-4	1-3	
Período noturno	2,714 (1,113)	3	1-4	2-3,5	0,534(Kruskal Wallis)
Você tem dupla jornada de trabalho?					
Níveis	Média(Desvio padrão)	Mediana	Mínimo-Máximo	Primeiro quartil - Terceiro quartil	
Não 2,182 (1,334)	2	0-4		1-3	
Não se aplica 3 (1,414)	3	2-4		2,5-3,5	
Sim 2,222 (1,093)	2	1-4		1-3	0,687(Kruskal Wallis)
Jornada diária (em horas)					
Níveis	Média(Desvio padrão)	Mediana	Mínimo-Máximo	Primeiro quartil - Terceiro quartil	
12 horas (Escala 12x36)	2,714 (1,113)	3	1-4	2-3,5	
6 horas ou menos	1,143 (0,9)	1	0-2	0,5-2	
8 horas	2,333 (1,317)	2	0-4	1-3	
Outro ou não se aplica	2,444 (1,236)	3	1-4	1-3	0,084(ANOVA)
O quão satisfeito(a) você está com seu trabalho?					
Níveis	Média(Desvio padrão)	Mediana	Mínimo-Máximo	Primeiro quartil - Terceiro quartil	
Muito satisfeito(a)	2,222 (1,641)	2	0-4	1-4	
Pouco satisfeito(a)	1,5 (0,707)	1,5	1-2	1,25-1,75	0,669(Kruskal Wallis)

Satisfeito(a)	2,273 (1,206)	2	0-4	1-3	
Como você se sente no seu ambiente de trabalho? Se necessário, assinale mais de uma opção.					
Níveis	Média(Desvio padrão)	Mediana	Mínimo-Máximo	Primeiro quartil - Terceiro quartil	
Mais Feliz/Animado(a)/Bem-humorado(a) do que Triste/Desanimado(a)/Mal-humorado(a)	2,3 (1,252)	2	0-4	2-3	0,169(Kruskal Wallis)*Desconsiderando as categorias com 1 pessoa
Mais Feliz/Animado(a)/Bem-humorado(a) do que Triste/Desanimado(a)/Mal-humorado(a), Mais Tenso(a)/Estressado(a) do que Tranquilo(a)/Satisfeito(a)	3 (0)	3	3-3	3-3	
Mais Feliz/Animado(a)/Bem-humorado(a) do que Triste/Desanimado(a)/Mal-humorado(a), Mais Tranquilo(a)/Satisfeito(a) do que Tenso(a)/Estressado(a)	1,286 (1,604)	1	0-4	0-2	
Mais Tenso(a)/Estressado(a) do que	3 (0,707)	3	2-4	3-3	

Tranquilo(a)/Satisfeito(a)					
Mais Tranquilo(a)/Satisfeito(a) do que Tenso(a)/Estressado(a)	2,133 (1,246)	2	1-4	1-3	
Mais Triste/Desanimado(a)/Mal-humorado(a) do que Feliz/Animado(a)/Bem-humorado(a)	4 (NA)	4	4-4	4-4	
Mais Triste/Desanimado(a)/Mal-humorado(a) do que Feliz/Animado(a)/Bem-humorado(a), Mais Tenso(a)/Estressado(a) do que Tranquilo(a)/Satisfeito(a)	1,5 (0,707)	1,5	1-2	1,25-1,75	
Mais Triste/Desanimado(a)/Mal-humorado(a) do que Feliz/Animado(a)/Bem-humorado(a), Mais Tranquilo(a)/Satisfeito(a) do que Tenso(a)/Estressado(a)	3 (NA)	3	3-3	3-3	

Ajustando-se um modelo de regressão múltipla considerando como resposta total de itens assinalados em laborais e como covariáveis: período do turno de trabalho, jornada diária e sensação ambiente de trabalho, nota-se que nem todas são significativas.
Reajustando-se o modelo obteve-se:

Tabela 11: Estimativas da regressão linear múltipla

			IC 95%		
	Estimativa	Desvio padrão	LI	LS	Pr(>|t|)
Intercepto	3,8	0,2675	3,2589513	4,3410487	<0,001
periodo noturno	1,6	0,7077	0,1685196	3,0314804	0,0294
Mais Feliz/Animado(a)/Bem-humorado(a) do que Triste/Desanimado(a)/Mal-humorado(a), Mais Tranquilo(a)/Satisfeito(a) do que Tenso(a)/Estressado(a)	-1,3714	0,615	-2,6153365	-0,1275207	0,0316

Assim, trabalhar no período noturno aumenta em média 1,6 a quantidade de itens assinalados na parte B em recursos laborais.
Estar Mais Feliz/Animado(a)/Bem-humorado(a) do que Triste/Desanimado(a)/Mal-humorado(a), Mais Tranquilo(a)/Satisfeito(a) do que Tenso(a)/Estressado(a) diminui em média 1,37 a quantidade de itens assinalados na parte B em recursos laborais

Tabela 12: RVC dos itens do questionário calculado para cada etapa

**item**	**RVC - Rodada 1**	**RVC - Rodada 2**
es_a0	0,6	1
ei_a0	0,2	1
ee_a0	-0,2	1
ec_a0	-0,2	1
es_a1	0,6	0,6
ei_a1	0,2	0,6
ee_a1	0,2	1
ec_a1	0,2	0,2
es_a2	1	-
ei_a2	1	-
ee_a2	0,6	-
ec_a2	1	-
es_a3	0,6	-
ei_a3	0,6	-
ee_a3	1	-
ec_a3	1	-
es_a4	1	-

ei_a4	1	-
ee_a4	1	-
ec_a4	1	-
es_a5	0,2	1
ei_a5	0,2	1
ee_a5	0,2	1
ec_a5	0,6	1
es_a6	0,6	-
ei_a6	0,6	-
ee_a6	0,6	-
ec_a6	0,6	-
es_a7	1	-
ei_a7	1	-
ee_a7	0,6	-
ec_a7	1	-
es_a8	1	-
ei_a8	1	-
ee_a8	1	-
ec_a8	1	-
es_a9	1	-
ei_a9	0,6	-
ee_a9	0,6	-
ec_a9	1	-
es_a10	1	-
ei_a10	1	-
ee_a10	1	-
ec_a10	1	-
es_a11	1	-
ei_a11	1	-
ee_a11	1	-
ec_a11	1	-
es_a12	0,6	-
ei_a12	1	-
ee_a12	1	-
ec_a12	1	-
es_a13	1	-
ei_a13	1	-
ee_a13	1	-
ec_a13	1	-
es_a14	1	-
ei_a14	1	-
ee_a14	1	-
ec_a14	1	-
es_a15	1	-
ei_a15	0,6	-
ee_a15	0,6	-
ec_a15	1	-
es_a16	0,6	1

ei_a16	0,2	1
ee_a16	0,6	1
ec_a16	0,6	1
es_a17	1	-
ei_a17	0,6	-
ee_a17	0,6	-
ec_a17	1	-
es_a18	1	-
ei_a18	1	-
ee_a18	1	-
ec_a18	1	-
es_a19	1	-
ei_a19	1	-
ee_a19	1	-
ec_a19	1	-
es_a20	1	-
ei_a20	1	-
ee_a20	1	-
ec_a20	1	-
es_a21	0,6	-
ei_a21	0,6	-
ee_a21	0,6	-
ec_a21	1	-
es_a22	0,6	-
ei_a22	0,6	-
ee_a22	0,6	-
ec_a22	1	-
es_a23	1	-
ei_a23	0,6	-
ee_a23	0,6	-
ec_a23	1	-
es_a24	1	-
ei_a24	1	-
ee_a24	0,6	-
ec_a24	1	-
es_a25	1	-
ei_a25	1	-
ee_a25	1	-
ec_a25	1	-
es_a26	1	-
ei_a26	0,6	-
ee_a26	0,6	-
ec_a26	1	-
es_a27	1	-
ei_a27	1	-
ee_a27	1	-
ec_a27	1	-

es_a28	0,6	0,6
ei_a28	0,2	1
ee_a28	0,2	1
ec_a28	0,6	0,6
es_a29	1	-
ei_a29	0,6	-
ee_a29	1	-
ec_a29	1	-
es_b0	1	-
ei_b0	1	-
ee_b0	1	-
ec_b0	1	-
es_sb1	1	-
ei_sb1	0,6	-
ee_sb1	0,6	-
ec_sb1	0,6	-
es_b1	1	-
ei_b1	1	-
ee_b1	0,6	-
ec_b1	0,6	-
es_b2	0,6	1
ei_b2	0,6	1
ee_b2	0,6	1
ec_b2	0,6	1
es_b3	1	-
ei_b3	0,6	-
ee_b3	0,6	-
ec_b3	0,6	-
es_b4	1	-
ei_b4	1	-
ee_b4	1	-
ec_b4	1	-
es_b5	1	-
ei_b5	1	-
ee_b5	1	-
ec_b5	1	-
es_b6	1	-
ei_b6	1	-
ee_b6	1	-
ec_b6	1	-
es_sb2	1	-
ee_sb2	1	-
ei_sb2	1	-
ec_sb2	1	-
es_b7	0,6	0,6
ei_b7	0,2	1
ee_b7	0,2	1
ec_b7	0,6	0,6

es_b8	0,6	1
ei_b8	0,6	1
ee_b8	0,6	1
ec_b8	0,6	1
es_b9	0,6	0,6
ei_b9	0,6	1
ee_b9	0,2	1
ec_b9	0,6	0,6
es_b10	0,6	0,6
ei_b10	0,6	1
ee_b10	-0,2	1
ec_b10	0,6	0,6
es_b11	0,6	1
ei_b11	0,6	1
ee_b11	0,6	1
ec_b11	0,6	1
es_b12	1	-
ei_b12	1	-
ee_b12	1	-
ec_b12	1	-
es_b13	1	-
ei_b13	1	-
ee_b13	0,6	-
ec_b13	1	-
es_sb3	1	-
ei_sb3	1	-
ee_sb3	1	-
ec_sb3	1	-
es_b14	1	-
ei_b14	1	-
ee_b14	1	-
ec_b14	1	-
es_b15	1	-
ei_b15	1	-
ee_b15	1	-
ec_b15	1	-
es_b16	1	-
ei_b16	1	-
ee_b16	1	-
ec_b16	1	-
es_b17	0,6	1
ei_b17	0,6	1
ee_b17	0,6	1
ec_b17	0,6	1

Pela tabela 12, nota-se que o RVC dos itens reformulados melhoraram de uma etapa para outra.

O alpha de cronbach da primeira rodada é 0,826, indicando uma boa consistência.

O alpha de cronbach da segunda rodada é 0,804, indicando uma consistência aceitável apesar do decaimento de uma rodada para outra. Observa-se pelo RVC que os itens reavaliados passaram a ter mais avaliadores considerando o item essencial após os ajustes.
Apenas o item ec_a1 possui um RVC baixo, mas pelo IVC se apresenta com concordância de 60% entre os avaliadores. Assim, para todos os itens reavaliados, exceto o item ec_a1, o RVC e IVC se apresentam com valores de pelo menos 60%.
