# Supplementary material for: Translation and cross-cultural adaptation of the MISSCARE Survey-Ped into Brazilian Portuguese
Source: Rev Bras Enferm. 2024 Jul 19;77(2):e20230060. doi: 10.1590/0034-7167-2023-0060 (PMC11259437; doi:10.1590/0034-7167-2023-0060)
Supplement: 0034-7167-reben-77-02-e20230060-suppl04 [file 0034-7167-reben-77-02-e20230060-suppl04.pdf]

Tradução e adaptação transcultural do instrumento MISSCARE Survey-Ped para o português brasileiro

Julia Silva Del Bello, Kiana Alexandra Rei Gray, Mavilde da Luz Gonçalves

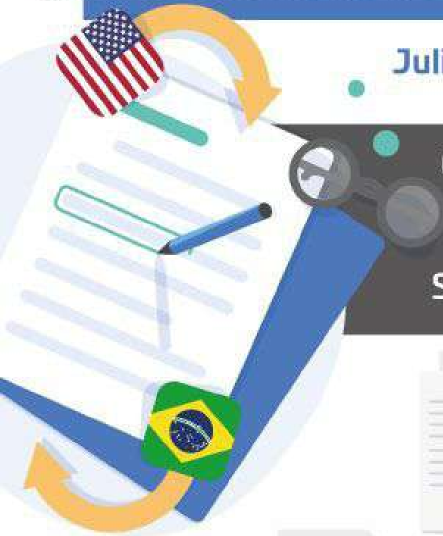

Objetivo: Realizar a tradução e adaptação transcultural do instrumento MISSCARE Survey-Ped para uso no Brasil.

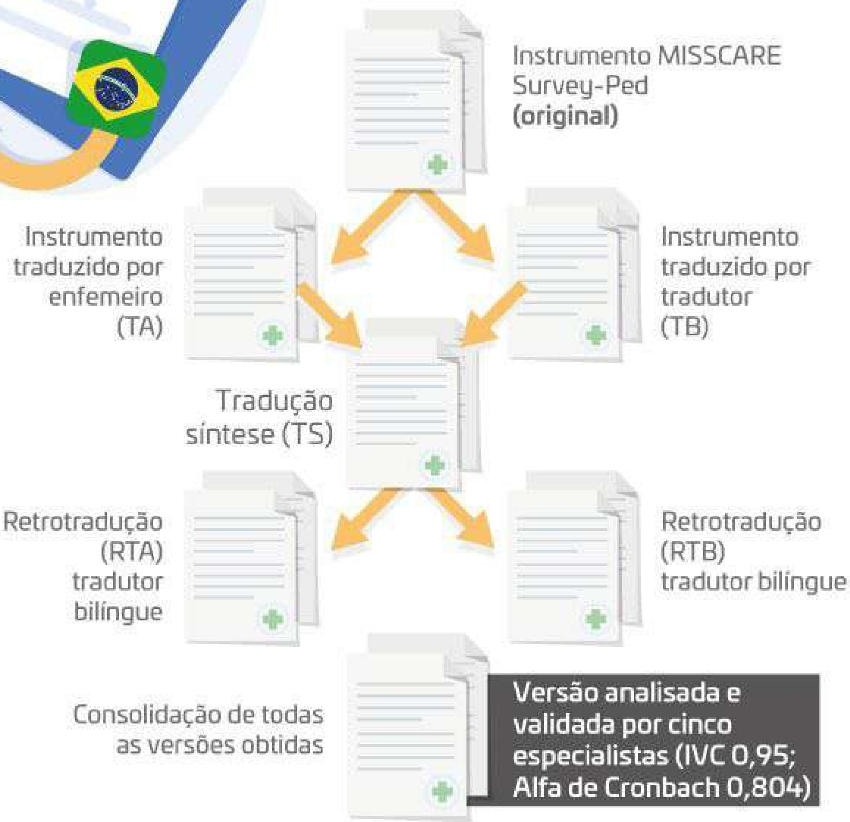

PARTICIPARAM DO PRÉ-TESTE:

44 enfermeiros pediatras da prática clínica brasileira.

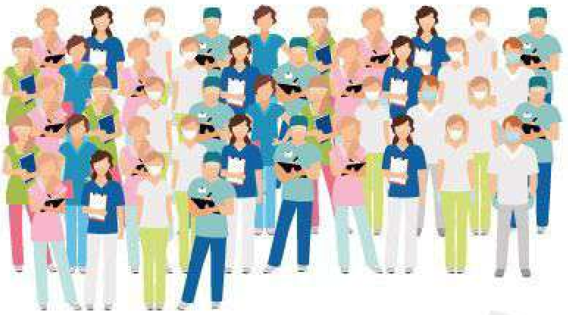

Cada participante analisou o instrumento assinalando os cuidados de enfermagem não realizados e quais as razões, baseando-se em situação vivenciada em sua prática clínica.

Calculou-se o IVC e RVC na avaliação da compreensão (IVC 0,886; RVC de 0,773), pertinência (IVC 0,931; RVC 0,864) e relevância (IVC 0,977; RVC 0,995) do instrumento.

O *MISSCARE Survey-Ped* Brasil foi obtido a partir da tradução e adaptação transcultural do instrumento original, sendo considerado apto para aplicação na prática clínica de enfermeiros pediatras do país.

Processo FAPESP nº 2021/05980-0
