# Supplementary material for: Translation and cross-cultural adaptation of the MISSCARE Survey-Ped into Brazilian Portuguese
Source: Rev Bras Enferm. 2024 Jul 19;77(2):e20230060. doi: 10.1590/0034-7167-2023-0060 (PMC11259437; doi:10.1590/0034-7167-2023-0060)
Supplement: 0034-7167-reben-77-02-e20230060-suppl05 [file 0034-7167-reben-77-02-e20230060-suppl05.pdf]

MISSCARE Survey Ped original	Tradução A (TA)	Tradução B (TB)	Síntese (TS)	Final
**Section "A" – missed nursing care activities**	**Seção A – atividades de cuidados omitidos de enfermagem**	**Seção "A" – atividades de cuidados de enfermagem não realizadas**	**Seção "A" – atividades de cuidados de enfermagem não realizadas**	**Seção "A" – Cuidados de enfermagem não realizados**
Attendance at daily rounds at the bedside	Visita de enfermagem à beira leito diariamente	Atendimento em rondas diárias à beira leito	Participação na visita clínica diária à beira leito	Participação na visita clínica multiprofissional diária à beira leito
Ambulation 3 times per day or as per nursing plan, if clinical conditions allows	Deambulação 3 vezes ao dia ou de acordo com a prescrição de enfermagem, se as condições clínicas permitirem	Deambulação 3 vezes por dia ou de acordo com o plano de enfermagem, se as condições clínicas permitirem	Deambulação 3 vezes ao dia ou de acordo com o plano de cuidados de enfermagem, se as condições clínicas permitirem	Deambulação 3 vezes ao dia ou de acordo com o plano de cuidados de enfermagem, se as condições clínicas permitirem
Assess effectiveness of medications	Avaliação da eficácia dos medicamentos	Avaliar a efetividade dos medicamentos	Avaliação da eficácia da medicação	Avaliação da eficácia da medicação
Passive mobilization of child every 2 hr or as ordered	Mudança do decúbito da criança a cada 2 horas ou conforme prescrito	Mobilização passiva da criança a cada 2 horas ou conforme solicitado	Mudança de decúbito da criança a cada 2 horas ou conforme prescrito	Mudança de decúbito da criança a cada 2 horas ou conforme prescrito
Mouth care	Cuidados orais	Cuidados bucais	Cuidados orais	Cuidados bucais
Parents included in child's care	Pais incluídos nos cuidados com a criança	Pais envolvidos no cuidado da criança	Envolvimento dos pais nos cuidados com a criança	Envolvimento dos pais nos cuidados com a criança
Patient and family education	Educação em saúde para o paciente e família	Educação do paciente e família	Educação do paciente e família	Educação do paciente e família
Discussion with child and his/her family about plans for discharge and care at home	Discussão com a criança e sua família sobre planos de alta e cuidados em casa	Conversa com a criança e sua família sobre planos de alta e de cuidados em casa	Discussão com a criança e sua família sobre planos de alta e cuidados no domicílio	Discussão com a criança e sua família sobre planos de alta e cuidados no domicílio
Promote neuro-evolutionary develop, according to age and baby's	Promoção do desenvolvimento neuroevolutivo, de acordo com a idade e condições clínicas (ou seja,	Promover o desenvolvimento neuro evolutivo, de acordo com a idade e as condições clínicas do bebê	Promoção do desenvolvimento neuroevolutivo, de acordo com a idade e condições clínicas da	Promoção do desenvolvimento neuroevolutivo, de acordo com a idade e condições clínicas da criança (por exemplo, cuidados neonatais,

clinical conditions (i.e., neonatal care, cognitive and relational development in child or in adolescent)	cuidados neonatais, desenvolvimento cognitivo e relacional na criança ou no adolescente)	(ou seja, cuidados neonatais, desenvolvimento cognitivo e relacional na criança ou no adolescente)	criança (por exemplo, cuidados neonatais, desenvolvimento cognitivo e relacional da criança ou do adolescente)	desenvolvimento cognitivo e relacional da criança ou do adolescente)
Pain assessment with pharmacological or with non-pharmacological care approaches, according to protocol	Avaliação da dor com abordagens farmacológicas ou não farmacológicas, de acordo com o protocolo	Avaliação da dor com abordagens farmacológicas ou não farmacológicas, de acordo com o protocolo	Avaliação da dor e intervenções farmacológicas ou não farmacológicas, de acordo com protocolos	Avaliação da dor e intervenções farmacológicas ou não farmacológicas, de acordo com protocolos
Medication requests acted on in 15 min	Solicitações de medicamentos atendidas dentro de 15 minutos	Pedidos de medicação atendidos em 15 min	Solicitações de medicamentos atendidas dentro de 15 minutos	Solicitações de medicamentos atendidas dentro de 15 minutos
Full documentation of all necessary data	Documentação completa de todos os dados necessários	Documentação completa de todos os dados necessários	Documentação completa de todos os dados necessários	Documentação completa com todos os dados necessários
Communicati on of all relevant information during shift change or handover	Comunicação de todas as informações relevantes na passagem de plantão ou transferência	Comunicação de todas as informações relevantes durante a mudança de turno ou transferência	Comunicação de todas as informações relevantes na passagem de plantão ou transferência	Comunicação de todas as informações relevantes na passagem de plantão ou transferência
Satisfaction of eating need according to child's clinical conditions (i.e., encourages oral feeding and/or nutrition at the request on the newborn; encourage a correct alimentation in according of personal taste)	Satisfação da necessidade alimentar de acordo com as condições clínicas da criança (ou seja, incentivo a alimentação oral e/ou nutricional a pedido do recém-nascido; incentivo a alimentação correta de acordo com o gosto pessoal)	Satisfação da necessidade alimentar de acordo com as condições clínicas da criança (ou seja, incentiva a alimentação oral e/ou nutricional a pedido do recém-nascido; incentiva uma alimentação correta de acordo com o gosto pessoal)	Satisfação das necessidades alimentares, de acordo com as condições clínicas da criança (por exemplo, incentivo a alimentação oral e/ou nutrição do recém-nascido assim que solicitado; incentivo a alimentação	Satisfação das necessidades alimentares, de acordo com as condições clínicas da criança (por exemplo, incentivo a alimentação oral e/ou nutrição do recém-nascido assim que solicitado; incentivo a alimentação apropriada, de acordo com a preferência pessoal)

			apropriada, de acordo com a preferência pessoal)	
Medications administered in 30 min before or after scheduled time (i.e., scheduled time 8 p.m., administration between 7:30 p.m. and 8:30 p.m.)	Administração de medicamentos 30 minutos antes ou depois do horário prescrito (ou seja, horário prescrito às 20h, administração entre 19h30 e 20h30)	Medicamentos administrados 30 minutos antes ou depois do horário programado (ou seja, horário programado às 20h, administração entre 19h30 e 20h30)	Administração de medicamentos 30 minutos antes ou depois do horário programado (por exemplo, horário programado às 20h, administração entre 19h30 e 20h30)	Administração de medicamentos entre 30 minutos antes ou depois do horário programado (por exemplo, horário programado às 20h, administração entre 19h30 e 20h30)
Assist child with toileting needs in 5 min of request (i.e., go with baby to the toilet or give appropriate devices if bedridden)	Auxílio a criança nas necessidades em banheiro dentro de 5 minutos após a solicitação (ou seja, ir com o bebê ao banheiro o dar os dispositivos apropriados se estiver acamado)	Auxiliar a criança nas necessidades de banheiro em 5 minutos após a solicitação (ou seja, ir com o bebê ao banheiro ou dar os dispositivos apropriados se estiver acamada)	Auxílio a criança nas necessidades de eliminação dentro de 5 minutos após a solicitação (por exemplo, ir com a criança ao banheiro ou fornecer os dispositivos apropriados se estiver no leito)	Auxílio a criança nas necessidades de eliminação dentro de 5 minutos após a solicitação (por exemplo, ir com a criança ao banheiro ou fornecer os dispositivos apropriados se estiver restrita no leito)
Response to call light, to intervention request, or alarm is initiated in 5 min (i.e,. monitor, infusion pumps, ventilator)	Resposta à luz de chamada, à solicitação de intervenção ou alarme é iniciada dentro de 5 minutos (ou seja, monitor, bombas de infusão, ventilador)	Reposta à luz de chamada, à solicitação de intervenção ou alarme iniciada em 5 min (ou seja, monitor, bombas de infusão, ventilador)	Resposta à luz de chamada, à solicitação de intervenção ou alarme é iniciada dentro de 5 minutos (por exemplo, monitores, bombas de infusão, aparelhos de ventilação mecânica)	Resposta à luz de chamada, à solicitação de intervenção ou alarme é iniciada dentro de 5 minutos (por exemplo, monitores, bombas de infusão, aparelhos de ventilação mecânica)
Emotional support to child and/ or family	Suporte emocional para a criança e sua família	Apoio emocional à criança e/ou família	Apoio emocional à criança e/ou família	Apoio emocional à criança e/ou família
Labs/specimen obtained as ordered	Coleta de exames laboratoriais obtidos conforme solicitado	Laboratoriais/espécimes obtidos conforme solicitado	Coleta de exames laboratoriais realizados conforme prescrito	Coleta de exames laboratoriais realizados conforme prescrito

Body hygiene and skin care	Higiene corporal e cuidados com a pele	Higiene corporal e cuidados com a pele	Higiene corporal e cuidados com a pele	Higiene corporal e cuidados com a pele
Central line site and peripheral line site assessment per protocol	Avaliação do local de inserção do cateter central e do cateter periférico segundo o protocolo	Avaliação do local da linha central e do local da linha periférica por protocolo	Avaliação do local de inserção do cateter intravenosos central e do cateter intravenoso periférico segundo protocolos	Avaliação do local de inserção do cateter intravenosos central e do cateter intravenoso periférico segundo protocolos
Central line site and peripheral line site care per protocol	Cuidados com o local de inserção do cateter central e do cateter periférico segundo o protocolo	Cuidados com o local da linha central e local da linha periférica de acordo com o protocolo	Cuidados com o local de inserção do cateter intravenoso central e do cateter intravenoso periférico segundo protocolos	Cuidados com o local de inserção do cateter intravenoso central e do cateter intravenoso periférico segundo protocolos
Adoption of the necessary precautions for infections control as per protocol (Individual Protection Devices, devices disinfection, isolation, correct waste disposal)	Adoção das precauções necessárias para o controle de infecções conforme protocolo (uso de EPIs, desinfecção de dispositivos, isolamento, correto descarte de resíduos)	Adoção dos cuidados necessários para o controle de infecções conforme protocolo (Dispositivos de Proteção Individual, desinfecção de dispositivos, isolamento, destinação correta de resíduos)	Adoção das precauções necessárias para o controle de infecções conforme protocolos (uso de EPIs, desinfecção de dispositivos, isolamento, correto descarte de resíduos)	Adoção das precauções necessárias para o controle de infecções conforme protocolos (uso de EPIs, desinfecção de dispositivos, isolamento, correto descarte de resíduos)
Monitoring intake/output of solid and liquid	Monitoramento da entrada/saída de sólidos e líquidos	Monitorar a entrada/saída de sólidos e líquidos	Monitoramento dos ganhos e perdas de sólidos e líquidos	Monitoramento dos ganhos e perdas de sólidos e líquidos
Vital signs assessed according to the nursing plan	Avaliação dos sinais vitais de acordo com a prescrição de enfermagem	Sinais vitais avaliados de acordo com o plano de enfermagem	Avaliação dos sinais vitais de acordo com o plano de cuidados de enfermagem	Avaliação dos sinais vitais de acordo com o plano de cuidados de enfermagem

Focused reassessments of the child's condition to assess improvements or deterioration during the shift	Reavaliações direcionadas sobre a condição da criança para avaliar melhorias ou pioras durante o turno	Reavaliações focadas da condição da criança para avaliar melhorias ou deterioração durante o turno	Reavaliações direcionadas sobre a condição da criança para avaliar melhorias ou agravos durante o plantão	Reavaliações direcionadas sobre a condição da criança para avaliar melhorias ou agravos durante o plantão
Hand washing	Lavagem das mãos	Lavagem das mãos	Higienização das mãos	Higienização das mãos
Assessment of the activities attributed to caregiver	Avaliação das atividades atribuídas ao cuidador	Avaliação das atividades atribuídas ao cuidador	Avaliação das atividades realizadas pelo cuidador	Avaliação das atividades atribuídas aos cuidadores
Safety and hygiene checks of bedside equipment completed once per shift or per protocol (i.e., bed, nightstand, devices)	Verificação de segurança dos equipamentos e limpeza concorrente do mobiliário realizadas uma vez por plantão ou segundo protocolo (ou seja, cama, mesa de cabeceira, dispositivos)	Verificações de segurança e higiene do equipamento de cabeceira concluídas uma vez por turno ou por protocolo (ou seja, cama, mesa de cabeceira, dispositivos)	Verificação de segurança dos equipamentos e limpeza concorrente do mobiliário realizadas uma vez por plantão ou segundo protocolo (por exemplo, cama, mesa de cabeceira, dispositivos)	Verificação de segurança dos equipamentos e limpeza concorrente do mobiliário realizadas uma vez por plantão ou segundo protocolo (por exemplo, cama, mesa de cabeceira, dispositivos)
**Section “B” – reasons for missed nursing care**	**Seção B – razões para as omissões de cuidados em enfermagem**	**Seção “B” – razões para atividades de cuidados de enfermagem não realizadas**	**Seção “B” – razões para as omissões de cuidados em enfermagem**	**Seção “B” – razões para as omissões de cuidados em enfermagem**
Labour resources	*Recursos de trabalho*	*Recursos de trabalho*	*Recursos laborais*	*Recursos laborais*
Unbalanced patient assignments**;**	Desequilíbrio nas atribuições de pacientes;	Atribuições desequilibradas de pacientes;	Desequilíbrio nas atribuições com pacientes.	Desequilíbrio no dimensionamento de pacientes.
Inadequate number of nurses**;**	Número inadequado de enfermeiras;	Número inadequado de enfermeiras;	Número inadequado de enfermeiras;	Número inadequado de enfermeiros.
Urgent patient situation (i.e., a patient's condition worsening)**;**	Pacientes em situação urgente (ou seja, piora da condição do paciente);	Situação urgente do paciente (ou seja, piora da condição do paciente);	Situação de urgência do paciente (por exemplo, piora da condição do paciente).	Situação de urgência do paciente (por exemplo, piora da condição do paciente).

Unexpected rise Inpatient volume and/or acuity on the unit**;**	Aumento inesperado do volume e/ou gravidade dos pacientes na unidade;	Aumento inesperado do volume e/ou acuidade de pacientes na unidade;	Aumento inesperado do número e/ou gravidade dos pacientes na unidade.	Aumento inesperado do número e/ou gravidade dos pacientes na unidade.
Inadequate number of assistive (i.e., nursing assistants)**;**	Número inadequado de técnicos/auxiliar es de enfermagem;	Número inadequado de auxiliares (ou seja, auxiliares de enfermagem);	Número inadequado de técnicos/auxiliares de enfermagem.	Número inadequado de técnicos/auxiliares de enfermagem.
Frequent interruptions;	Interrupções frequentes;	Interrupções frequentes;	Interrupções frequentes;	Interrupções frequentes.
Communication	*Comunicação*	*Comunicação*	*Comunicação*	*Comunicação*
Tension or communica tion breakdowns with the medical staff;	Barreiras de tensão ou comunicação com a equipe médica;	Tensão ou quebra de comunicação com a equipe médica;	Tensão ou barreiras de comunicação com a equipe médica.	Tensão ou falhas na comunicação com a equipe médica.
Lack of collaboration from team members (i.e., nurses, nursing assistant, physicians);	Falta de colaboração dos membros da equipe (ou seja, enfermeiras, técnicos/auxiliar es, médicos);	Falta de colaboração dos membros da equipe (ou seja, enfermeiros, auxiliares de enfermagem, médicos);	Falta de colaboração entre membros da equipe (por exemplo, enfermeiras, técnicas/auxiliares de enfermagem e médicos).	Falta de colaboração entre membros da equipe (por exemplo, enfermeiros, técnicas/auxiliares de enfermagem e médicos).
Tension or communica tion breakdowns in the nursing team;	Barreiras de tensão ou comunicação com a equipe de enfermagem;	Tensão ou falhas de comunicação na equipe de enfermagem;	Tensão ou barreiras de comunicação na equipe de enfermagem.	Tensão ou falhas na comunicação na equipe de enfermagem.
Tension or communica tion breakdowns with other services or departments (i.e., transfusion centre, radio diagnostic service, hospital pharmacy, etc.);	Barreiras de tensão ou comunicação com outros serviços ou departamentos (ou seja, banco de sangue, serviço de radiologia, farmácia, etc);	Tensão ou quebra de comunicação com outros serviços ou departamentos (ou seja, centro de transfusão, serviço de diagnóstico de rádio, farmácia hospitalar, etc.);	Tensão ou barreiras de comunicação com outros serviços ou departamentos (por exemplo, banco de sangue, serviço de radiologia, farmácia, etc);	Tensão ou falhas na comunicação com outros serviços ou departamentos (por exemplo, banco de sangue, serviço de radiologia, farmácia, etc);
Nursing assistant did not communicate that care to the child was not done;	Auxiliar/técnico de enfermagem não comunicou que o cuidado à criança	Auxiliar de enfermagem não comunicou que o cuidado à criança não foi realizado;	Técnica/auxiliar de enfermagem não comunicou que o cuidado à criança não foi realizado.	Técnico/auxiliar de enfermagem não comunicou que o cuidado à criança não foi realizado.

	não foi realizado;			
Inadequate hand-off from previous shift or sending unit;	Inadequada passagem de plantão entre turnos ou na transferência entre unidades;	Transferência inadequada do turno anterior ou unidade emissora;	Inadequada passagem de plantão entre turnos ou na transferência entre unidades.	Inadequada passagem de plantão entre turnos ou na transferência entre unidades.
Other services or departments did not give the care needed (i.e., laboratory for analysis, hospital pharmacy);	Outros serviços ou departamentos não prestaram os cuidados necessários (ou seja, laboratório para análise, farmácia hospitalar);	Outros serviços ou departamentos não prestaram os cuidados necessários (ou seja, laboratório para análises, farmácia hospitalar);	Outros serviços ou departamentos não prestaram os cuidados necessários (por exemplo, laboratório de análises, farmácia hospitalar).	Outros serviços ou setores não prestaram os cuidados necessários (por exemplo, laboratório de análises, farmácia hospitalar).
Material resources	*Recursos materiais*	*Recursos materiais*	*Recursos materiais*	*Recursos materiais*
Supplies/equipment not available when needed (i.e., infusion pumps, surgical tools);	Suprimentos/equipamentos não disponíveis quando necessário (ou seja, bombas de infusão, instrumentos cirúrgicos);	Suprimentos/equipamentos não disponíveis quando necessários (ou seja, bombas de infusão, ferramentas cirúrgicas);	Materiais/equipamentos não disponíveis quando necessários (por exemplo, bombas de infusão, instrumentais cirúrgicos).	Materiais/equipamentos não disponíveis quando necessários (por exemplo, bombas de infusão, instrumentais cirúrgicos).
Supplies/equipment not properly functioning when needed;	Suprimentos/equipamentos não funcionam corretamente quando necessário;	Suprimentos/equipamentos não estão funcionando corretamente quando necessário;	Materiais/equipamentos não funcionam corretamente quando necessário.	Materiais/equipamentos não funcionam corretamente quando necessário.
Medications were not available when needed;	Medicamentos não estavam disponíveis quando necessário;	Os medicamentos não estavam disponíveis quando necessários ;	Medicamentos não disponíveis quando necessários.	Medicamentos não disponíveis quando necessários.
Lack of familiarity with equipment/ procedure/p olicy.	Falta de familiaridade com o equipamento/procedimento/política.	Falta de familiaridade com o equipamento/procedimento/política.	Falta de treinamento com equipamento/procedimento/normas.	Falta de familiaridade com equipamento/procedimento/norma.

Retrotradução A (RTA)	Retrotradução B (RTB)
**Section "A" - nursing care activities not performed**	**Section "A" - nursing care activities not performed**
Participation in the daily clinical visit to the bedside	Participation in the daily clinical visit to the bedside
Ambulation 3 times a day or according to the nursing care plan, if clinical conditions allow	Ambulation 3 times a day or according to the nursing care plan, if clinical conditions allow
Evaluation of the efficacy of the medication	Evaluation of the efficacy of the medication
Change of child's decubitus every 2 hours or as prescribed	Change of child's decubitus every 2 hours or as prescribed
Oral care	Oral care
Parental involvement in childcare	Parental involvement in childcare
Patient and family education	Patient and family education
Discussion with the child and family about discharge plans and home care	Discussion with the child and his/her family about discharge plans and home care
Promotion of neuroevolutionary development, according to the child's age and clinical conditions (e.g. neonatal care, cognitive and relational development of the child or adolescent)	Promotion of neuroevolutionary development, according to the age and clinical conditions of the child (e.g., neonatal care, cognitive and relational development of the child or adolescent)
Pain assessment and pharmacological or non pharmacological interventions, according to protocols	Pain assessment and pharmacological or non pharmacological interventions, according to protocols
Drug requests met within 15 minutes	Drug requests met within 15 minutes
Complete documentation of all necessary data	Complete documentation of all necessary data

Communication of all relevant information on shift change or transfer	Communication of all relevant information on shift change or transfer
Satisfying dietary needs, according to the child's clinical conditions (e.g., encouraging oral feeding and/or nutrition of the newborn as requested; encouraging appropriate feeding, according to personal preference)	Satisfaction of dietary needs, according to the clinical conditions of the child (e.g., encouraging oral feeding and/or nutrition of the newborn as soon as requested; encouraging appropriate feeding, according to personal preference)
Administration of medicines 30 minutes before or after the scheduled time (e.g., scheduled time at 8:00 p.m., administration between 7:30 p.m. and 8:30 p.m.)	Administration of medicines 30 minutes before or after the scheduled time (e.g., scheduled time at 8:00 p.m., administration between 7:30 p.m. and 8:30 p.m.)
Assist the child in the disposal needs within 5 minutes after the request (e.g., go with the child to the bathroom or provide the appropriate devices if he is in bed)	Assist the child in the disposal needs within 5 minutes after the request (e.g., go with the child to the bathroom or provide the appropriate devices if he is in bed)
Response to call light, intervention request or alarm is initiated within 5 minutes (e.g. monitors, infusion pumps, mechanical ventilation appliances)	Response to call light, intervention request or alarm is initiated within 5 minutes (e.g. monitors, infusion pumps, mechanical ventilation appliances)
Emotional support for the child and/or family	Emotional support for the child and/or family
Collection of laboratory tests performed as prescribed	Collection of laboratory tests performed as prescribed
Body hygiene and skin care	Body hygiene and skin care
Evaluation of the insertion site of the central intravenous catheter and peripheral intravenous catheter according to protocols	Evaluation of the insertion site of the central intravenous catheter and peripheral intravenous catheter according to protocols
Care with the insertion area of the central intravenous catheter and peripheral intravenous catheter according to protocols	Care with the insertion area of the central intravenous catheter and peripheral intravenous catheter according to protocols
Adoption of the necessary precautions for infection control according to protocols (use of EPIs, disinfection of devices, isolation, correct disposal of waste)	Adoption of the necessary precautions for infection control according to protocols (use of EPIs, disinfection of devices, isolation, correct disposal of waste)
Monitoring solids and liquids gains and losses	Monitoring solids and liquids gains and losses

Evaluation of vital signs according to the nursing care plan	Evaluation of vital signs according to the nursing care plan
Targeted reassessments of the child's condition to assess improvements or injuries during shift	Targeted reassessments of the child's condition to assess improvements or injuries during shift
Hand hygiene	Hand hygiene
Evaluation of the activities performed by the caregiver	Evaluation of the activities performed by the caregiver
Safety check of equipment and concurrent cleaning of furniture performed once per shift or according to protocol (e.g. bed, nightstand, devices)	Safety check of equipment and concurrent cleaning of furniture performed once per shift or according to protocol (e.g. bed, bedside table, devices)
**Section "B" - reasons for nursing care omissions**	**Section "B" - reasons for nursing care omissions**
Labor resources	*Labor resources*
Imbalance in patient assignments.	Imbalance in patient assignments.
Inadequate number of nurses.	Inadequate number of nurses.
Urgent situation of the patient (e.g., worsening of the patient's condition).	Urgent situation of the patient (e.g., worsening of the patient's condition).
Unexpected increase in the number and/or severity of patients in the unit.	Unexpected increase in the number and/or severity of patients in the unit.
Inadequate number of nursing technicians/aassistants.	Inadequate number of nursing technicians/aassistants.
Frequent interruptions.	Frequent interruptions.
Communication	*Communication*
Tension or communication barriers with medical team.	Tension or communication barriers with medical team.

Lack of collaboration between team members (e.g., nurses, nursing technicians/assistants and physicians).	Lack of collaboration between team members (e.g., nurses, nursing technicians/assistants and physicians).
Tension or communication barriers in the nursing team.	Tension or communication barriers in the nursing team.
Tension or communication barriers with other services or departments (e.g. blood bank, radiology service, pharmacy, etc.).	Tension or communication barriers with other services or departments (e.g. blood bank, radiology service, pharmacy, etc.).
A nursing technician/assistant did not report that childcare was not performed.	A nursing technician/assistant did not report that childcare was not performed.
Inadequate transfer between shifts or between units.	Inadequate transfer between shifts or between units.
Other services or departments did not provide the necessary care (e.g., analysis laboratory, hospital pharmacy).	Other services or departments did not provide the necessary care (e.g., analysis laboratory, hospital pharmacy).
Material resources	*Material resources*
Materials/equipment not available when needed (e.g., infusion pumps, surgical instruments).	Materials/equipment not available when needed (e.g., infusion pumps, surgical instruments).
Materials/equipment not working properly when needed.	Materials/equipment not working properly when needed.
Medicines not available when needed.	Medicines not available when needed.
Lack of training with equipment/ procedure/ standards.	Lack of training with equipment/ procedure/ standards.
