# Supplementary material for: Translation and cross-cultural adaptation of the MISSCARE Survey-Ped into Brazilian Portuguese
Source: Rev Bras Enferm. 2024 Jul 19;77(2):e20230060. doi: 10.1590/0034-7167-2023-0060 (PMC11259437; doi:10.1590/0034-7167-2023-0060)
Supplement: Translation

# Translation: Tradução e adaptação transcultural do instrumento *MISSCARE Survey-Ped* para o português brasileiro

**DOI:** 10.1590/0034-7167-2023-0060pt

**Authors:** Julia Silva Del Bello, Kiana Alexandra Rei Gray, Mavilde da Luz Gonçalves Pedreira

**Affiliations:** I Universidade Federal de São Paulo. São Paulo, São Paulo, Brasil; II Conselho Nacional de Desenvolvimento Científico e Tecnológico. Brasília, Distrito Federal, Brasil

**Keywords:** Enfermagem Pediátrica, Segurança do Paciente, Cuidados de Enfermagem, Estudos de Validação, Carga de Trabalho.

## Abstract

**Objetivos:**

realizar a tradução e adaptação transcultural do instrumento *MISSCARE Survey-Ped* para uso no Brasil.

**Métodos:**

estudo metodológico proposto por tradução, síntese das traduções, retrotradução, avaliação por comitê de especialistas e pré-teste com a população-alvo.

**Resultados:**

foram realizadas duas traduções diretas do instrumento, seguidas por uma versão consensual entre as mesmas. Essa versão síntese foi retrotraduzida e analisada por um comitê de cinco especialistas em enfermagem pediátrica e segurança do paciente, obtendo um Índice de Validade de Conteúdo (IVC) de 0,95 e alfa de Cronbach de 0,804. Enviou-se a versão final para pré-teste com 254 enfermeiros pediatras brasileiros, sendo que 44 (17,3%) analisaram o instrumento quanto à sua compreensão (IVC 0,866; Razão de Validade de Conteúdo (RVC) 0,773), pertinência (IVC 0,931; RVC 0,864) e relevância (IVC 0,977; RVC 0,955).

**Conclusões:**

o *MISSCARE Survey-Ped* Brasil foi considerado apto para aplicação na prática clínica de enfermeiros pediatras do país.

## INTRODUÇÃO

Reduzir a possibilidade de erros e violações capazes de gerar eventos adversos ao paciente durante a prestação de assistência à saúde constitui um pilar para a promoção da segurança do paciente^(1)^. Contudo, não se organiza um sistema de saúde sem considerar que seres humanos são passíveis de erro. Ao invés de buscar a perfeição, a cultura de segurança do paciente visa identificar erros, gerenciar riscos, aprender com os erros e as omissões, visando instituir medidas de prevenção que promovam a segurança ao paciente e ao sistema como um todo^(1-2)^.

A tipologia e a abrangência dos erros na área da saúde estão sendo continuamente conhecidas por meio da pesquisa e da divulgação de dados oriundos de notificações estimuladas nas políticas de segurança na área da saúde da Organização Mundial da Saúde e do Ministério da Saúde^(3)^. O conhecimento sobre as situações que comprometem a segurança do paciente gera oportunidades para a instituição de medidas de controle e prevenção que tornem o sistema de saúde cada vez mais confiável, por meio da remodelação de estruturas, tecnologias e processos de interação entre o sistema de atendimento, os profissionais de saúde e pacientes e famílias^(4)^.

Os erros e eventos adversos que acometem crianças podem diferir daqueles que ocorrem em adultos devido às características denominadas como 4Ds: demografia; desenvolvimento; dependência; e diferentes condições de saúde^(5-6)^. Em um mesmo ambiente de cuidados, profissionais de saúde atendem de recém-nascidos a adolescentes, com diferentes necessidades, tipos de tecnologias de cuidado e de atendimento, podendo requerer conhecimentos de diversas especialidades para atenção a agravos neurológicos, respiratórios, cardiovasculares, gastrointestinais, urológicos, entre outros, demandando amplo conhecimento da equipe em uma área cada vez mais especializada^(4)^. Destacam-se na pediatria os erros na medicação, sendo estimada nos Estados Unidos a proporção de 1,8 a 2,96 erros a cada 100 altas^(5-7)^.

Um cuidado científico e ético em enfermagem se fundamenta em realizar a assistência certa, da maneira certa, para a pessoa certa e no momento certo, pautando-se na melhor informação científica disponível para atender às necessidades integrais e individuais do paciente e sua família^(8)^. No entanto, as constantes falhas operacionais dos sistemas desviam as ações de enfermeiros para a resolução momentânea de dificuldades. Com tais demandas, que muitas vezes vão além do que os competem, os enfermeiros não conseguem focar na atenção à individualidade e integralidade do ser humano, delegando atividades em excesso para o nível técnico e comprometendo a capacidade de avaliação dos cuidados de enfermagem prestados^(9)^.

Dessa forma, em meio a múltiplas demandas e recursos limitados, enfermeiros podem abreviar cuidados, atrasá-los ou ainda, omiti-los. Em relação aos erros, existem atos de comissão (fazer algo errado) e atos de omissão (deixar de fazer algo certo). Embora seja mais comum se desconhecerem os erros de omissão, suas consequências podem constituir relevante ameaça à segurança do paciente^(10-11)^. Em definição, o cuidado de enfermagem não realizado se refere a qualquer aspecto do atendimento ao paciente que foi omitido (parcial ou totalmente) ou atrasado^(12-13)^.

Para interferir de modo a aumentar a segurança e, consequentemente, a qualidade da assistência, os enfermeiros devem primeiro identificar quais são os fatores que predispõem à ocorrência de omissões de cuidados de enfermagem, as barreiras que impedem a prestação de cuidados e as oportunidades de melhoria.

Kalisch e Williams^(12)^ desenvolveram um instrumento chamado *The Missed Nursing Care Survey*, o *MISSCARE Survey*. A ferramenta divide-se em três partes, sendo a primeira composta por dados demográficos dos enfermeiros e relativos à satisfação no trabalho. Na sequência, a Parte A apresenta os itens relativos aos cuidados não realizados, e a Parte B abrange as razões para a não realização desses cuidados, contendo 24 e 17 itens, respectivamente. A mensuração dos dados se dá por uma escala do tipo Likert de quatro pontos, que na Parte A varia de “raramente esquecido” a “sempre esquecido”, e na Parte B, de “não é uma razão” a “razão significativa”. A Parte A e a Parte B podem ser aplicadas de forma independente^(12-13)^.

Em 2012, o *MISSCARE Survey* foi adaptado e validado para uso no Brasil com o nome de *MISSCARE-BRASIL* ^(10)^. A disponibilização desse instrumento no Brasil tem como objetivo o uso de ferramentas com capacidade de avaliar a omissão dos cuidados de enfermagem, tendendo a favorecer a identificação de soluções para o fenômeno em questão e melhorando a prática clínica^(10)^. Até o momento, não existem outros instrumentos no contexto nacional que se proponham a avaliar esse fenômeno empiricamente. Contudo, o instrumento não abrange especificidades pediátricas, o que revela uma lacuna no conhecimento de cuidados negligenciados dentro da enfermagem pediátrica brasileira.

Tubbs-Cooley *et al*.^(14)^ exploraram em 2015 o cuidado de enfermagem não realizado no contexto neonatal. Para isso, precisaram adaptar a versão original do *MISSCARE Survey* considerando características específicas dos recém-nascidos que requerem diferentes cuidados de enfermagem, bem como seu planejamento, quando comparado aos adultos. Isso também se aplica ao contexto hospitalar pediátrico, que difere tanto do adulto como do neonatal, e uma nova versão para essa população foi desenvolvida na Itália em 2018, o *MISSCARE Survey-Ped* ^(15)^.

O processo de adaptação do instrumento finalizou com 29 cuidados de enfermagem pediátrica na Seção A e 17 motivos para omissão na Seção B, considerando aspectos específicos do ambiente pediátrico, como a presença constante de familiares ou cuidadores, que são ativamente envolvidos no processo de cuidado^(9,15)^. O instrumento foi validado com bom Índice de Validade de Conteúdo (IVC) (0,88) e alfa de Cronbach (0,912; 0,82; 0,807)^(15)^.

Os cuidados omissos em pediatria são de particular preocupação, devido à vulnerabilidade e especificidades dessa população, pois são menos capazes de promover sua própria segurança, e resultados negativos podem comprometer seriamente sua saúde. Dessa forma, propõe-se neste estudo realizar a tradução e adaptação transcultural do *MISSCARE Survey-Ped* para uso no Brasil.

## OBJETIVOS

Realizar a tradução e adaptação transcultural do instrumento *MISSCARE Survey-Ped* para uso no Brasil.

## MÉTODOS

### Aspectos éticos

A autorização para uso do instrumento no Brasil, assim como sua tradução e adaptação, foi obtida junto à autora que desenvolveu o *MISSCARE Survey-Ped*. A pesquisa foi aprovada pelo Comitê de Ética em Pesquisa da Universidade Federal de São Paulo (CEP-UNIFESP), e todos os participantes do estudo assinaram o Termo de Consentimento Livre e Esclarecido (TCLE).

### Desenho, período e local do estudo

Trata-se de estudo metodológico composto por cinco etapas, tais como tradução, síntese das traduções, retrotradução, avaliação por comitê de especialistas, pré-teste de adaptação transcultural com a população-alvo, com base nas recomendações *Consensus-based Standards for the selection of health Measurement Instruments* (COSMIN)^(16)^.

Inicialmente, a tradução direta do instrumento foi realizada por dois tradutores independentes, com conhecimento do idioma original e do idioma de destino. Delineou-se que um dos tradutores fosse enfermeiro e estivesse ciente dos objetivos do estudo e dos conceitos envolvidos (TA), enquanto o outro não deveria ser enfermeiro nem conhecedor dos objetivos do estudo (TB), refletindo a linguagem usada na população leiga, conforme as recomendações de Beaton *et al*.^(17)^.

As traduções obtidas foram analisadas por profissional com conhecimento clínico e em pesquisa nas áreas de enfermagem pediátrica e segurança do paciente, assim como conhecimento avançado em inglês. Essa versão consensual foi reencaminhada aos tradutores iniciais para concordância da tradução síntese (TS).

A retrotradução foi implementada com participação de dois tradutores bilíngues (RTA e RTB), nativos na língua original e não especialistas na área de estudo, na tentativa de controle de viés e contribuir para encontrar erros ou incoerências na tradução. Todos os tradutores realizaram a atividade de modo independente. As versões RTA e RTB foram analisadas pelos autores do estudo até se obter consenso em inconsistências identificadas.

Um comitê de cinco *experts*, como é recomendado por Lynn^(18)^, formado por uma equipe de enfermeiros especialistas em enfermagem pediátrica, teve a função de consolidar todas as versões obtidas (TA, TB, TS, RTA, RTB) e desenvolver a versão pré-final do instrumento, considerando equivalência semântica (relativa ao significado das palavras pelo vocabulário e gramática), equivalência idiomática (ou seja, o uso de coloquialismos), equivalência experimental (que se refere às situações envolvidas no contexto cultural) e, por fim, equivalência conceitual (que explora as diferentes maneiras que um conceito pode ser entendido)^(17)^.

Esta versão final do *MISSCARE Survey-Ped* na língua portuguesa do Brasil foi analisada em estudo de adaptação transcultural. Foram convidados enfermeiros pediatras do Brasil, de diferentes regiões e níveis de formação, para análise da compreensão, pertinência e relevância para a área de enfermagem pediátrica e segurança do paciente.

Todos os dados foram coletados de forma virtual entre novembro de 2021 e julho de 2022.

### Amostra e critérios de inclusão

O recrutamento dos enfermeiros participantes da pesquisa ocorreu a partir de busca no currículo da Plataforma *Lattes* do Conselho Nacional de Desenvolvimento Científico e Tecnológico (CNPq), relação dos associados da Sociedade Brasileira de Enfermeiros Pediatras, contatos do grupo de pesquisas de enfermagem em segurança do paciente, cuidados intensivos pediátricos e terapia intravenosa em pediatria (SEGTEC - Segurança, Tecnologia e Cuidado) da UNIFESP e reconhecimento como especialista na área de enfermagem pediátrica ou segurança do paciente.

A seleção dos cinco membros para o comitê contou com os seguintes critérios de inclusão: profissionais da enfermagem brasileiros, com conhecimento avançado em inglês, experiência clínica e/ou de pesquisa nas áreas de enfermagem pediátrica e/ou segurança do paciente. Destaca-se que quatro (80,0%) enfermeiros possuíam titulação de doutorado e um (20,0%) de pós-doutorado.

A decisão do tamanho amostral para a aplicação do pré-teste considerou a abordagem de Beaton *et al*.^(17)^, sugerindo entre 20 e 40 pessoas do grupo-alvo. Ao todo, 254 enfermeiros brasileiros foram convidados para a aplicação do pré-teste por meio de uma carta convite enviada por e-mail, e 44 (17,3%) participaram da pesquisa.

### Protocolo do estudo

A coleta dos dados ocorreu de forma virtual e em duas etapas. A primeira foi desenvolvida entre novembro de 2021 e maio de 2022, envolvendo os processos de tradução, retrotradução e análise do comitê de especialistas. Para esse último, foi elaborado um formulário no *software Research Eletronic Data Capture* (REDCap)^®^ contendo a versão TS do instrumento para preenchimento das equivalências semântica, idiomática, experimental e cultural, sendo empregada a técnica Delphi para esta fase da pesquisa. Cada item apresentado pôde ser avaliado como “não equivale”, “indeciso” ou “equivale”, gerando uma escala Likert que pontuou -1, 0, 1, respectivamente. Nos casos de -1 ou 0, o *expert* deveria expressar sugestões de alteração no item avaliado. Aos especialistas, foram disponibilizadas também as outras versões (original, TA, TB, RTA e RTB) para consulta.

A segunda etapa da coleta ocorreu entre os meses de junho e julho de 2022, e teve início após a validação do instrumento pelo comitê. O formulário para os enfermeiros brasileiros foi elaborado na plataforma *Google Forms* ^®^. Solicitou-se para cada participante que analisasse o instrumento baseando-se ou simulando um plantão ou um momento em que realizou, supervisionou, vivenciou ou refletiu sobre a prática cotidiana de enfermagem no cuidado de neonatos, crianças, adolescentes e suas famílias, assinalando os cuidados de enfermagem não realizados. Ao final da Seção A e da Seção B, o enfermeiro poderia tecer considerações de adequação ou melhoria. Ao término das duas seções do *MISSCARE Survey-Ped*, foi solicitado que se realizasse a avaliação global, segundo uma escala Likert que variou de 1 a 5 pontos, considerando “discordo totalmente”, “discordo parcialmente”, “não concordo nem discordo”, “concordo parcialmente” e “concordo totalmente”. Em caso de discordância, solicitou-se ao enfermeiro que expressasse sugestões de melhoria.

Variáveis demográficas para identificação dos participantes do estudo também foram coletadas (idade, sexo, nível de formação acadêmica, cidade e estado onde trabalha, tempo de experiência profissional em anos, tempo de experiência na área de enfermagem pediátrica, área de atuação, atuação em instituição pública ou privada, turno de trabalho e jornada diária), além de informações relativas à satisfação no trabalho (o quão satisfeito está e como se sente no ambiente de trabalho), como é proposto no *MISSCARE* original.

### Análise dos resultados e estatística

Os resultados foram importados para uma planilha do programa *Microsoft Excel* ^®^, e foram analisados pelos autores do estudo. A fim de quantificar o grau de concordância entre os *experts,* as equivalências foram medidas por meio do IVC^(19)^. Considerou-se o valor mínimo de IVC 0,80, ou 80,0%, por item. Os itens que não alcançaram o valor mínimo foram submetidos à reavaliação em segunda rodada por meio da técnica Delphi até obtenção de consenso pelo comitê.

O alfa de Cronbach foi calculado para análise da consistência interna na primeira e segunda rodada de avaliação dos *experts*.

Em relação à análise de conteúdo na avaliação global do instrumento, calculou-se a Razão de Validade de Conteúdo (RVC), que mede a concordância entre os enfermeiros avaliadores sobre a importância do item avaliado^(20-21)^. No presente estudo, houve a participação de 44 enfermeiros, sendo necessário um valor mínimo de RVC de 0,29^(21-22)^.

## RESULTADOS

As versões TA e TB alcançaram resultados próximos e TS foi finalizada após ajustes. RTA e RTB indicaram fidelidade ao conteúdo original e, então, TS foi encaminhada para análise por especialistas. Em relação ao título do instrumento, decidiu-se pela manutenção em inglês, acrescida pela designação da tradução como “*MISSCARE Survey-Ped* Brasil”.

Considerando os itens dos instrumentos, títulos e subtítulos, foram analisados 50 itens. Na primeira rodada de avaliação pelo comitê de especialistas, cinco (10,0%) e sete (14,0%) itens relativos às Seções A e B, respectivamente, foram modificados segundo sugestões dos *experts*, pois obtiveram IVC<80,0%. Os demais itens obtiveram IVC > 80,0%, e o IVC do instrumento na primeira rodada foi de 89,0%, conforme demonstrado nas Tabelas 1 e 2. O alfa de Cronbach da primeira rodada foi 0,826, indicando uma boa consistência.

**Tabela 1:** Primeira rodada da técnica Delphi da avaliação da tradução da Seção A do instrumento *MISSCARE Survey-Ped* para o português do Brasil pelo comitê de especialistas segundo equivalência semântica, equivalência idiomática, equivalência experimental, equivalência cultural

Itens analisados	Pontuação, média IVC^*^ (%)				
	ES	EI	EE	EC	Total
Seção “A” - Atividades de cuidados de enfermagem não realizadas	4(80,0)	3(60,0)	2(40,0)	2(60,0)	2,75(55,0)
Participação na visita clínica diária à beira leito	3(60,0)	3(60,0)	3(60,0)	3(60,0)	3(60,0)
Deambulação 3 vezes ao dia ou de acordo com o plano de cuidados de enfermagem, se as condições clínicas permitirem	5(100,0)	5(100,0)	4(80,0)	5(100,0)	4,75(95,0)
Avaliação da eficácia da medicação	4(80,0)	4(80,0)	5(100,0)	5(100,0)	4,5(90,0)
Mudança de decúbito da criança a cada 2 horas ou conforme prescrito	5(100,0)	5(100,0)	5(100,0)	5(100,0)	5(100,0)
Cuidados orais	3(60,0)	3(60,0)	2(40,0)	4(80,0)	3(60,0)
Envolvimento dos pais nos cuidados com a criança	4(80,0)	4(80,0)	4(80,0)	4(80,0)	4(80,0)
Educação do paciente e família	5(100,0)	5(100,0)	4(80,0)	5(100,0)	4,75(95,0)
Discussão com a criança e sua família sobre planos de alta e cuidados no domicílio	5(100,0)	5(100,0)	5(100,0)	5(100,0)	5(100,0)
Promoção do desenvolvimento neuroevolutivo, de acordo com a idade e condições clínicas da criança (por exemplo, cuidados neonatais, desenvolvimento cognitivo e relacional da criança ou do adolescente)	5(100,0)	4(80,0)	4(80,0)	5(100,0)	4,5(90,0)
Avaliação da dor e intervenções farmacológicas ou não farmacológicas, de acordo com protocolos	5(100,0)	5(100,0)	5(100,0)	5(100,0)	5(100,0)
Solicitações de medicamentos atendidas dentro de 15 minutos	5(100,0)	5(100,0)	5(100,0)	5(100,0)	5(100,0)
Documentação completa com todos os dados necessários	3(60,0)	5(100,0)	5(100,0)	5(100,0)	4,5(90,0)
Comunicação de todas as informações relevantes na passagem de plantão ou transferência	5(100,0)	5(100,0)	5(100,0)	5(100,0)	5(100,0)
Satisfação das necessidades alimentares, de acordo com as condições clínicas da criança (por exemplo, incentivo a alimentação oral e/ou nutrição do recém-nascido assim que solicitado; incentivo a alimentação apropriada, de acordo com a preferência pessoal)	5(100,0)	5(100,0)	5(100,0)	5(100,0)	5(100,0)
Administração de medicamentos 30 minutos antes ou depois do horário programado (por exemplo, horário programado às 20h, administração entre 19h30 e 20h30)	5(100,0)	4(80,0)	4(80,0)	5(100,0)	4,5(90,0)
Auxílio a criança nas necessidades de eliminação dentro de 5 minutos após a solicitação (por exemplo, ir com a criança ao banheiro ou fornecer os dispositivos apropriados se estiver no leito)	4(80,0)	3(60,0)	4(80,0)	4(80,0)	3,75(75,0)
Resposta à luz de chamada, à solicitação de intervenção ou alarme é iniciada dentro de 5 minutos (por exemplo, monitores, bombas de infusão, aparelhos de ventilação mecânica)	5(100,0)	4(80,0)	4(80,0)	5(100,0)	4,5(90,0)
Apoio emocional à criança e/ou família	5(100,0)	5(100,0)	5(100,0)	5(100,0)	5(100,0)
Coleta de exames laboratoriais realizados conforme prescrito	5(100,0)	5(100,0)	5(100,0)	5(100,0)	5(100,0)
Higiene corporal e cuidados com a pele	5(100,0)	5(100,0)	5(100,0)	5(100,0)	5(100,0)
Avaliação do local de inserção do cateter intravenosos central e do cateter intravenoso periférico segundo protocolos	4(80,0)	4(80,0)	4(80,0)	5(100,0)	4,25(85,0)
Cuidados com o local de inserção do cateter intravenoso central e do cateter intravenoso periférico segundo protocolos	4(80,0)	4(80,0)	4(80,0)	5(100,0)	4,25(85,0)
Adoção das precauções necessárias para o controle de infecções conforme protocolos (uso de EPIs, desinfecção de dispositivos, isolamento, correto descarte de resíduos)	5(100,0)	4(80,0)	4(80,0)	5(100,0)	4,5(90,0)
Monitoramento dos ganhos e perdas de sólidos e líquidos	5(100,0)	5(100,0)	4(80,0)	5(100,0)	4,75(95,0)
Avaliação dos sinais vitais de acordo com o plano de cuidados de enfermagem	5(100,0)	5(100,0)	5(100,0)	5(100,0)	5(100,0)
Reavaliações direcionadas sobre a condição da criança para avaliar melhorias ou agravos durante o plantão	5(100,0)	4(80,0)	4(80,0)	5(100,0)	4,5(90,0)
Higienização das mãos	5(100,0)	5(100,0)	5(100,0)	5(100,0)	5(100,0)
Avaliação das atividades realizadas pelo cuidador	4(80,0)	3(60,0)	3(60,0)	4(80,0)	3,5(70,0)
Verificação de segurança dos equipamentos e limpeza concorrente do mobiliário realizadas uma vez por plantão ou segundo protocolo (por exemplo, cama, mesa de cabeceira, dispositivos)	5(100,0)	4(80,0)	5(100,0)	5(100,0)	4,75(95,0)

* *IVC - Índice de Validade de Conteúdo; ES - equivalência semântica; EI - equivalência idiomática; EE - equivalência experimental; EC - equivalência cultural; EPI - equipamento de proteção individual.*

**Tabela 2:** Primeira rodada da técnica Delphi da avaliação da tradução da Seção B do instrumento *MISSCARE Survey-Ped* para o português do Brasil pelo comitê de especialistas segundo equivalência semântica, equivalência idiomática, equivalência experimental, equivalência cultural

Itens analisados	Pontuação, média IVC^*^ (%)				
	ES	EI	EE	EC	Total
Seção “B” - razões para as omissões de cuidados em enfermagem	5(100,0)	5(100,0)	5(100,0)	5(100,0)	5(100,0)
Recursos laborais	5(100,0)	4(80,0)	4(80,0)	4(80,0)	4,25(85,0)
Desequilíbrio nas atribuições com pacientes	5(100,0)	5(100,0)	4(80,0)	4(80,0)	4,5(90,0)
Número inadequado de enfermeiras	3(60,0)	4(80,0)	4(80,0)	4(80,0)	3,75(75,0)
Situação de urgência do paciente (por exemplo, piora da condição do paciente)	5(100,0)	4(80,0)	4(80,0)	4(80,0)	4,25(85,0)
Aumento inesperado do número e/ou gravidade dos pacientes na unidade	5(100,0)	5(100,0)	5(100,0)	5(100,0)	5(100,0)
Número inadequado de técnicos/auxiliares de enfermagem	5(100,0)	5(100,0)	5(100,0)	5(100,0)	5(100,0)
Interrupções frequentes	5(100,0)	5(100,0)	5(100,0)	5(100,0)	5(100,0)
Comunicação	5(100,0)	5(100,0)	5(100,0)	5(100,0)	5(100,0)
Tensão ou barreiras de comunicação com a equipe médica	4(80,0)	3(60,0)	3(60,0)	4(80,0)	3,5(70,0)
Falta de colaboração entre membros da equipe (por exemplo, enfermeiras, técnicas/auxiliares de enfermagem e médicos)	3(60,0)	4(80,0)	4(80,0)	4(80,0)	3,75(75,0)
Tensão ou barreiras de comunicação na equipe de enfermagem	4(80,0)	4(80,0)	3(60,0)	4(80,0)	3,75(75,0)
Tensão ou barreiras de comunicação com outros serviços ou departamentos (por exemplo, banco de sangue, serviço de radiologia, farmácia, etc)	4(80,0)	4(80,0)	1(20,0)	4(80,0)	3,25(65,0)
Técnica/auxiliar de enfermagem não comunicou que o cuidado à criança não foi realizado	3(60,0)	4(80,0)	4(80,0)	4(80,0)	3,75(75,0)
Inadequada passagem de plantão entre turnos ou na transferência entre unidades	5(100,0)	5(100,0)	5(100,0)	5(100,0)	5(100,0)
Outros serviços ou setores não prestaram os cuidados necessários (por exemplo, laboratório de análises, farmácia hospitalar)	5(100,0)	5(100,0)	3(60,0)	5(100,0)	4,5(90,0)
Recursos materiais	5(100,0)	5(100,0)	5(100,0)	5(100,0)	5(100,0)
Materiais/equipamentos não disponíveis quando necessários (por exemplo, bombas de infusão, instrumentais cirúrgicos)	5(100,0)	5(100,0)	5(100,0)	5(100,0)	5(100,0)
Materiais/equipamentos não funcionam corretamente quando necessário	5(100,0)	5(100,0)	5(100,0)	5(100,0)	5(100,0)
Medicamentos não disponíveis quando necessários	5(100,0)	5(100,0)	5(100,0)	5(100,0)	5(100,0)
Falta de treinamento com equipamento/procedimento/normas	3(60,0)	4(80,0)	4(80,0)	4(80,0)	3,75(75,0)

* *IVC - Índice de Validade de Conteúdo; ES - equivalência semântica; EI - equivalência idiomática; EE - equivalência experimental; EC - equivalência cultural.*

Dos 12 itens que precisaram de revisão após a primeira rodada, todos foram validados na segunda rodada da análise, e o IVC final do instrumento foi de 0,95. O alfa de Cronbach da segunda rodada foi 0,804, indicando uma consistência aceitável apesar do decaimento de uma rodada para outra.

Após a análise por especialistas, o pré-teste foi aplicado com 44 enfermeiros. Todos os participantes eram do sexo feminino, com média de idade de 40 anos, atuantes em diferentes áreas e regiões do país e com diferentes níveis de formação (Tabela 3).

**Tabela 3:** Dados coletados no pré-teste com a caracterização dos participantes

Área de atuação	n(%)	Região pertencente	n(%)	Nível de formação	n(%)
Assistência hospitalar	29(65,9%)	Norte	0(0%)	Doutorado	14(31,8%)
Docência	26(59,0%)	Nordeste	6(13,6%)	Especialização/aperfeiçoamento	13(29,5%)
Pesquisa	23(52,2%)	Centro-Oeste	2(4,5%)	Mestrado	10(22,7%)
Atenção primária	5(11,3%)	Sudeste	32(72,7%)	Pós-doutorado	5(11,4%)
Gestão	6(13,6%)	Sul	4(9,1%)	Livre-Docência	1(2,3%)
Outros	2(4,5%)			Graduação	1(2,3%)

Portanto, 30 enfermeiros (68,1%) trabalham em instituição pública; nove (20,4%) trabalham em instituição privada; quatro (9,0%) trabalham em ambas; e um (2,2%) se identificou como aposentado/não empregado. A maioria (68,1%) dos participantes trabalha no período diurno e 75,0% dos enfermeiros não referiram dupla jornada de trabalho. No tocante à jornada diária, 21 (47,7%) trabalham oito horas por dia; nove (20,0%) assinalaram a opção outro ou não se aplica; sete (15,9%) cumprem a escala 12x36; e sete (15,9%) trabalham seis horas ou menos.

Sobre a satisfação no trabalho, 33 (75,0%) enfermeiros declararam estar satisfeitos; nove (20,4%) estão muito satisfeitos; e dois (4,5%) estão pouco satisfeitos. Assim, 20 (45,5%) participantes assinalaram que se sentem mais feliz/animado(a)/bem-humorado(a) do que triste/desanimado(a)/mal-humorado(a) no seu trabalho; quatro (9,1%) enfermeiros se sentem mais triste/desanimado(a)/mal-humorado(a) do que feliz/animado(a)/bem-humorado(a); 23 (52,3%) se sentem mais tranquilo(a)/satisfeito(a) do que tenso(a)/estressado(a); 10 (22,7%) se sentem mais tenso(a)/estressado(a) do que tranquilo(a)/satisfeito(a).

Após a apresentação do instrumento, 42 (95,5%) dos enfermeiros identificaram cuidados de enfermagem não realizados, dispostos na Seção A. Os itens mais assinalados foram “Verificação de segurança dos equipamentos e limpeza concorrente do mobiliário realizadas uma vez por plantão ou segundo protocolo (por exemplo, cama, mesa de cabeceira, dispositivos)”, assinalado 29 (69,0%) vezes, “Participação na visita clínica multiprofissional diária à beira leito”, assinalado 27 (64,3%) vezes, “Cuidados bucais”, “Solicitações de medicamentos atendidas dentro de 15 minutos” e “Avaliação das atividades atribuídas aos cuidadores”, assinalados 22 (52,4%) vezes cada.

Na Seção B, 43 (97,7%) enfermeiros responderam que as razões para as omissões de cuidados de enfermagem estavam relacionadas a recursos laborais. Os itens mais assinalados foram “Número inadequado de técnicos/auxiliares de enfermagem”, com 34 (79,1%) respostas, e “Número inadequado de enfermeiros”, com 33 (76,7%) respostas.

Sobre as causas relacionadas à comunicação, 41 (93,2%) enfermeiros responderam e assinalaram principalmente os itens “Falta de colaboração entre membros da equipe (por exemplo, enfermeiros, técnicos/auxiliares de enfermagem e médicos)”, com 33 (80,5%) respostas, e “Inadequada passagem de plantão entre turnos ou na transferência entre unidades”, com 28 (68,3%) respostas.

Assim, 40 (90,9%) enfermeiros consideraram recursos materiais como motivo para a omissão de cuidados de enfermagem, assinalando em maior número os itens “Materiais/equipamentos não funcionam corretamente quando necessário”, com 29 (72,5%) respostas, e “Materiais/equipamentos não disponíveis quando necessários (por exemplo, bombas de infusão, instrumentais cirúrgicos)”, com 28 (70,0%) respostas.

Em relação à compreensão, 29 (65,9%) dos enfermeiros concordaram totalmente que o instrumento é claro, inteligível e de fácil compreensão; 10 (22,7%) concordaram parcialmente; quatro (9,1%) discordaram parcialmente; e um (2,3%) não concordou nem discordou.

Na avaliação da pertinência, 28 (63,6%) enfermeiros concordaram totalmente que o instrumento abrange situações relacionadas ao cuidado de enfermagem não realizado e as razões para omissões; 13 (29,5%) concordaram parcialmente; dois (4,5%) não concordaram nem discordaram; e um (2,3%) discordou totalmente.

Obteve-se que 32 (72,7%) enfermeiros concordaram totalmente na análise de que o instrumento é relevante para a identificação de cuidados de enfermagem não realizados e as razões para omissões; 11 (25,0%) concordaram parcialmente; e um (2,3%) discordou parcialmente.

Conforme a Tabela 4, notam-se altos valores de RVC e IVC, indicando a consistência nas respostas dos avaliadores para os itens de avaliação do questionário.

**Tabela 4:** Estimativas do Razão de Validade de Conteúdo e Índice de Validade de Conteúdo na avaliação global do *MISSCARE Survey-Ped* Brasil quanto à compreensão, pertinência e relevância

	O instrumento é claro, inteligível e de fácil compreensão	O instrumento abrange situações relacionadas ao cuidado de enfermagem não realizado e as razões para omissões	O instrumento é relevante para a identificação de cuidados de enfermagem não realizados e as razões para omissões
RVC^*^	0,773	0,864	0,955
IVC^†^	0,886	0,931	0,977

* RVC - Razão de Validade de Conteúdo;

† IVC - Índice de Validade de Conteúdo.

A partir da análise estatística utilizando o modelo linear generalizado Quase-Poisson, observou-se que os recursos laborais aumentaram em média 1.290 cuidados de enfermagem descritos como não realizados, visto que houve relação significante entre apontar cuidados de enfermagem não realizados por causas laborais (p<0,001).

Também verificou-se que o total de cuidados de enfermagem apontados na Seção A não está relacionado com as variáveis sociodemográficas. A quantidade média de razões assinaladas na Seção B é diferente entre quem possui jornada de seis horas ou menos e quem respondeu outro/não se aplica. Enfermeiros que responderam outro/não se aplica assinalaram mais motivos na Seção B.

Trabalhar no período noturno aumentou em média 1,6 a quantidade de itens assinalados na Seção B em recursos laborais. Estar mais feliz/animado(a)/bem-humorado(a) do que triste/desanimado(a)/mal-humorado(a) e mais tranquilo(a)/satisfeito(a) do que tenso(a)/estressado(a) diminuiu em média 1,37 a quantidade de itens assinalados na Parte B em recursos laborais.

No processo de adaptação transcultural, destacaram-se sugestões relativas aos termos utilizados. Um enfermeiro mencionou que “*No item “Resposta à luz de chamada, à solicitação de chamada e alarme”, luz de chamada talvez seja mais compreensível como campainha [...]”*. Outra sugestão foi *“No item “Documentação completa com todos os dados necessários”, não está muito claro na tradução se isso se refere a documentos do prontuário ou passagem de plantão ou transição do cuidado [...]*”. Porém, os referidos itens não sofreram revisões, visto que foram citados por apenas um enfermeiro (2,2%).

A partir das sugestões dos enfermeiros participantes sobre a Seção A, Seção B e avaliação global do instrumento, o item “Desequilíbrio nas atribuições com pacientes” precisou ser revisado e alterado para “Desequilíbrio no dimensionamento de pacientes”, visto que foi citada tal questão por dois enfermeiros (4,5%) que expressaram dúvida quanto ao significado da frase, sendo entendida como pertinente a sugestão. Dessa forma, finalizou-se a última versão do *MISSCARE Survey-Ped* Brasil, como apresentado no Quadro 1.

**Quadro 1:** Versões do *MISSCARE Survey-Ped* Brasil com os itens alterados segundo sugestões destacados em negrito

Versão inicial do *MISSCARE Survey-Ped* Brasil	Versão final do *MISSCARE Survey-Ped* Brasil	Análise das modificações
**Seção “A” - Atividades de cuidados de enfermagem não realizadas**	**Seção “A” - Cuidados de enfermagem não realizados**	*A palavra “atividades” foi suprimida pois “atividades de” seguida por “cuidados” foi considerado redundante.*
**Participação na visita clínica diária à beira leito**	**Participação na visita clínica multiprofissional diária à beira leito**	*O item foi reformulado pois não ficou claro se o termo “visita” era referente ao comparecimento do enfermeiro ao quarto do paciente em uma visita clínica multiprofissional, no contexto da passagem de caso, ou se o enfermeiro não adentrou o quarto do paciente durante todo o plantão.*
Deambulação 3 vezes ao dia ou de acordo com o plano de cuidados de enfermagem, se as condições clínicas permitirem	Deambulação 3 vezes ao dia ou de acordo com o plano de cuidados de enfermagem, se as condições clínicas permitirem	
Avaliação da eficácia da medicação	Avaliação da eficácia da medicação	
Mudança de decúbito da criança a cada 2 horas ou conforme prescrito	Mudança de decúbito da criança a cada 2 horas ou conforme prescrito	
**Cuidados orais**	**Cuidados bucais**	*A palavra “orais” foi substituída por “bucais” pois este está mais próximo do que é utilizado na prática de enfermagem.*
Envolvimento dos pais nos cuidados com a criança	Envolvimento dos pais nos cuidados com a criança	
Educação do paciente e família	Educação do paciente e família	
Discussão com a criança e sua família sobre planos de alta e cuidados no domicílio	Discussão com a criança e sua família sobre planos de alta e cuidados no domicílio	
Promoção do desenvolvimento neuroevolutivo, de acordo com a idade e condições clínicas da criança (por exemplo, cuidados neonatais, desenvolvimento cognitivo e relacional da criança ou do adolescente)	Promoção do desenvolvimento neuroevolutivo, de acordo com a idade e condições clínicas da criança (por exemplo, cuidados neonatais, desenvolvimento cognitivo e relacional da criança ou do adolescente)	
Avaliação da dor e intervenções farmacológicas ou não farmacológicas, de acordo com protocolos	Avaliação da dor e intervenções farmacológicas ou não farmacológicas, de acordo com protocolos	
Solicitações de medicamentos atendidas dentro de 15 minutos	Solicitações de medicamentos atendidas dentro de 15 minutos	
Documentação completa com todos os dados necessários	Documentação completa com todos os dados necessários	
Comunicação de todas as informações relevantes na passagem de plantão ou transferência	Comunicação de todas as informações relevantes na passagem de plantão ou transferência	
Satisfação das necessidades alimentares, de acordo com as condições clínicas da criança (por exemplo, incentivo a alimentação oral e/ou nutrição do recém-nascido assim que solicitado; incentivo a alimentação apropriada, de acordo com a preferência pessoal)	Satisfação das necessidades alimentares, de acordo com as condições clínicas da criança (por exemplo, incentivo a alimentação oral e/ou nutrição do recém-nascido assim que solicitado; incentivo a alimentação apropriada, de acordo com a preferência pessoal)	
Administração de medicamentos entre 30 minutos antes ou depois do horário programado (por exemplo, horário programado às 20h, administração entre 19h30 e 20h30)	Administração de medicamentos entre 30 minutos antes ou depois do horário programado (por exemplo, horário programado às 20h, administração entre 19h30 e 20h30)	
**Auxílio a criança nas necessidades de eliminação dentro de 5 minutos após a solicitação (por exemplo, ir com a criança ao banheiro ou fornecer os dispositivos apropriados se estiver no leito)**	**Auxílio a criança nas necessidades de eliminação dentro de 5 minutos após a solicitação (por exemplo, ir com a criança ao banheiro ou fornecer os dispositivos apropriados se estiver restrita no leito)**	*Foi acrescentado o termo “restrita no leito”, uma vez que é subentendido que todas as crianças hospitalizadas estarão em um leito.*
Resposta à luz de chamada, à solicitação de intervenção ou alarme é iniciada dentro de 5 minutos (por exemplo, monitores, bombas de infusão, aparelhos de ventilação mecânica)	Resposta à luz de chamada, à solicitação de intervenção ou alarme é iniciada dentro de 5 minutos (por exemplo, monitores, bombas de infusão, aparelhos de ventilação mecânica)	
Apoio emocional à criança e/ou família	Apoio emocional à criança e/ou família	
Coleta de exames laboratoriais realizados conforme prescrito	Coleta de exames laboratoriais realizados conforme prescrito	
Higiene corporal e cuidados com a pele	Higiene corporal e cuidados com a pele	
Avaliação do local de inserção do cateter intravenosos central e do cateter intravenoso periférico segundo protocolos	Avaliação do local de inserção do cateter intravenosos central e do cateter intravenoso periférico segundo protocolos	
Cuidados com o local de inserção do cateter intravenoso central e do cateter intravenoso periférico segundo protocolos	Cuidados com o local de inserção do cateter intravenoso central e do cateter intravenoso periférico segundo protocolos	
Adoção das precauções necessárias para o controle de infecções conforme protocolos (uso de EPIs, desinfecção de dispositivos, isolamento, correto descarte de resíduos)	Adoção das precauções necessárias para o controle de infecções conforme protocolos (uso de EPIs, desinfecção de dispositivos, isolamento, correto descarte de resíduos)	
Monitoramento dos ganhos e perdas de sólidos e líquidos	Monitoramento dos ganhos e perdas de sólidos e líquidos	
Avaliação dos sinais vitais de acordo com o plano de cuidados de enfermagem	Avaliação dos sinais vitais de acordo com o plano de cuidados de enfermagem	
Reavaliações direcionadas sobre a condição da criança para avaliar melhorias ou agravos durante o plantão	Reavaliações direcionadas sobre a condição da criança para avaliar melhorias ou agravos durante o plantão	
Higienização das mãos	Higienização das mãos	
**Avaliação das atividades realizadas pelo cuidador**	**Avaliação das atividades atribuídas aos cuidadores**	*Visto que “realizar” e “atribuir” são conceitos distintos, uma análise foi feita com a versão original do instrumento, entendendo-se como mais pertinente ao contexto o uso da palavra “atribuídas”.*
Verificação de segurança dos equipamentos e limpeza concorrente do mobiliário realizadas uma vez por plantão ou segundo protocolo (por exemplo, cama, mesa de cabeceira, dispositivos)	Verificação de segurança dos equipamentos e limpeza concorrente do mobiliário realizadas uma vez por plantão ou segundo protocolo (por exemplo, cama, mesa de cabeceira, dispositivos)	
Seção “B” - razões para as omissões de cuidados em enfermagem	Seção “B” - razões para as omissões de cuidados em enfermagem	
Recursos laborais	Recursos laborais	
**Desequilíbrio nas atribuições com** **pacientes**	**Desequilíbrio no dimensionamento de pacientes**	*“Atribuições” não ficou claro e por isso foi substituído para “dimensionamento”.*
**Número inadequado de enfermeiras**	**Número inadequado de enfermeiros**	*Modificou-se para “enfermeiros” para englobar ambos os sexos.*
Situação de urgência do paciente (por exemplo, piora da condição do paciente)	Situação de urgência do paciente (por exemplo, piora da condição do paciente)	
Aumento inesperado do número e/ou gravidade dos pacientes na unidade	Aumento inesperado do número e/ou gravidade dos pacientes na unidade	
Número inadequado de técnicos/auxiliares de enfermagem	Número inadequado de técnicos/auxiliares de enfermagem	
Interrupções frequentes	Interrupções frequentes	
Comunicação	Comunicação	
**Tensão ou barreiras de comunicação com a equipe médica**	**Tensão ou falhas na comunicação com a equipe médica**	*A palavra “barreiras” não ficou clara e por isso foi substituída para “falhas”.*
**Falta de colaboração entre membros da equipe (por exemplo, enfermeiras, técnicas/auxiliares de** **enfermagem e médicos)**	**Falta de colaboração entre membros da equipe (por exemplo, enfermeiros, técnicas/auxiliares de enfermagem e médicos)**	*Modificou-se para “enfermeiros” e “técnicos” para englobar ambos os sexos.*
**Tensão ou barreiras de comunicação na equipe de enfermagem**	**Tensão ou falhas na comunicação na equipe de enfermagem**	*A palavra “barreiras” não ficou clara e por isso foi substituída para “falhas”.*
**Tensão ou barreiras de comunicação com outros serviços ou departamentos (por exemplo, banco de sangue, serviço de radiologia, farmácia, etc)**	**Tensão ou falhas na comunicação com outros serviços ou departamentos (por exemplo, banco de sangue, serviço de radiologia, farmácia, etc)**	*Idem acima.*
**Técnica/auxiliar de enfermagem não comunicou que o cuidado à criança não foi realizado**	**Técnico/auxiliar de enfermagem não comunicou que o cuidado à criança não foi realizado**	*Modificou-se para “técnicos” para englobar ambos os sexos.*
Inadequada passagem de plantão entre turnos ou na transferência entre unidades	Inadequada passagem de plantão entre turnos ou na transferência entre unidades	
Outros serviços ou setores não prestaram os cuidados necessários (por exemplo, laboratório de análises, farmácia hospitalar)	Outros serviços ou setores não prestaram os cuidados necessários (por exemplo, laboratório de análises, farmácia hospitalar)	
Recursos materiais	Recursos materiais	
Materiais/equipamentos não disponíveis quando necessários (por exemplo, bombas de infusão, instrumentais cirúrgicos)	Materiais/equipamentos não disponíveis quando necessários (por exemplo, bombas de infusão, instrumentais cirúrgicos)	
Materiais/equipamentos não funcionam corretamente quando necessário	Materiais/equipamentos não funcionam corretamente quando necessário	
Medicamentos não disponíveis quando necessários	Medicamentos não disponíveis quando necessários	
**Falta de treinamento com equipamento/procedimento/norma**	**Falta de familiaridade com equipamento/procedimento/norma**	*O termo “treinamento” foi substituído por “familiaridade”, pois, embora o profissional possa ter sido treinado, ele pode não ter muitas oportunidades de contato com algum equipamento/procedimento/ norma.*

## DISCUSSÃO

Os erros de omissão são considerados fenômenos intrínsecos ao processo cognitivo humano e estão entre os tipos de incidentes mais relevantes no sistema de saúde em todos o mundo, devido à sua ocorrência frequente e potencial de oferecer dano ao paciente. Contudo, ainda existem poucas evidências empíricas desse fenômeno em populações pediátricas^(23)^.

O presente estudo descreveu o processo de tradução e adaptação transcultural do instrumento *MISSCARE Survey-Ped*, originalmente na língua inglesa, para a língua portuguesa do Brasil, seguindo as recomendações preconizadas pela literatura. O processo, embora complexo, é de grande importância, porque, enquanto preserva as características da versão original, é adaptada considerando a heterogeneidade da população e a utilização de termos regionais^(24)^.

O envolvimento de especialistas em enfermagem pediátrica e segurança do paciente favoreceu o desenvolvimento do estudo, visto que, com sua comprovada experiência na área, sugeriram ajustes que promoveram melhorias da versão síntese (TS). Da mesma forma, a participação de enfermeiros pediatras de todas as regiões do Brasil, exceto da região Norte, com diferentes níveis de formação, beneficiou a identificação das diferenças nos aspectos da prática de enfermagem, ao considerar as proporções continentais do país. Destaca-se, contudo, que a maioria dos enfermeiros possuía formação acadêmica de pós-graduação, refletindo uma parcela de enfermeiros com maior preparo acadêmico. Tal resultado pode ter relação com a baixa proporção de enfermeiros que aceitou participar da pesquisa, estando os profissionais pós-graduados mais estimulados para a participação em pesquisas científicas dessa natureza.

A relação entre a identificação de cuidados não realizados em pacientes adultos observados no estudo de validação do *MISSCARE* no Brasil^(10)^, em comparação com pediátricos, é significativa no que tange à participação na visita clínica multiprofissional, como também foi observado no presente estudo. A organização do processo de trabalho da equipe multiprofissional no atendimento à criança hospitalizada depende das concepções dos profissionais acerca do significado de trabalho e equipe e da sua importância, visando ao alcance de uma assistência integral. As falhas de comunicação, a sobrecarga de trabalho, os fluxos e processos dos serviços de saúde e as interrupções são aspectos que fragilizam a dinâmica assistencial^(25)^.

Em relação aos cuidados não realizados no contexto pediátrico, no estudo de validação do *MISSCARE Survey-Ped* para uso na Turquia, as principais atividades de cuidados de enfermagem não realizadas durante o último plantão foram: administrar os medicamentos entre 30 minutos antes ou depois do horário programado; atender solicitações de medicamentos prescritos ao paciente dentro de 15 minutos; e envolver os pais nos cuidados com a criança^(26)^. Esse último, sendo um cuidado específico do ambiente pediátrico, aponta para o Cuidado Centrado no Paciente e na Família (CCPF).

A implementação do CCPF nos contextos de cuidado pode ter como desdobramento diversas ações e estratégias que contribuam com a segurança do paciente, promovendo a prestação de um cuidado qualificado que resulte em uma melhor experiência do paciente e família no contato com o sistema de saúde. No entanto, a falta de treinamento dos profissionais de saúde sobre o estabelecimento de relacionamentos eficazes com o paciente ainda é um obstáculo a ser superado^(9,25)^.

Destaca-se, ainda, o item da Seção A, que foi mais assinalado pelos enfermeiros brasileiros participantes com o cuidado não realizado, “Verificação de segurança dos equipamentos e limpeza concorrente do mobiliário realizadas uma vez por plantão ou segundo protocolo (por exemplo, cama, mesa de cabeceira, dispositivos)”, que está diretamente ligado à prevenção e controle de infecção hospitalar. As Infecções Relacionadas à Assistência à Saúde (IRAS) em pediatria são importantes eventos adversos que afetam o tratamento, aumentando a morbidade, a mortalidade, o tempo de internação, os custos e o sofrimento da criança e família.

Na Seção B, que aponta para as razões da omissão de cuidados de enfermagem, a subseção “recursos laborais” apresentou maior comparação média de respostas. Da mesma forma, os estudos realizados com o *MISSCARE* nos Estados Unidos^(12)^, Turquia^(27)^ e Brasil^(10)^ também obtiveram maiores pontuações relacionadas aos recursos laborais.

O número inadequado de profissionais de enfermagem foi citado como a principal razão para a não realização de cuidados de enfermagem em pediatria. Isso condiz com estudos nacionais que relatam as precariedades, inadequações e insatisfações vividas nos hospitais por esses profissionais envolvendo condições de trabalho, insatisfação com o salário, aumento de horas trabalhadas e *burnout*. Como consequência, os trabalhadores são levados a solicitar afastamentos e/ou pedir demissão, situação que contribui para a falta de profissionais na área^(28-30)^. Considerando que a maioria dos enfermeiros participantes atua assistencialmente em instituições públicas de saúde, explica-se o número alto de enfermeiros que assinalaram esse item.

Ambas as versões, *MISSCARE Survey-Ped* e *MISSCARE Survey-Ped* Brasil, apresentaram resultados similares no processo de validação: os valores de alfa de Cronbach foram maiores que 0,70, considerados aceitáveis para a consistência interna e, da mesma forma, o IVC foi maior que 0,80, evidenciando boa validade de conteúdo.

### Limitações do estudo

Foi identificada como uma limitação deste estudo a impossibilidade de comparar os achados entre todas as regiões brasileiras, visto que não houve participação de enfermeiros da região Norte. Além disso, o contexto de prática de enfermagem dos participantes pode modificar sua avaliação quanto às situações que acarretam omissões de cuidados de enfermagem. Embora realizado em diferentes regiões do país, como o local de trabalho não foi uma variável considerada neste estudo, enfermeiros alocados na determinada localidade podem ter contribuído de maneira mais ampla, sem necessariamente refletir a realidade de tal região. Considerando que este foi um estudo de tradução e adaptação transcultural, é necessário ainda que o *MISSCARE Survey-Ped* Brasil seja aplicado para maior amostra e com análise fatorial. Este estudo está em desenvolvimento para avaliação prática do instrumento.

### Contribuições para a área da enfermagem, saúde, ou políticas públicas

As vantagens da utilização do instrumento são amplamente descritas em literatura internacional, destacando-se que pode oferecer informações para o desenvolvimento de políticas públicas voltadas à promoção da segurança do paciente, ao ter sido avaliada por especialistas. Também pode ser útil às instituições de saúde na elaboração de protocolos, diretrizes ou *checklists*, a fim de promover ambientes positivos de prática profissional, prevenir esquecimentos e possíveis erros de omissão durante a prática de enfermagem. Por ser direcionado à enfermagem pediátrica, abrangerá especificidades dessa população, possibilitando melhorar indicadores de resultados na atenção à saúde da criança que requer hospitalização.

## CONCLUSÕES

Com este estudo, foi possível realizar a tradução e adaptação transcultural para a língua portuguesa do Brasil do instrumento *MISSCARE Survey-Ped*, sendo considerado apto para uso em instituições de saúde nacionais, ao alcançar níveis adequados de validação do conteúdo por especialistas e por enfermeiros na prática clínica.

## Data Availability

https://doi.org/10.48331/scielodata.IOBMLO
